# Quaternary Ammonium Compounds (QACs) and Ionic Liquids (ILs) as Biocides: From Simple Antiseptics to Tunable Antimicrobials

**DOI:** 10.3390/ijms22136793

**Published:** 2021-06-24

**Authors:** Anatoly N. Vereshchagin, Nikita A. Frolov, Ksenia S. Egorova, Marina M. Seitkalieva, Valentine P. Ananikov

**Affiliations:** N. D. Zelinsky Institute of Organic Chemistry, Russian Academy of Sciences, Leninsky Prospect 47, 119991 Moscow, Russia; nikitafrolov298@gmail.com (N.A.F.); egorova-ks@ioc.ac.ru (K.S.E.); s_marina@ioc.ac.ru (M.M.S.)

**Keywords:** quaternary ammonium compound, ionic liquid, antibacterial, antimicrobial, biocide

## Abstract

Quaternary ammonium compounds (QACs) belong to a well-known class of cationic biocides with a broad spectrum of antimicrobial activity. They are used as essential components in surfactants, personal hygiene products, cosmetics, softeners, dyes, biological dyes, antiseptics, and disinfectants. Simple but varied in their structure, QACs are divided into several subclasses: Mono-, bis-, multi-, and poly-derivatives. Since the beginning of the 20th century, a significant amount of work has been dedicated to the advancement of this class of biocides. Thus, more than 700 articles on QACs were published only in 2020, according to the modern literature. The structural variability and diverse biological activity of ionic liquids (ILs) make them highly prospective for developing new types of biocides. QACs and ILs bear a common key element in the molecular structure–quaternary positively charged nitrogen atoms within a cyclic or acyclic structural framework. The state-of-the-art research level and paramount demand in modern society recall the rapid development of a new generation of tunable antimicrobials. This review focuses on the main QACs exhibiting antimicrobial and antifungal properties, commercial products based on QACs, and the latest discoveries in QACs and ILs connected with biocide development.

## 1. Introduction

For many years, quaternary ammonium compounds (QACs) have been included in most antiseptics and disinfectants and used in various areas, from household and agriculture to medicine and industry [1].

The COVID-19 pandemic that broke out in 2020 led to a significant increase in the widespread use of sanitizers, including QACs. Recent studies have shown that more than 90% of the dust samples analyzed during the pandemic contained QACs, and their average concentration doubled compared to the pre-COVID period [2]. It is to be expected that with the further progression of the pandemic, this number will increase, although the virucidal effect of QACs on SARS-CoV-2 requires further research [3].

The constant presence of subinhibitory concentrations of QACs on various working surfaces, together with the frequent use of QACs, increases the risk of the development of a resistant bacterial environment, which will lead to a plummet of the effectiveness of popular antiseptics and disinfectants. The solution to this problem can be found in the synthesis of new QACs, which exhibit superior antibacterial, antifungal, and antiviral properties.

**Figure 1:**
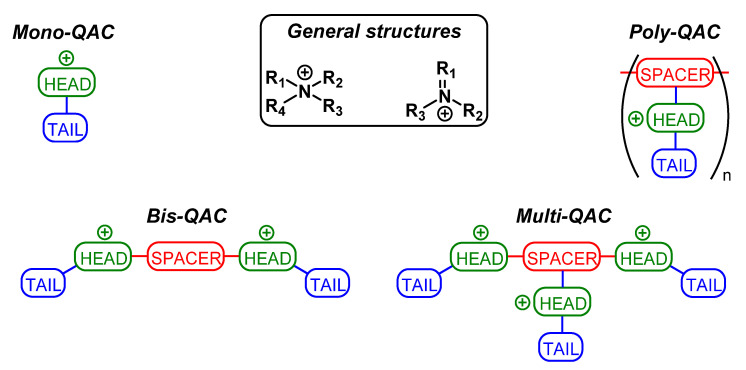
General structures and types of QACs.

The structure of QACs consists of a positively charged nitrogen atom with four or three substituents and one double bond. The core QAC structure can contain one (mono-QAC), two (bis-QAC), or more (multi-QAC, poly-QAC) charged nitrogen atoms, including those in heterocyclic compounds (piperidine, pyridine, imidazole, etc.). One or more of the substituents are usually long aliphatic chains containing at least ten carbon atoms. In the case of bis-QACs, multi-QACs, and poly-QACs, the structure that connects the charged nitrogen atoms (the head or nucleus fragment) is called a spacer or linker, and the alkyl chains extending from the heads (if they are present in the molecule) are called tails (Figure 1). QACs are generally water-soluble and stable. The counterion in these compounds usually does not affect the biological activity but often impacts the solubility of the biocide. The majority of the registered QACs contain chloride or bromide as anions. Due to their amphiphilic nature, QACs are able to form micelles. The critical concentration of micelle formation (CCM) is one of the important characteristics of these substances.

The first studies of QACs as antibacterial agents were carried out at the beginning of the 20th century. Hexamethylenetetramine derivatives exhibited an in vitro bactericidal effect [4,5,6]. With the discovery of benzalkonium chloride (BAC) in 1935 [7], QACs found application in medical practice. Subsequently, the study of this class of compounds has led to the discovery of many valuable properties of QACs, due to which they are now used as surfactants, personal hygiene products, cosmetics, softeners, dyes, biological dyes, and, of course, antiseptics and disinfectants with a wide spectrum of action [8].

**Figure 2:**
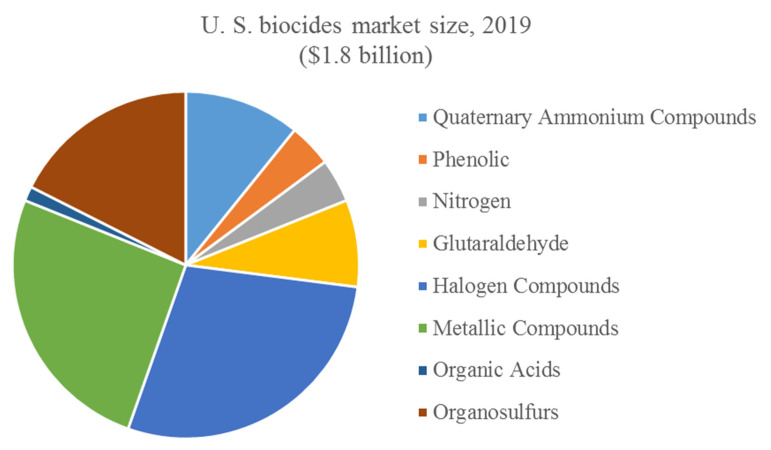
Biocide market in USA.

Therefore, QACs belong to the group of biocides–chemical compounds designed to neutralize, suppress, or prevent the action of harmful organisms by chemical or biological means [9]. As an example, in 2019, QACs accounted for ca. 11% of the whole biocide market in the United States, which equals ca. $192 million (Figure 2) [10].

The U.S. biocide market has grown by ca. 12% since 2016. The global trade of biocides, including QACs, is expected to grow by 3.9% annually and to reach $10.5 billion in 2027, thus evidencing the relevance and popularity of the topic. In other countries, similar trends can be expected due to the unquestionable significance of QACs.

**Figure 3:**
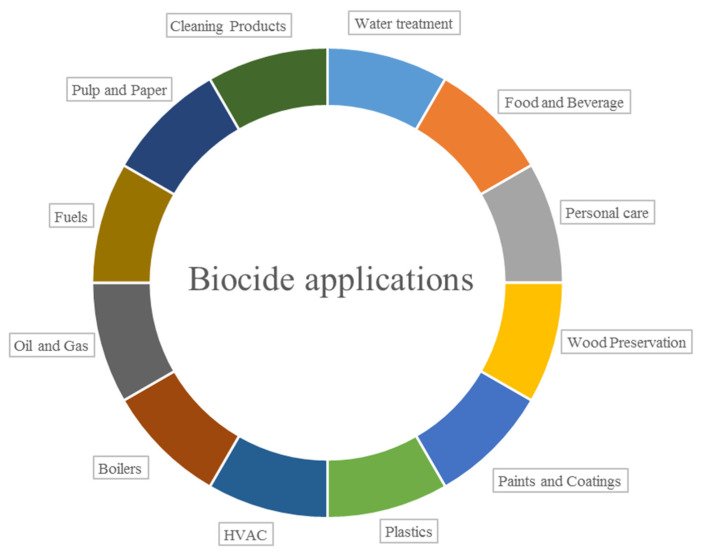
Biocide applications (HVAC—heating, ventilation, and air conditioning).

Biocides are used in a wide variety of fields. Approximately 50% of biocide applications in the global market are in the water purification and paint industry (Figure 3) [10]. However, they also play an important role in the medical field [11].

This review focuses on the main QACs exhibiting the characteristics of biocides, the latest discoveries and issues of this field, and is separated into two parts. The first part presents the main commercial QACs currently used as active substances in antiseptics and disinfectants. The second part describes the scientific research of this class of compounds. Due to the ever-increasing demand for new bactericides and fungicides, the search for compounds active against newly arisen resistant strains of pathogenic bacteria and fungi is one of the most important areas of modern pharmaceutics. Of special concern is the emergence of multidrug-resistant strains (so-called “superbugs”). Therefore, we also discuss the possibilities of applying ionic liquids (ILs) as antimicrobial compounds. ILs, some of which can be classified as QACs, comprise a class of substances with vast molecular diversity. These compounds have been shown to possess a wide range of biological activities, including impressive antimicrobial properties [12,13]. A summary of the bactericidal and fungicidal activities of common ILs, bis-charged ILs, and poly-ILs is provided in the corresponding subsections.

## 2. Antimicrobial Properties of QACs and ILs

### 2.1. Commercial QACs

**Figure 4:**
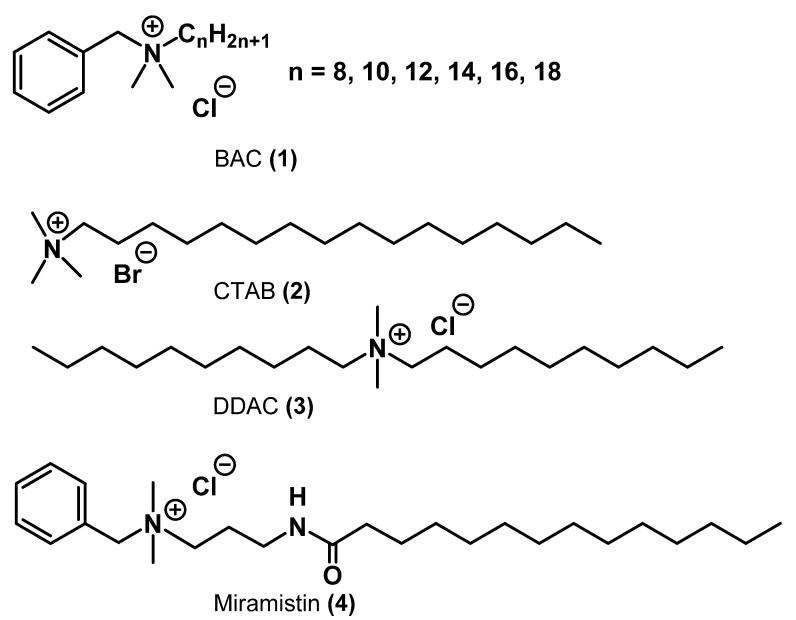
Commercial alkyl QACs.

A significant step in the development of biologically active QACs was the discovery of benzalkonium chloride **1** (BAC) by Domagk in 1935. BAC is a mixture of mono-QACs with benzyl, methyl, and alkyl substituents with different chain lengths from C_8_ to C_18_ (Figure 4). This drug is the first active QAC compound approved by the US Environmental Protection Agency in 1947, and it has been widely used to date [14]. More details about the most important discoveries of that time in the QAC field can be found in the review by Rahn and Van Eseltine [15].

The biological activity of benzalkonium salts depends on the length of the alkyl side chains. It is known that the C_12_-C_14_ compounds exhibit stronger bactericidal effects [16]. Due to its broad antibacterial activity and low toxicity, a mixture of benzalkonium derivatives is used in washing disinfectants for hands and face, mouthwashes, creams, and other cleansing and disinfecting products. BAC exhibits bactericidal activity against *Staphylococcus*, *Streptococcus*, Gram-negative bacteria (*E. coli*, *Pseudomonas aeruginosa*, *Proteus*, *Klebsiella*, etc.), anaerobic bacteria, fungi, and molds. It is also efficient against bacterial strains resistant to antibiotics and chemotherapeutic drugs; it inhibits Staphylococcus plasma coagulase and hyaluronidase. BAC prevents secondary wound infection with hospital strains [17]. In addition, a 0.2% aqueous solution of BAC was shown to inactivate the SARS-CoV-2 virus within 15 s [18].

Further study of this class of compounds led to the discovery of several currently widely known QACs with similar structures: alkyltrimethylammonium bromides. The most famous of them are cetyltrimethylammonium bromide (CTAB) **2** and dialkyldimethylammonium chloride, the main representative of the latter being dimethyldidecylammonium chloride (DDAC) **3**. The addition of the second long aliphatic chain increased the biological activity of the substance against *S. aureus* up to 8 times but, at the same time, increased its toxicity against red blood cells [8].

Miramistin **4** is a nonheterocyclic alkyl QAC and one of the most popular antibacterial agents in antiseptics used in Russia [19]. Miramistin demonstrates a moderate antiseptic effect against pathogenic fungi and viruses. Its aqueous solutions are used in the treatment of pyo-inflammatory diseases in surgery, obstetrics, gynecology, dermatology, urology, dentistry, and ophthalmology [20,21]. Miramistin-containing drugs have a pronounced bactericidal effect on Gram-positive (*Staphylococcus* spp., *Streptococcus* spp., *Streptococcus pneumoniae*, etc.), Gram-negative bacteria (*Pseudomonas aeruginosa*, *Escherichia coli*, *Klebsiella* spp., etc.), aerobic, and anaerobic bacteria, both in the form of monocultures and microbial associations, including hospital strains polyresistant to antibiotics. Moreover, miramistin demonstrates antiviral activities (hepatitis, HIV), prevents wound and burn contamination, and facilitates the recovery of damaged tissues [22].

**Figure 5:**
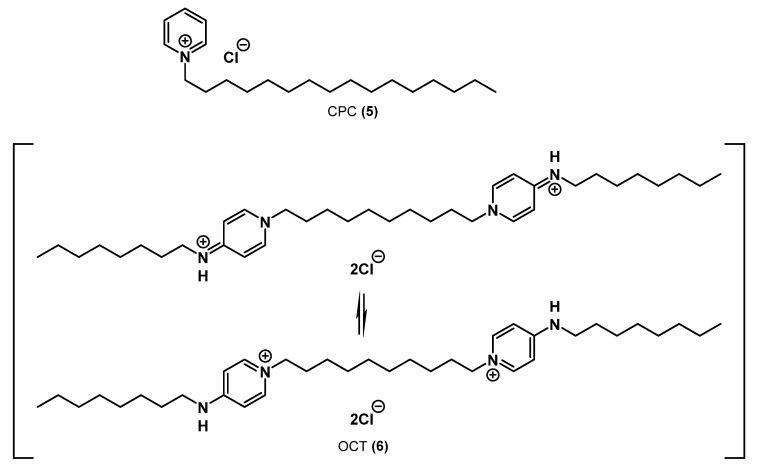
Commercial QACs based on pyridine.

Along with the majority of nonheterocyclic QACs on the antiseptic and disinfectant market, there are also examples of heterocyclic QACs, especially pyridine-based QACs (Figure 5).

The simplest of them is mono-QAC cetylpyridinium chloride **5** (CPC). First described shortly after BAC in 1939 [23], CPC has been extensively used in many mouthwashes and products for oral care [24]. In addition, CPC works as a preservative agent due to its outstanding inhibition properties of bacterial growth.

The second antiseptic of the subgroup is octenidine dihydrochloride **6** (OCT). Its dimeric structure is more complex than that of the other typical substances of this class. Here, two pyridinic nitrogen atoms linked via an alkyl bridge have alkylamine substituents in the para-position. OCT exists in pyridinic and imino forms. Due to its molecular structure, it demonstrates a broad spectrum of antibacterial activity, affecting *S. aureus*, *S. epidermidis*, *P. mirabilis*, *K. pneumoniae*, *E. coli*, *P. aeruginosa*, etc. [25]. Two cation-active centers divided by the long aliphatic carbon chain facilitate molecule binding to negatively charged surfaces of microbial cells. Strong interactions between octenidine and lipids (in particular, cardiolipins) in the bacterial cell membrane have been detected [26]. OCT has an intense residual effect on the skin, which is observed even 24 h after the last application. Due to its antimicrobial properties and skin compatibility, OCT can be used for various local applications where fast action and long-term effects are required, e.g., for disinfecting the skin of patients or treating acute and chronic wounds spontaneously colonized or locally infected by pathogenic bacteria. OCT can also be used for treating surgical equipment, injection sites of central catheters, infected root canals of teeth, candidiasis, acne, and nail infections [26,27,28,29].

**Figure 6:**
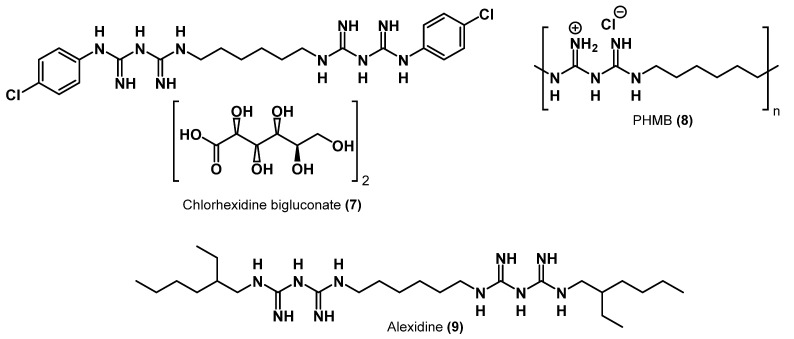
Commercial QACs–biguanide derivatives.

A number of other biocides that play an important role in the modern market of antiseptics and disinfectants should also be mentioned. The antiseptics chlorhexidine bigluconate **7** (CHG), alexidine **9,** and polyhexamethylene biguanide **8** (PHMB) (Figure 6) are guanidine derivatives from the cationic biocide family, as well as the abovementioned QACs [30].

CHG is a symmetrical bis-biguanide connected by an alkyl chain; it carries two positive charges at physiological pH. Developed in the early 1950s during the screening for antimalarial drugs, CHG has since recommended itself as a broad-spectrum antibacterial drug. CHG is one of the first antiseptics used on the skin and for decontamination of wounds. It is typically applied in the form of bigluconate, gluconate, dichloride, and acetate salts. Antiseptic drugs, which contain chlorhexidine bigluconate as an active substance, have a fairly wide spectrum of action. They are active against Gram-positive bacteria but not Gram-negative bacteria and mycobacteria or fungi. CHG is widely used in surgery and hand washing in the treatment of wound sepsis. It is also used in various oral hygiene products, as an anti-plaque agent, and in periodontal treatments. Similar activities were exhibited by aleksidine (Figure 6) [31,32,33,34].

PHMB is an alkyl biguanide polymer that can be used in a soluble form as chloride. It is an effective alternative to traditional antiseptics due to its low toxicity and superior antibacterial and antifungal activity [35]. It is used for treating swimming pools and fabrics, in cleaning products, and as a disinfectant for contact lenses and mouthwashes [36].

### 2.2. The Latest Scientific Discoveries in the QAC Field

**Figure 7:**
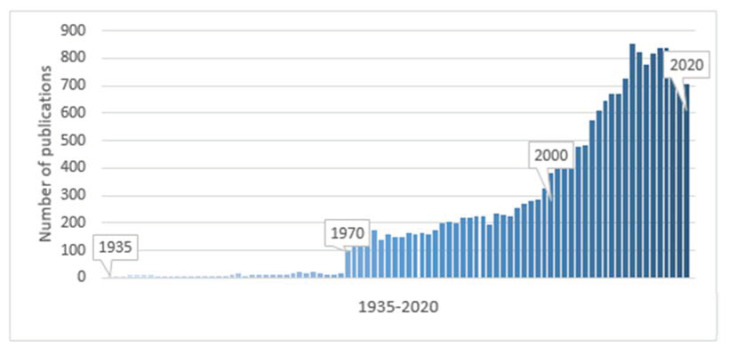
Number of publications involving QACs from 1935 to 2020 (SciFinder, January 2021).

The simplicity of synthesis, vast structural diversity, and high biological activity drive numerous scientific studies on QACs. Over the past 85 years, after the emergence of the class of cationic biocides, the number of publications on the topic has been arising significantly (Figure 7). According to SciFinder, more than 700 articles on QAC properties were published in 2020.

The scientific society proposes various synthetic procedures and applications for QACs, analyzes their structural fragments, and establishes the relations between the efficiency and molecular structure [37,38]. The last approach, known since the 19th century [39], is widely used in quantitative studies on various activities of chemical substances (QSAR, quantitative structure–activity relationship) [40].

Judging from the basic structure (Figure 1), one can change several parts in a given QAC to determine their impact on its activity:

Head. The number of charged nitrogen atoms (mono-, bis-, multi-QAC), as well as the head structure (non-heterocyclic, heterocyclic, aromatic), can be changed.

Spacer. The structure (aliphatic, aromatic, saturated, unsaturated, mixed, etc.) can be changed.

Tail. The structure (saturated, unsaturated, branched, unbranched) and the length of the aliphatic chain can be changed.

Substituents. A desired group can be introduced into any of the abovementioned fragments of the QAC molecule.

Hereafter, we will focus on representative examples of synthetic biocidal QACs obtained by various scientific groups in recent years. The effect of the structural fragments of the biocides on their biological activity will also be considered. The material is presented sequentially, depending on the QAC charge (mono-QAC, bis-QAC, poly-QAC). Additional information on studies on antimicrobial activity, surfactant properties, usage, and synthesis can be found in recent reviews on the topic [8,41,42,43,44,45,46,47,48,49,50,51].

#### 2.2.1. Single-Charged QACs (Mono-QACs)

**Figure 8:**
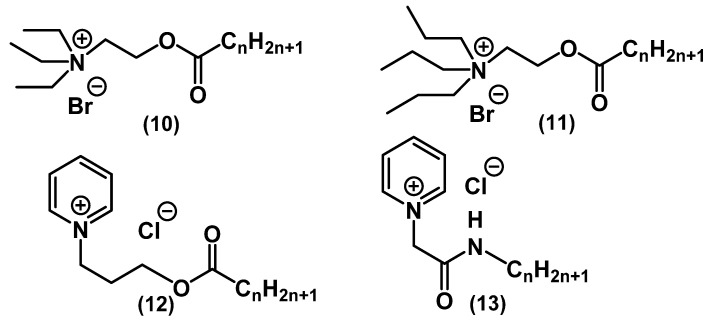
“Soft” mono-QACs.

Thorsteinsson and colleagues developed “softer” analogues of the existing QAC biocides [52]. While “hard drugs” (CPC, BAC) are specified as drugs that are not subject to in vivo changes, “soft drugs” are metabolized to nontoxic compounds (Figure 8) [43].

Due to the introduction of amide and ether groups, the synthesized QAC molecules **10**-**13** are deactivated and decomposed into amides, fatty acids, and alcohols. Compounds without alkyl chains or with short chains (C_2_, C_3_) were found to be inactive. Substances with C_12_–C_18_ alkyl tails exhibited antibacterial activity comparable to a known analog (BAC 1) against *E. coli*, *S. aureus,* and *P. aeruginosa*. Additionally, some compounds from series **11** showed activity against herpes simplex virus (HSV-1).

**Figure 9:**
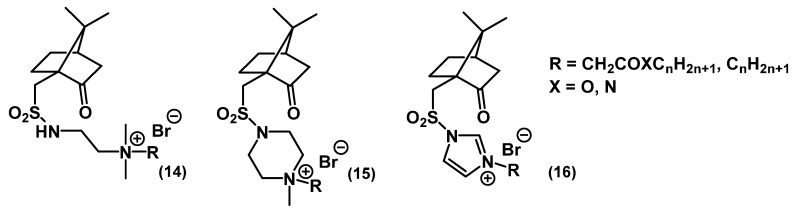
CSA-based mono-QACs.

Miklas and colleagues carried out the synthesis and studied the biological properties of QACs based on camphorsulfonic acid (CSA) **14**-**16** (Figure 9) [53,54].

Upon changing the QAC core from ammonium to a less saturated heterocyclic structure (imidazole), the antimicrobial activity of the compounds gradually decreased. Salts with alkyl tails exhibited better activity than their ester and amide counterparts. The optimal chain length was found to be C_12_-C_14_.

**Figure 10:**
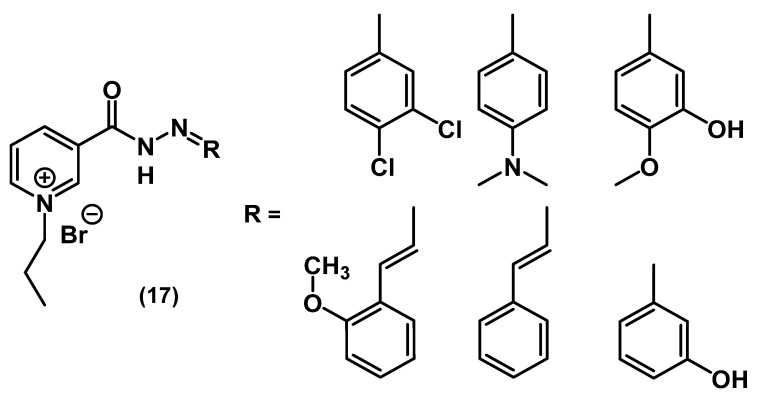
Mono-QACs containing hydrazide bridges.

In a recent work, Ali and colleagues developed new pyridine-based QACs from Schiff bases of nicotine hydrazines (Figure 10) [55].

These substances had good water solubility, most likely due to the presence of hydrazide groups. Despite the shorter alkyl chains (compared to typical QACs), a series of substances **17** showed high activity against colonies and biofilms of *E. coli* and *S. aureus*. According to this study, the presence of donor groups in the phenyl ring of the R substituent increased the bactericidal activity.

**Figure 11:**
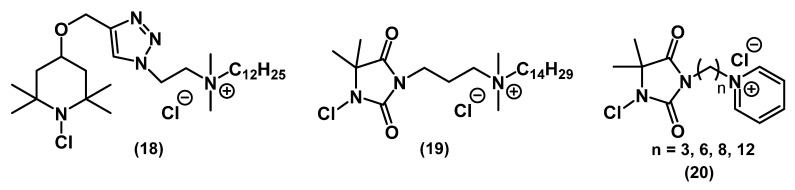
Mono-QACs containing *N*-chloramines.

In the works of Liu and colleagues, the effect of combining two biocidal fragments (*N*-chloramines and alkyl QACs) in one molecule **18**-**19** on bactericidal properties was studied (Figure 11) [56,57,58].

Chloramines act on bacterial cells through the oxidative transfer of chlorine to biological receptors which leads to cell lysis. The attachment of the QAC molecule with a positive charge allowed anchoring of the *N*-chloramine moiety on the surface of the bacterial cell, thus enhancing the effect [56]. The introduction of a long alkyl chain into the compound leads to the rupture of the bacterial membrane, penetration of the biocide into the cell, and a subsequent enhancement of the bactericidal effect [57,58]. At the same time, Li and colleagues combined a pyridinic QAC with *N*-chloramine **20** (Figure 11). The antibacterial activity of this compound was similar to that presented by Liu [59].

**Figure 12:**
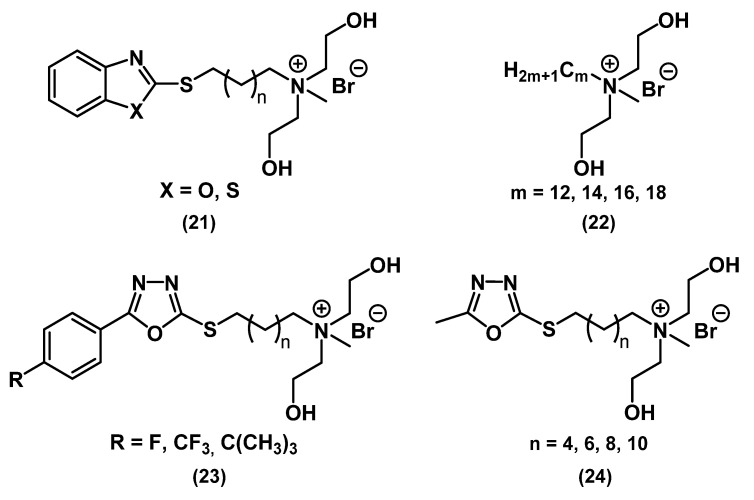
Mono-QACs containing hydroxyl groups.

In the works of Wang and Hou, a similar approach to changing the structure of QAC by adding biologically active fragments to the molecule was used (Figure 12) [60,61].

Initially, guided by the hypothesis that hydroxy groups should stimulate membrane penetration and cell destruction, a series of hydroxy-QACs **22** with different alkyl chain lengths was synthesized. All the resulting compounds exhibited lower antibacterial activity than CHG; they also demonstrated antifungal activity with an optimal tail length of C_12_. It should be noted that the toxicity of the compounds correlated with their activity [60]. Then, a fragment of oxadiazole derivatives **23**-**24**, benzothiazole (X=S) **21,** and benzoxazole (X=O) **21** was introduced into the QAC molecule, which led to an increase in bactericidal and fungicidal activity and a decrease in toxicity in epithelial cells and erythrocytes [61].

**Figure 13:**
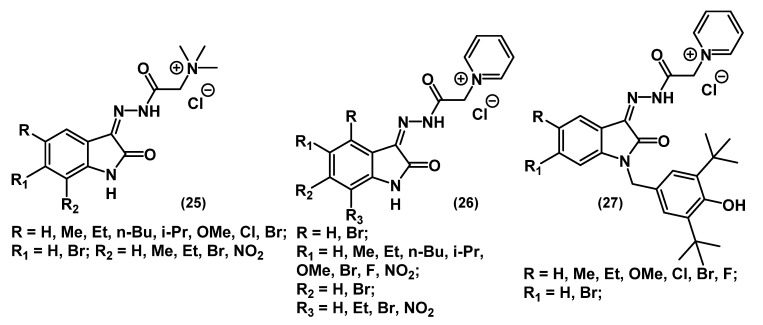
Isatin-based mono-QACs.

Bogdanov and colleagues explored the microbiological effect of isatin-based QACs (Figure 13) [62].

As seen from the figure, the structures of these ammonium **25** and pyridine **26**-**27** salts contain no long alkyl chains. Therefore, the cytotoxicity of these compounds is significantly lower than that of typical QACs. However, the antibacterial activity is markedly reduced in the absence of quaternary nitrogen tails. Thus, none of the compounds from this series showed a biocidal effect against the Gram-negative bacteria *E. coli* and *P. aeruginosa*. On the other hand, these salts inhibited the growth of Gram-positive bacteria (*S. aureus* and *B. cereus*) and fungi (*C. albicans*) at concentrations comparable to modern antibiotics (chloramphenicol and norfloxacin). Overall, QACs with pyridinium nuclei and donor substituents in the aromatic part of isatin **27** turned out to be more active than the others.

**Figure 14:**
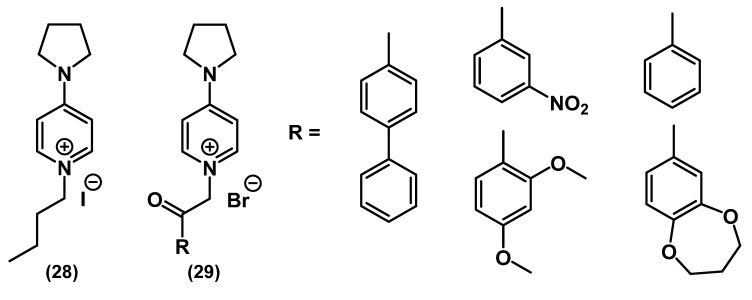
Mono-QACs containing aryl substituents.

Rusew and colleagues presented a work, in which long lipophilic tails in QACs were replaced by more compact aryl-containing substituents (Figure 14) [63].

The results of a broad antibacterial screening appeared to be nontypical for cationic biocides. Compounds with biphenyl and 1,3-dimethoxyphenyl **29** substituents selectively inhibited the growth of *E. coli* (Gram-negative) and *S. aureus* (Gram-positive) but no other Gram-positive and Gram-negative bacteria. In a quantitative sense, the inhibiting zones of these substances were similar to kanamycin.

**Figure 15:**
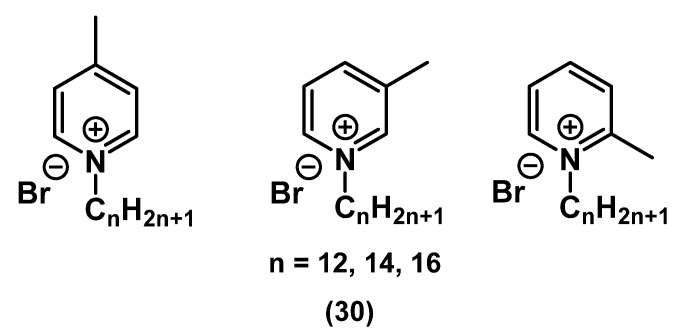
Picolinic mono-QACs.

Kuca and Soukup studied the biological activity of picolinic QAC with methyl substituents **30** (Figure 15) [64].

It was found that the position of the substituent did not significantly affect the biocidal effect of methylpicolinates, possibly due to the small size of the methyl substituent. Overall, picolinates showed a comparable or even superior bacteriostatic effect compared to BAC on a wide range of pathogens. The optimal tail length was C_14_-C_16_, and higher activity was observed in Gram-positive bacteria than in Gram-negative bacteria, as with most QACs.

**Figure 16:**
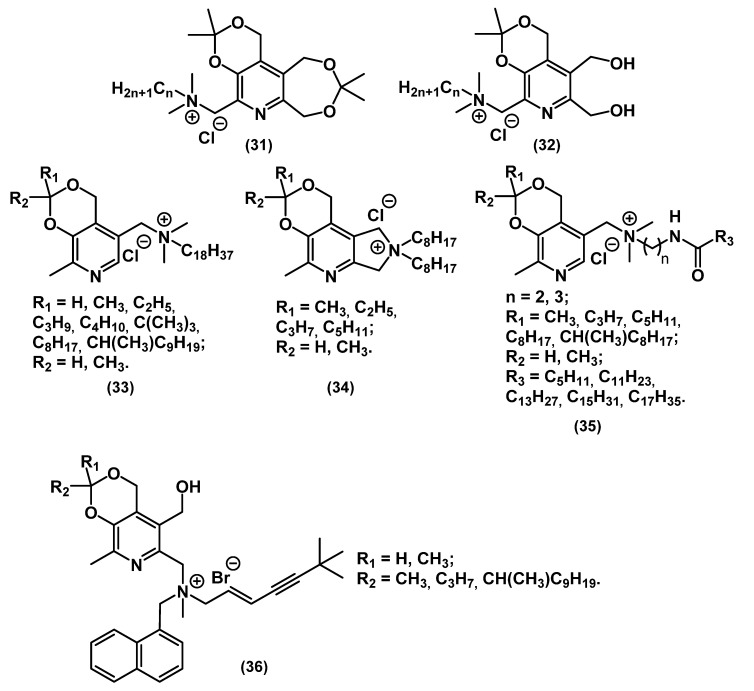
Pyridoxin-based mono-QACs.

Shtyrlin and his colleagues created a pyridoxine-based QAC library, including bis-derivatives, which will be discussed in the corresponding part of the review (Figure 16) [65,66,67,68,69,70].

Pyridoxin functional derivatives **31**-**36** exhibited a broad spectrum of antibacterial and antifungal activity; at that time, they were more active against Gram-positive bacteria than Gram-negative bacteria. It should be mentioned that a combination of the antifungal drug terbinafine with pyridoxin-based QAC **36** was efficient against mixed colonies of pathogenic bacteria and fungi. This example proved the advantage of combining two different biocide fragments in one molecule.

**Figure 17:**
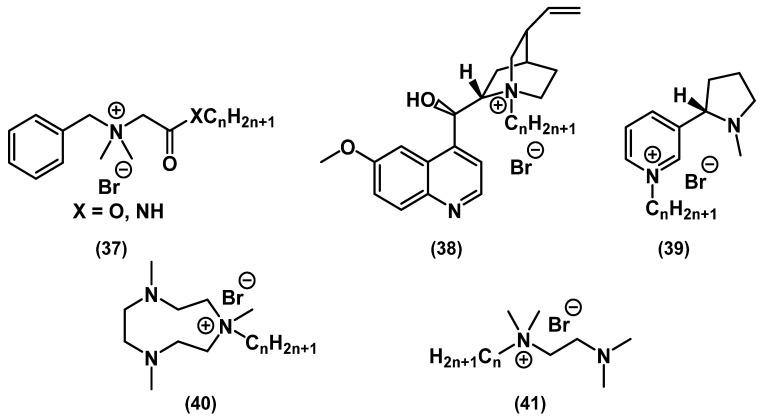
Mono-QACs from Wuest’s and Minbiole’s works.

A significant contribution to the development of QACs as a class of cationic biocides was made by the groups of Wuest and Minbiole (Figure 17) [71,72,73,74,75,76].

It was found that close structural analogs of BAC **37** containing amide and ester groups exhibited comparable activity and lower toxicity than BAC [76]. QAC derivatives of natural compounds (quinine **38** and nicotine **39**) demonstrated a wide spectrum of antibacterial action, thus justifying the search for other platforms of natural origin to expand the library of active QAC compounds [74].

An overview of the antibacterial activity of mono-QACs, analyzed in the review, is shown in Table 1.

#### 2.2.2. Common Ionic Liquids and Ionic Liquids with Active Pharmaceutical Ingredients (API-ILs)

**Figure 18:**
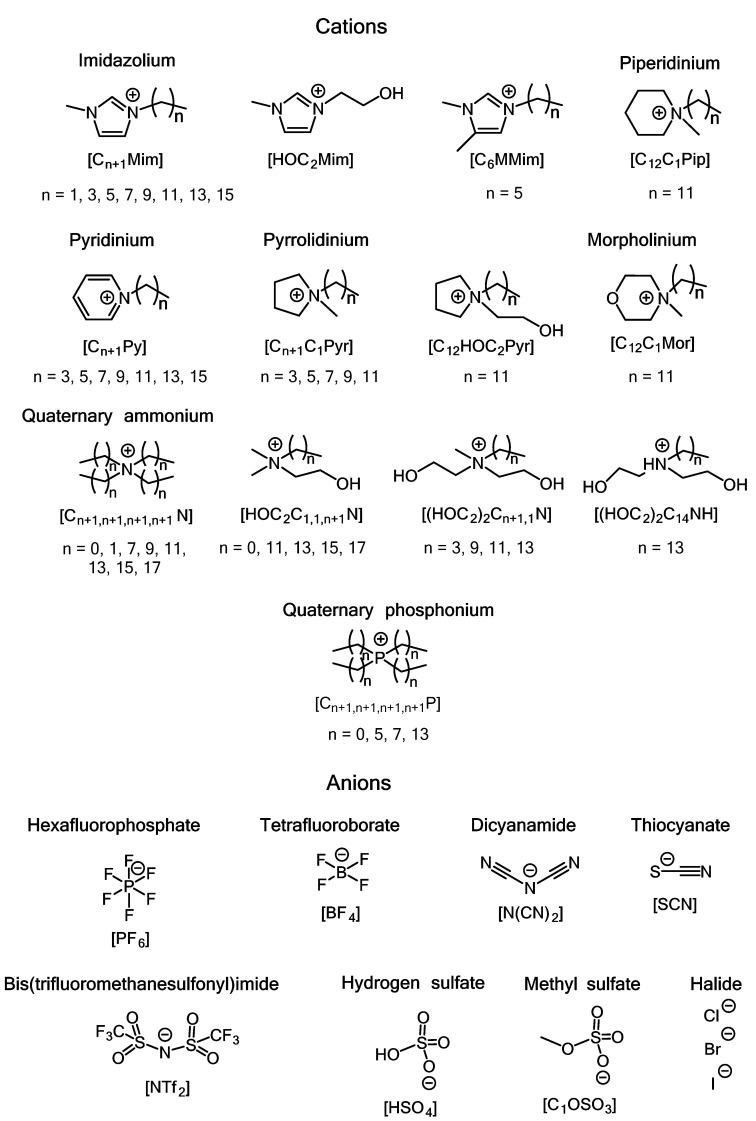
Cations and anions commonly used in ILs with known antimicrobial activity.

ILs are organic salts that generally exist in liquid form at a wide range of temperatures. The most common ILs are composed of a bulky organic cation and a more compact anion (Figure 18). Due to its broad applications in chemistry, this class of compounds has been studied thoroughly, and the chemical and physicochemical properties, as well as biodegradation potential, of various ILs have been determined [12,77].

Initially, ILs were considered green solvents that could replace traditional toxic organic solvents in various chemical processes [78]. However, when evidence of the high biological activity of various classes of ILs has emerged, these substances have quickly become candidates for new drugs and drug-like molecules. In particular, the antimicrobial activity of ILs has attracted much attention, and their possible medical and environmental applications have been proposed [12,13,79,80].

A subclass of ILs with quaternary ammonium cations (which includes several of the above-discussed QACs) has promptly been established as a promising alternative to traditional antimicrobial substances [80]. ILs with other cations have also demonstrated prominent bactericidal and fungicidal activities [12,79]. Some of these ILs (e.g., *N*-hexadecylpyridinium chloride, or cetylpyridinium chloride, CPC, which is also classified as a QAC) have been extensively used as antiseptics for a long time [81,82]. The first successful results of studies on the antimicrobial activities of various ILs have led to the rapid development of API-ILs (active pharmaceutical ingredient–ionic liquid), that is, known commercial drugs in an ionic liquid form [12,83,84].

An overview of the antimicrobial activities of various members of common IL classes is provided in Table 2 and Table S1. In most cases, there is a direct relation between the length of the alkyl side chain in the cation and the IL antimicrobial activity. ILs with relatively short side chains (ethyl, butyl, hexyl) usually demonstrate weak activity (see Table S1), whereas those with long side chains (dodecyl, tetradecyl, hexadecyl) can be strong inhibitors of some bacterial and fungal species, including biofilm-forming and drug-resistant species (see, e.g., entries for [C_n_Mim][A], n = 12–16, and [C_n_Py], n = 12–16, in Table 2) [81,85,86,87,88,89]. For instance, 1-dodecyl-3-methylimidazolium bromide ([C_12_Mim][Br]), *N*-dodecyl-*N*-methylpyrrolidinium bromide ([C_12_C_1_Pyr][Br]), and *N*-dodecyl-*N*-methylpiperidinium bromide ([C_12_C_1_Pip][Br]) demonstrated both high antimicrobial and low hemolytic activity, thus allowing their successful application in medicinal practice [90,91]. Cholinium-based ILs with long alkyl chains, in particular, *N*-(2-hydroxyethyl)-*N*,*N*-dimethyl-*N*-tetradecylammonium bromide, *N*-(2-hydroxyethyl)-*N*,*N*-dimethyl-*N*-hexadecylammonium bromide, and *N*-(2-hydroxyethyl)-*N*,*N*-dimethyl-*N*-octadecylammonium bromide, efficiently inhibited the growth of various bacterial strains, including antibiotic-resistant strains (see entries for [HOC_2_C_1,1,n_N][Br], n = 14–18, in Table 2) [92]. Surface-active cholinium ILs with the dodecylbenzenesulfonate anion demonstrated significant activity against Gram-negative and Gram-positive bacteria, fungi, and single-cell algae; these ILs were proposed to be used as coatings for the prevention of biofilm formation on stone surfaces [93].

It should be noted that the anion can also have a significant impact on the antimicrobial activity. Thus, the antibacterial activity of 1-butyl-3-methylimidazolium ILs with different anions against pathogenic and semipathogenic Gram-negative and Gram-positive bacteria varied significantly depending on the anionic nature [94]. In particular, 1-butyl-3-methylimidazolium bis(trifluoromethanesulfonyl)imide ([C_4_Mim][NTf_2_]) demonstrated the highest activity against *E. coli* (see entries for [C_4_Mim][A] in Table 2 and Table S1); however, its anti-adhesive activity was significantly lower than that of several other ILs tested. A different picture was observed in the case of 1-hexyl-3-methylimidazolium IL, among which 1-hexyl-3-methylimidazolium nitrate ([C_6_Mim][NO_3_]) demonstrated the highest activity against *E. coli* and several other microorganisms tested (see entries for [C_6_Mim][A] in Table S1) [95]. Interestingly, it was demonstrated that for ILs with tris(pentafluoroethyl)trifluorophosphate anions, the antimicrobial activity decreased upon increasing the alkyl side chain length [96].

**Figure 19:**
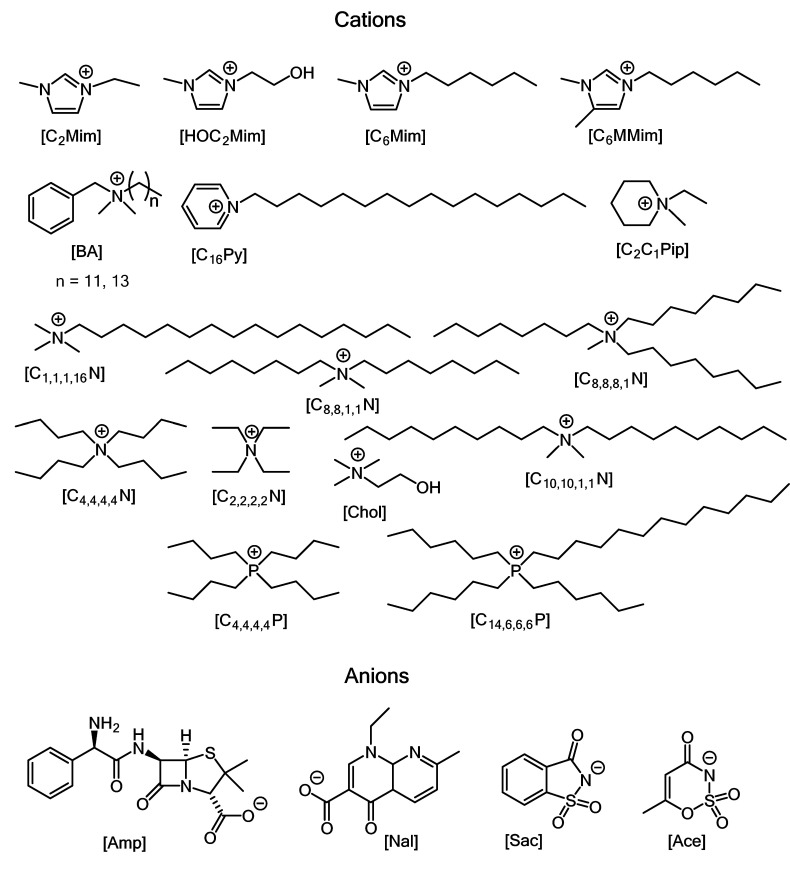
Cations and anions used in antimicrobial API-ILs.

Of special interest are ILs containing antimicrobial moieties in their anions or cations. The API-IL concept allows simultaneously solving two common issues of traditional drugs: low solubility in aqueous media and tendency to form polymorphs [12]. Examples of bactericidal API-ILs are given in Figure 19, Table 3, and Table S2. Thus, API-ILs bearing ampicillin as their anion in combination with cetylpyridinium or 1-hexadecyl-2,3-dimethylimidazolium as their cation demonstrated improved activity against several Gram-negative and Gram-positive bacterial strains, including ampicillin-resistant E. coli strains, compared to the ampicillin sodium salt (see the corresponding entries in Table 3) [82,97].

#### 2.2.3. Double-Charged QACs (Bis-QACs)

Bis-QAC (or so-called “twin surfactants”) is a subclass of synthetic amphiphiles that contain two cationic nitrogen atoms, a spacer linking them, and two lipophilic alkyl substituents [100]. These are common characteristics of typical bis-QAC, the exact structure of which can vary greatly. The intense development of bis-QACs began later than that of mono-QACs in the 1980s with the discovery of octenidine (see the Commercial QACs section). Nonetheless, there are many publications on the synthesis and biocide properties of bis-QACs.

**Figure 20:**
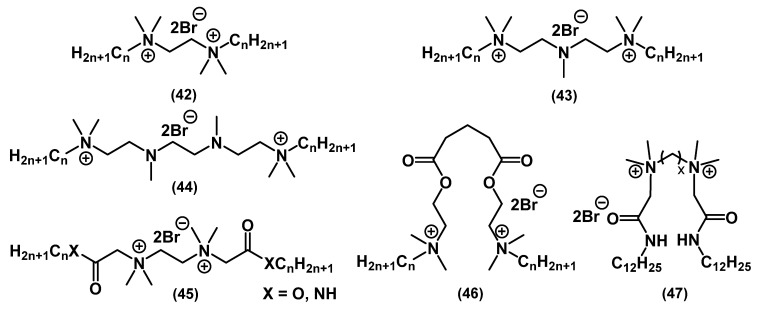
Alkyl bis-QACs.

A significant number of alkyl bis-QACs were synthesized to test the effect of the total charge of the molecule on the activity (Figure 20).

Bis-QACs with ester spacer **46** showed better activity than their mono analogues, both against Gram-positive and Gram-negative bacteria and fungi [101]. It is worth noting that the activity against *E. coli* was nonlinear and plummeted upon increasing the alkyl chain length from C_12_ to C_14_. This relationship, which is known for the biocidal action of amphiphils on Gram-negative bacteria, is called the “cut-off” effect. It was described by Devinsky and colleagues as a consequence of membrane penetration [102]. The addition of a second charged nitrogen atom increased the activity 3-fold in *S. aureus* and 4-fold in *E. coli* in the work of Hodye (substance **47**). The activity also correlated with the distance between the heads, with the optimal spacer length being C_6_ [103]. Wuest and Minbiole and colleagues studied the biocidal action of QACs based on polyamines **43**-**44** [71,104]. Tetramethylethylenediamine derivatives (TMEDAs) **42** turned out to be an extremely promising class of biocides because of their simple synthesis, cheap starting materials, and high activity [75]. In all the above-mentioned studies, the biological effect on pathogenic bacteria increased 3–4 times, especially for Gram-negative strains, compared to mono-QACs.

**Figure 21:**
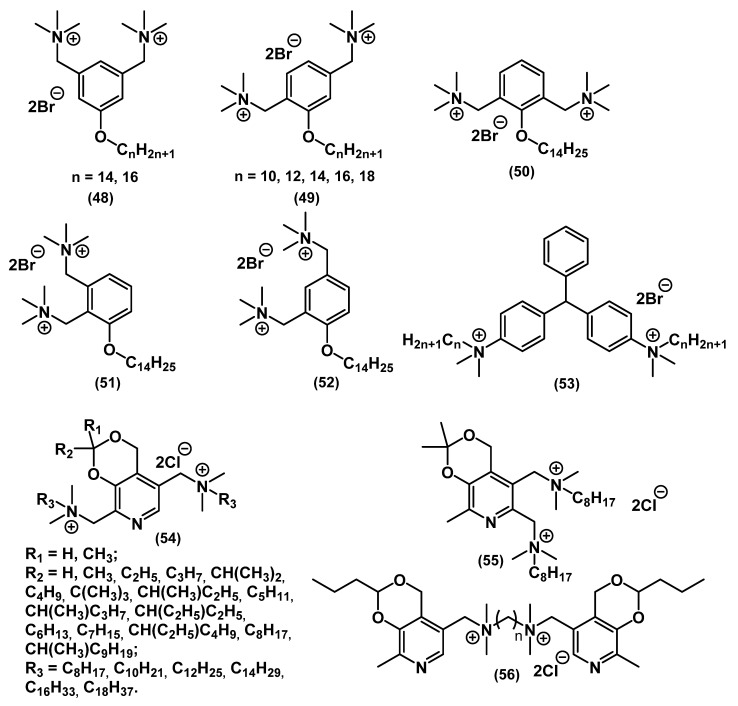
Alkyl bis-QACs containing aromatic spacers.

Changing the spacer in the bis-QAC structure is one of the key factors in the design of target molecules. Thus, the aforementioned alkyl bis-QACs can contain aromatic spacers (Figure 21).

A study by LaDow and colleagues showed that bis-QACs **48**-**52** inhibited the growth of Gram-positive bacteria at approximately the same concentration as their mono analogs. However, bis-QACs had a much stronger effect on Gram-negative bacteria, which was confirmed by other studies [105]. In continuation of their work on the study of pyridoxine QAC derivatives, Shtyrlun and colleagues noted a clear dependence of the activity of compounds **54** on their lipophilicity. Thus, the values of the lipophilicity coefficient for the most active compounds (C_10_, C_12_) were in the range of 1 to 3; at values higher than 6 or lower than 0, the activity decreased sharply [106]. Forman and colleagues studied QAC derivatives of malachite green **53**, comparing its mono- and bis-QACs. Analogs with two long alkyl chains were generally comparable to mono-QACs but were more efficient against resistant bacteria [107].

**Figure 22:**
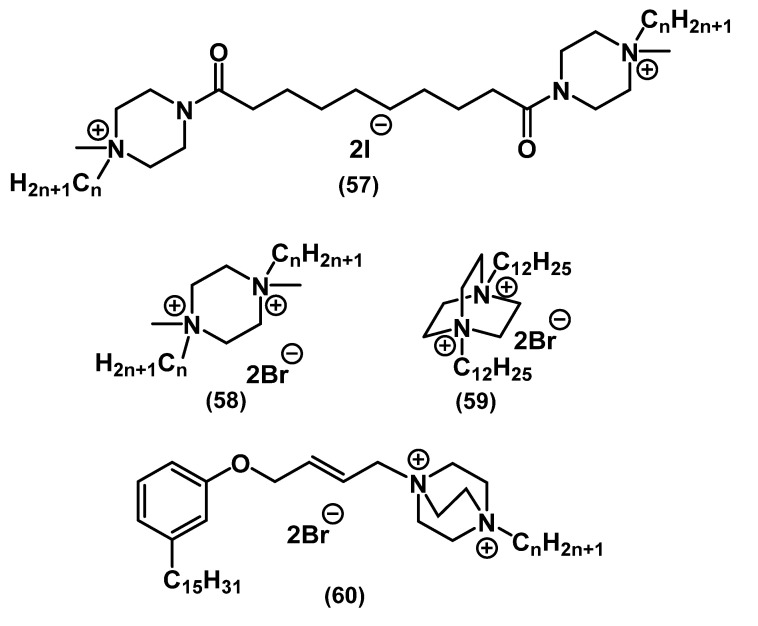
Bis-QACs containing saturated heterocycles.

Similar to mono-QACs, the head of bis-QACs can have a saturated heterocyclic structure (Figure 22).

Kourai and colleagues, in their study of bis-QAC derivatives of piperazine **57**, found that compounds with different spacer structures but the same lipophilicity exhibited different activities. This fact suggested that the dependence of the biocidal action on lipophilicity was valid only for the series of QACs differing in the length of the tail [108]. Kontos and colleagues tested the dependence of the activity of **58**-**59** on the rigidity of the structure. The initial assumption that a more flexible structure would provide easier passage through the bacterial membrane and accelerate cell lysis turned out to be erroneous. Thus, derivatives of the more rigid amine structure **59** of diazobicyclooctane (DABCO) were most active in the series [109]. A series of heterocyclic QACs based on cardanol **60** was developed by Ma and colleagues [110]. Along with moderate antibacterial activity, the compounds appeared to be good surfactants.

**Figure 23:**
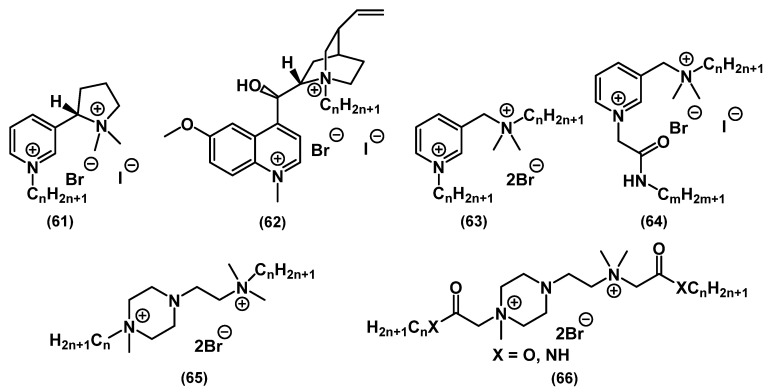
Mixed bis-QACs.

There are several examples of mixed bis-QACs carrying two different heterocycles or heterocyclic and alkyl parts (Figure 23).

In the continuation of the work on preparation of the above-mentioned QAC derivatives of quinine and nicotine, the usual “activation” of the second nitrogen charged center did not lead to a significant increase in the activity of **61**-**62**. Presumably, the total charge of the molecule does not affect the activity as strongly as the addition of the second alkyl chain [74]. In the work of Schallenhammer and colleagues, hybrid bis-QACs **63**-**64** combining CPC **5** and BAC **1** showed higher activity against Gram-negative bacteria than each of the commercial “source drugs” applied separately. At the same time, hybrid monoderivatives did not show such a result [111]. Piperazine bis-QAC derivatives **65** and their “soft” analogs **66** showed similar relationships with the previous bis-QACs [72,112].

**Figure 24:**
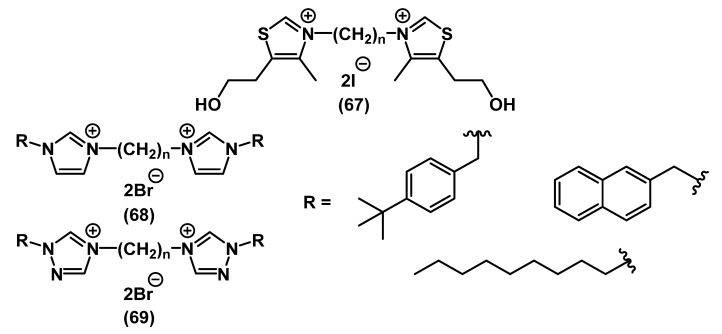
Bis-QACs containing saturated heterocycles.

Additionally, there is a range of interesting works concerning QACs with polynuclear heterocycles with several heteroatoms (Figure 24).

Thomas and colleagues synthesized QACs based on bis-thiazole **67**, bis-imidazole **68** and bis-triazole **69**. While thiazole derivatives with an alkyl spacer and without lipophilic tails **67** did not show high activity, bis-QACs with nitrogen heterocycles **68**-**69** demonstrated MIC values lower than that of CHG [113].

**Figure 25:**
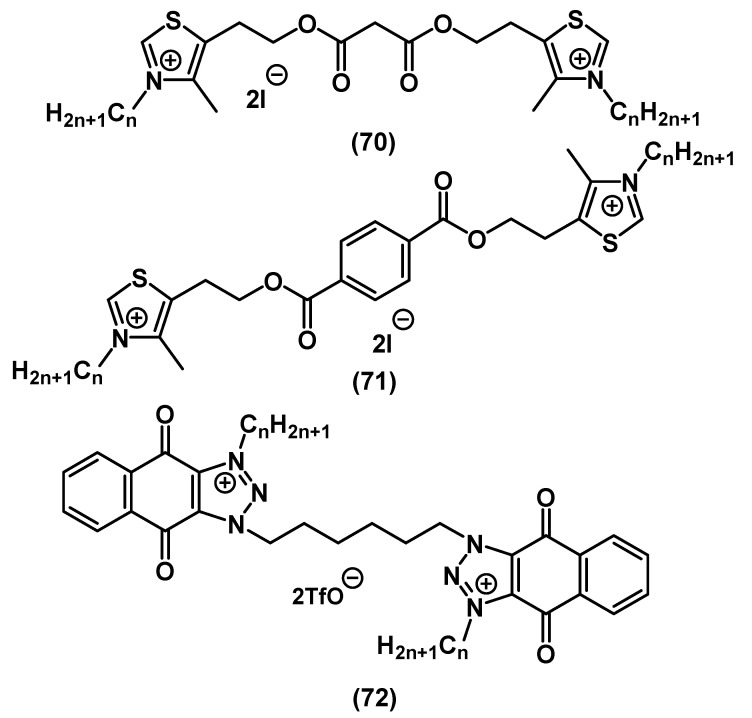
Bis-QACs containing unsaturated heterocycles.

In contrast, in the work of Shirai and colleagues, thiazole bis-QACs with alkyl tails **71** (Figure 25) exhibited a wide spectrum of antibacterial and antifungal effects [114]. This is additional evidence that the tails in the QAC structure are strong inducer of the biological effect against pathogens. Shrestha and colleagues studied the antibacterial and antifungal activity of bis-triazole QAC based on benzoquinone **72** (Figure 25) [115].

**Figure 26:**
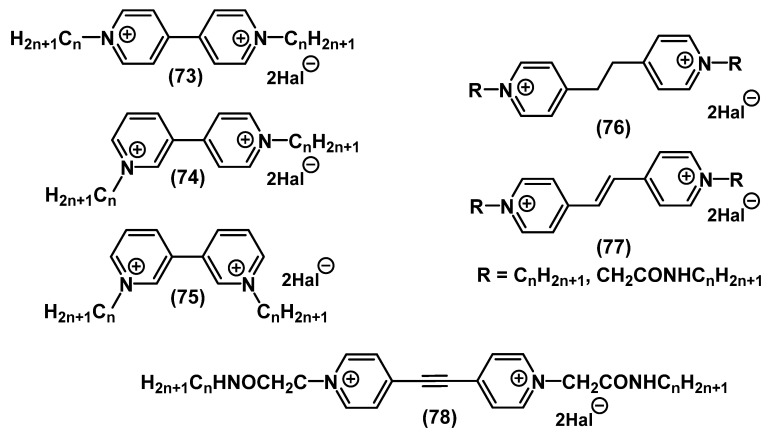
Pyridine-based bis-QACs without spacers and with alkyl spacers.

Inspired by the success of octenidine on the market of cationic biocides, scientists have begun to actively develop a class of bispyridinium salts with various types of spacers (Figure 26).

In the work of Minbiole and colleagues, bispiridinium QAC derivatives of paraquats **73**-**75** and bis-QACs without a spacer between pyridinium heads were studied. The activity of meta-**75** and parameta-analogs **74** was more pronounced. Cyclovoltamperometric analysis showed the predisposition of paraquats **73** to reversible oxidation-reduction processes and the formation of “superoxide”. This presumably increases the toxicity, while metaquats **75** and parametaquats **74** are not subject to this possibility and thus can be less toxic. In addition, given the high activity of parameta-derivatives **74**, this indicates the incoherence between the increase in the biocidal action of QACs and their redox capacity [116,117]. A study on the dependence of the activity on the rigidity of the structure for bispyridinium-QACs with alkyl spacers with different saturations **76**-**78** showed ambiguous results. While this dependence was not observed for QACs with alkyl chains as tails, and the MIC values remained approximately at the same level, in the case of bis-QACs with amide bridges in the tails, a sharp decrease in the activity was observed upon increasing the structural rigidity. The authors showed that in such rigid structures, the bis-QAC activity decreased as the charged heads moved away from each other [118].

**Figure 27:**
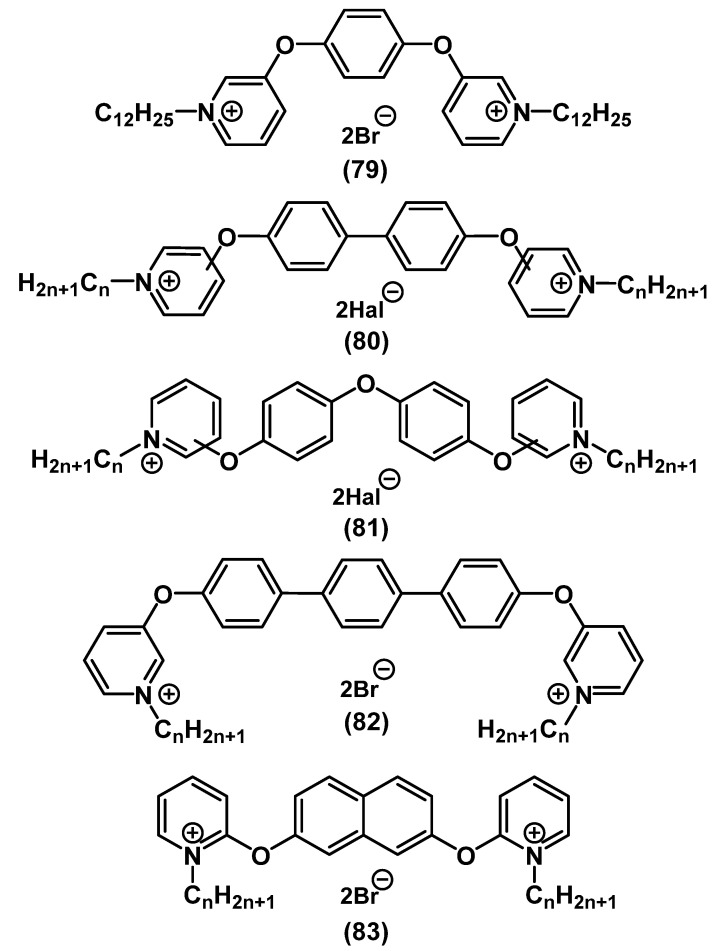
Pyridine-based bis-QACs containing aromatic spacers.

In the last few years, new biocidal pyridine-based bis-QACs containing an aromatic fragment in a spacer have been synthesized (Figure 27). Thus, bis-QACs with 1,4-dioxophenyl as spacer **79** were significantly more active than commercial QACs (BAC 1, CHG 7) [119,120,121]. Vereshchagin’s group studied the dependence of the activity of biocides on the size of the aromatic spacer of salts, as well as the location of the spacer relative to the charged pyridinium nitrogen **79**-**83** [122,123,124,125,126]. It was discovered that the QAC activity increased upon increasing the length of the aromatic spacer. The activity increased in the following order: mono- **79** < bi- **80** < terphenyl **82** [122,124]. It can be assumed that in such structures, the activity increases with an increase in the distance between the nitrogen atoms. It is worth noting that the optimal length of the alkyl tails also varied in this series: C_12_ for phenyl **79**, C_10_ for biphenyl **80**, and C_8_ for terphenyl **82**. The influence of the position of substitution in pyridine turned out to be ambiguous. In the case of biphenyl **80**, the meta-salts turned out to be slightly more active than the para-derivatives, while the opposite was observed for the more mobile biphenyl ether **81** [123,126]. The ortho-salts showed strikingly lower activity. However, this was not the case for QACs of 2,7-dihydroxynaphthalene derivatives **83**, and the biocidal effect of the orthosalts was extremely high [125]. From the viewpoint of their activity, the leading compounds from the series of bis-QACs with aromatic spacers were superior to the widely used QACs, such as CHG **7**, CPC **5**, BAC **1**, and miramistin **4**, and were comparable to OCT **6** (Figure 27).

**Figure 28:**
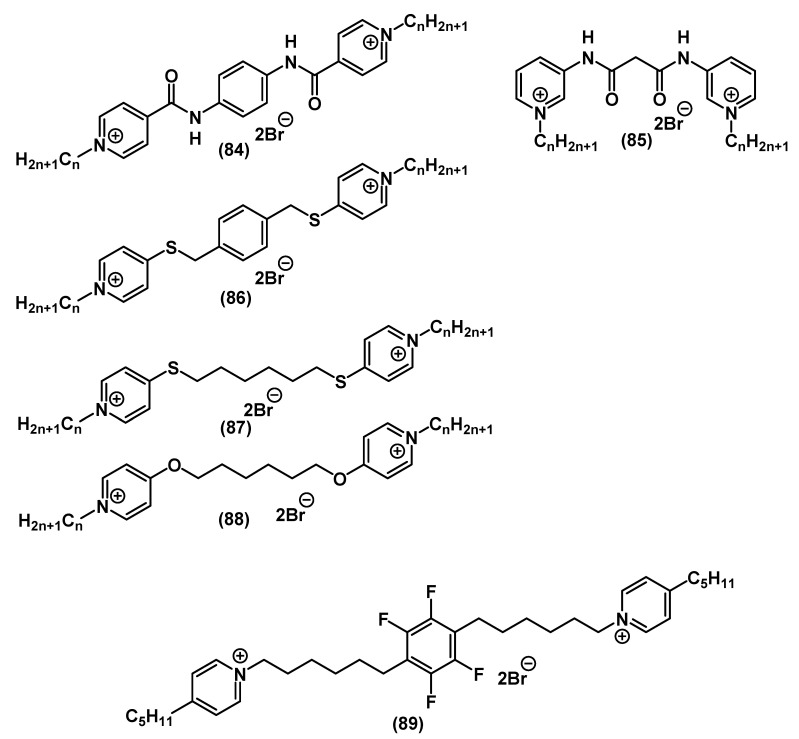
Pyridine-based bis-QACs containing mixed spacers.

There is a broad variety of structures of bispyridinium salts containing mixed spacers (Figure 28).

Kourai and colleagues initiated studies on bis-pyridine salts **84**, **86**-**88** [127,128,129,130,131,132]. Later, Obando and colleagues proposed the synthesis of biologically active bis-QACs containing mixed alkyl-aromatic spacers **89** [133]. In their recent investigation, Hao and colleagues performed a comprehensive physical-chemical and biological analysis of bis-QACs with amide bridges **85** [134].

**Figure 29:**
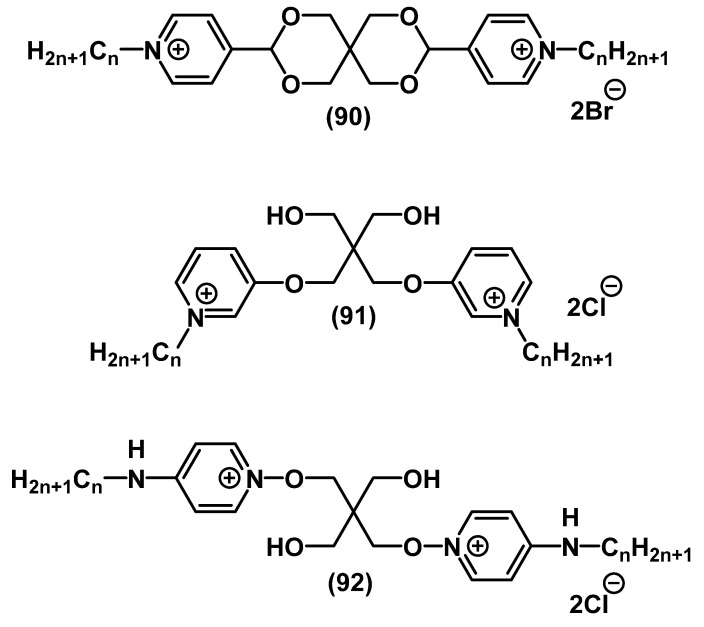
Pyridine-based bis-QACs containing pentaerythritol.

Pentaerythritol-based bis-QACs **90**-**91** (Figure 29) were developed by Yamamoto and colleagues. These substances revealed a broad scope of antibacterial and antifungal activities [120]. At that time, the substances with condensed hydroxy groups **90** had higher activity than those with free hydroxy groups **91**. The biocompatibility of the series leaders was similar to or higher than that of the common antiseptics (BAC, CPC, OCT, PHMB). Furthermore, Vereshchagin presented a synthetic route and microbiological study of pentaerythritol bis-QACs as OCT analogues **92** [135]. The salts were active towards MRSA and *E. coli* (Figure 29).

An overview of the antibacterial activity of bis-QACs, analyzed in the review, is shown in Table 4.

#### 2.2.4. Dicationic Ionic Liquids

**Figure 30:**
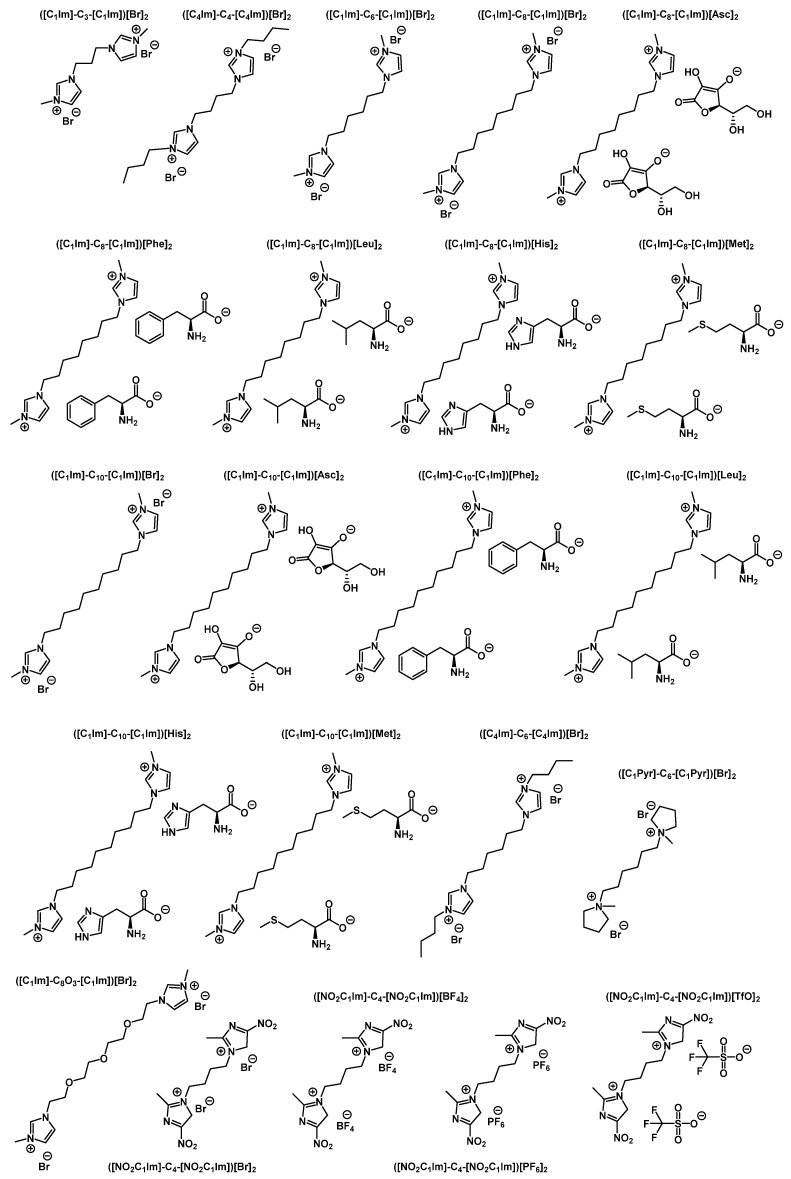
Examples of dicationic ILs with tested antimicrobial activity. The numbers of substances correspond to those in Table 5.

A number of dicationic ILs have been tested for their antimicrobial activity (see Figure 30, Table 5, and Table S3 for several examples) [90,136,137,138,139]. The high bactericidal activity of some of these ILs (in particular, nitro-substituted imidazolium salts) suggests their possible medical applications (see Table 5).

#### 2.2.5. Multiple-Charged QACs (Multi-QACs)

Multi-QACs are salts with three or more charged nitrogen atoms in one molecule [8]. This biocide group is rather underexplored compared to mono- and bis-QACs, probably because of the more complicated synthesis and the lack of low-cost platforms for multicharged QAC structures.

**Figure 31:**
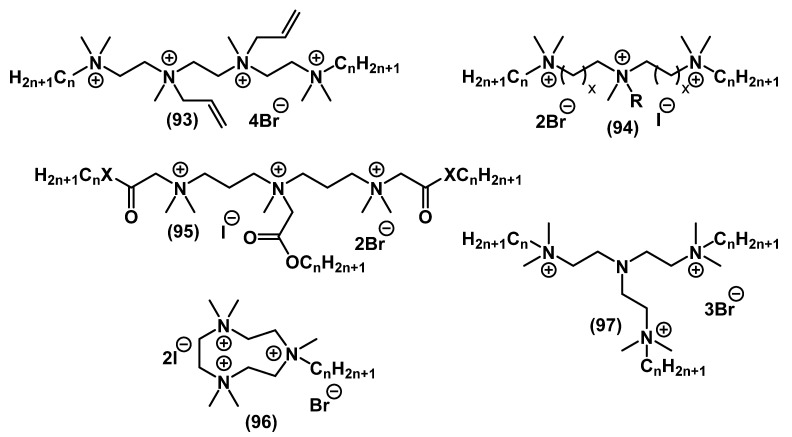
Alkyl multi-QACs.

Wuest and Minbiole developed a simple synthetic route for obtaining tris- and tetra-QACs on the basis of polyamine platforms **93**-**97** (Figure 31) [71,72,76,140]. The activity of multi-QACs was significantly higher than that of mono-QACs but was comparable to that of bis-QACs.

**Figure 32:**
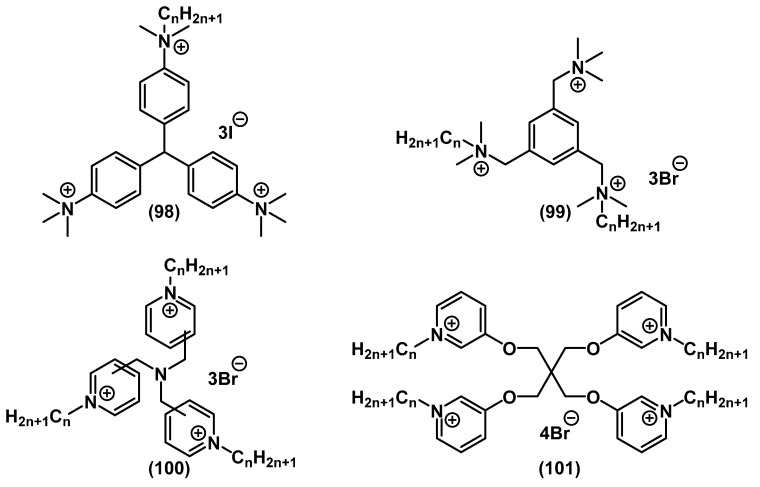
Multi-QACs with aromatic fragments.

Several multi-QACs with aromatic fragments in the structure were also obtained (Figure 32). Forman and colleagues demonstrated that tris-derivatives of crystal violet with one alkyl tail **98** had lower activity than mono-QACs. However, analogs containing ethyl groups at the charged nitrogen instead of methyl groups were more active [107]. Gallagher and colleagues found that tris-QACs with two alkyl tails **99** were more effective against Gram-negative bacteria than tris-QACs with one alkyl tail [141,142]. Tris-pyridinium salts **100** [143] and tetrapyridinium salts **101** [144] also comprised an efficient group of biocides with a broad spectrum of action and surpassed the activity of the well-known pyridinium antiseptic CPC **5** several times.

An overview of the antibacterial activity of multiple QACs, analyzed in the review, is shown in Table 6.

#### 2.2.6. Poly-Charged QACs (Poly-QACs)

Polymer structures with quaternary nitrogen occupy a large niche in the field of cationic biocides. QACs exhibiting antimicrobial activity can be incorporated into polymer structures in several ways [49]:

Ring-opening polymerization. Chain-growth polymerization, in which one end of the polymer chain carries an active site for adding cyclic monomers. The terminal groups of the resulting polymer depend on the initiator used and the termination reaction [145].

Controlled radical polymerization. Continuous polymerization includes several stages: Initiation, growth, and chain termination [146].

Click reaction. Polymerization that utilizes methods of click chemistry [147].

**Figure 33:**
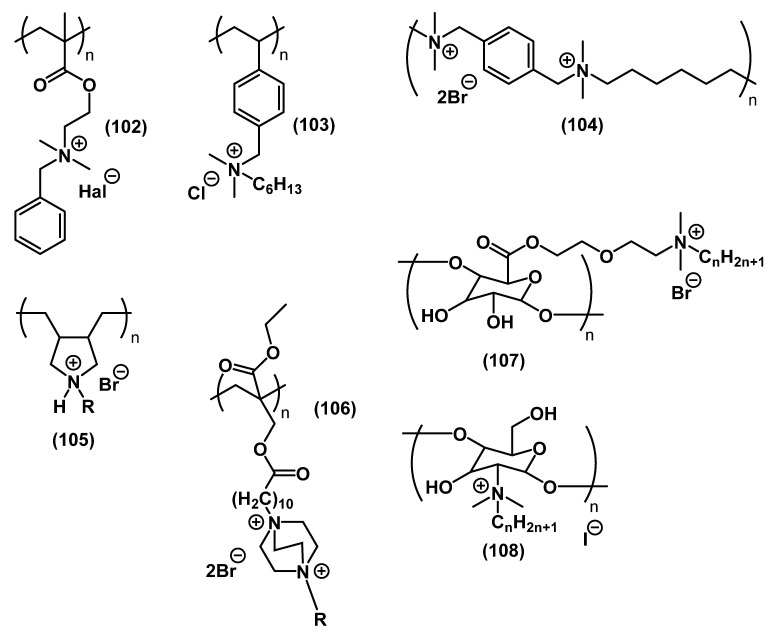
Spectrum of biologically active homogeneous poly-QACs.

**Figure 34:**
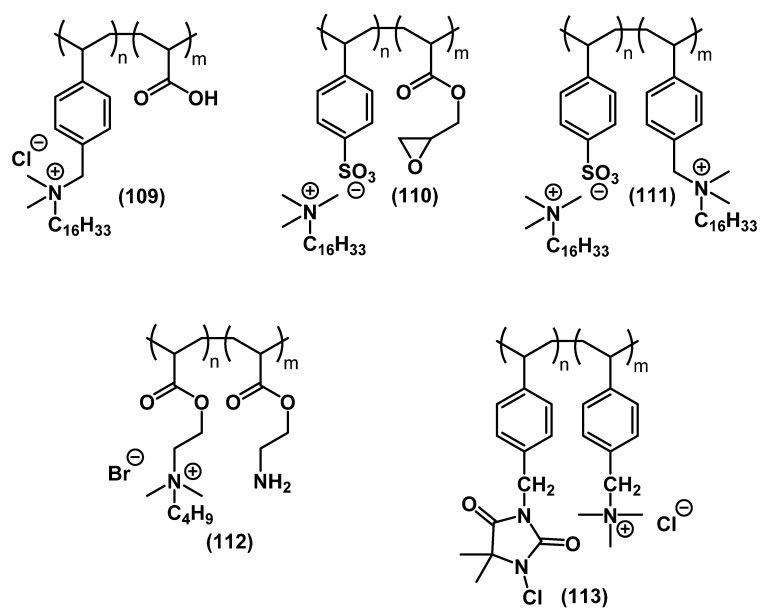
Copolymer poly-QACs.

Similar to other types of QACs, the structure of poly-QACs can vary depending on the monomer composition (homogeneous poly-QACs (Figure 33) in the case of the same monomers, or copolymers (Figure 34) in the case of different monomers) and the polymerization type.

Lu and colleagues studied the biological properties of poly-QACs with benzyl substituents and ether groups in side chain **102** [148]. The activity of the polyderivatives was significantly higher than that of the corresponding monomers; it increased upon increasing the length of the alkyl substituent. Guo and colleagues compared polymers with quaternary nitrogen in the side **103** and main **104** chains [149]. The presence of charged nitrogen atoms in the main polymer chain enhanced the antibacterial effect on Gram-positive and Gram-negative bacteria by several times. The carbohydrate-based poly-QACs obtained by Badawy’s **108** [150] and Shaban’s **107** [151] groups also exhibited biocidal activity. Polymer salts consisting of monomers with DABCO-containing heterocyclic QACs **106** were obtained by Mathias’ group [152]. Researchers observed an increase in bactericidal activity with the growth of alkyl chains. It should be noted that the monomer did not exhibit antibacterial activity. Polymerization may be the key to achieving the required biocidal effect for inactive QAC molecules. Timofeeva and colleagues developed an approach to the synthesis of quaternary poly(diallyldialkylammonium) salts with various substituents **105** [153]. The researchers noted that the antibacterial effect, but not the antifungal effect, became more pronounced upon increasing the mass of the polymer.

Kallitsis and colleagues studied single- **109**-**110** and two-charged **111** copolymeric QACs in their work [154,155]. The peculiarity of this study was in the fact that the polymer chain in one of the target compounds **110** was an anion, while the cation was a conventional mono-QAC alkyl cation of CTAB type **2**, whereas compound **111** was poly-QAC bearing both cations and anions. This composition had a positive impact on the biocidal effect against a wide range of bacteria. The optimal structure was established as 75% ionic and 25% covalent bonds of the polymer with QAC. Jie and colleagues combined the QAC and *N*-chloramine **113** molecules in one polymer [128]. A similar successful approach was pursued by Liu and colleagues [56,57,58]. Bai and colleagues synthesized a polymer combining amino and QAC groups **112**, which showed excellent bacteriostatic potential [156].

The diversity of homogeneous and copolymeric QACs is very high and is beyond the scope of this review; only exemplary biologically active representatives of this class are presented here. More detailed information on poly-QACs can be found in other reviews [44,47,49,50,157,158,159].

An overview of the antibacterial activity of poly-QACs, analyzed in the review, is shown in Table 7.

#### 2.2.7. Polyionic Liquids

**Figure 35:**
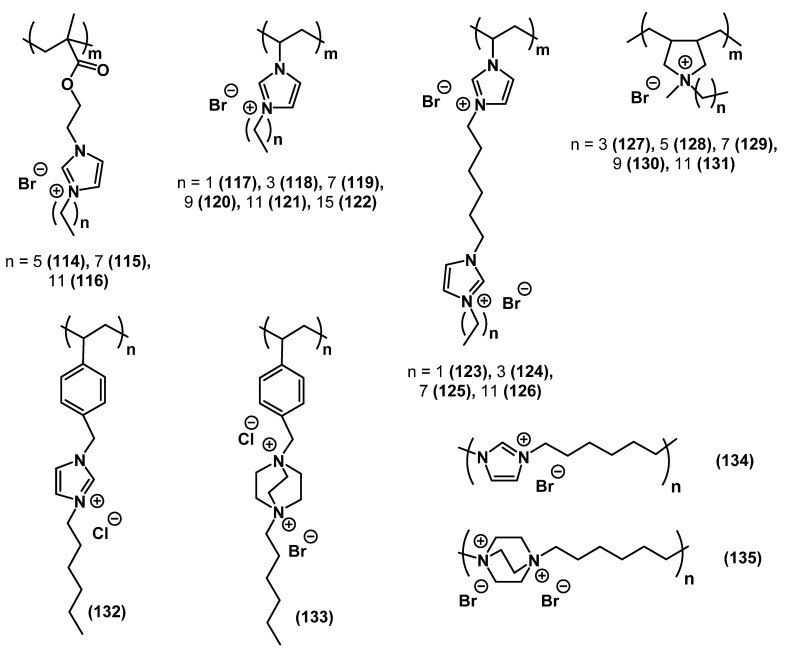
Examples of poly-ILs with tested antimicrobial activity. The numbers of substances correspond to those in Table 8.

According to the strict definition, poly-ILs are ionic polymers with complete ionicity [161]. However, ionic polymers with lower levels of ionicity are often considered poly-ILs in publications. In recent years, poly-ILs have been extensively studied as advantageous materials for antibacterial coatings and surfaces [89,162,163,164,165,166,167,168,169]. Exemplary poly-ILs with tested antibacterial activity are listed in Table 8 and Figure 35. Note that the table includes substances **103** and **104**, which are also considered poly-(QACs).

Antibacterial coatings on the basis of 3-(2-(methacryloyloxy)ethyl)-1-alkylimidazolium ILs showed high bactericidal activity against *E. coli* (see entries **114**-**116** in Table 8) [162]. In the case of 1-alkyl-3-vinylimidazolium-based poly-ILs, the alkyl side chain length and charge density were directly related to the antimicrobial activity against *E. coli* and *S. aureus* (see entries **117**-**119**, **121**, and **123**-**126** in Table 8) [164]. In contrast, the bactericidal activity of the corresponding poly-IL membranes increased upon increasing the charge density but decreased upon increasing the alkyl chain length. A similar picture was observed for pyrrolidinium-based ILs and membranes [89]. The homopolymeric ILs were active against *S. aureus* and *E. coli*, and their antimicrobial activity increased upon increasing the alkyl side chain length in the monomer (see entries **123**-**126** and **127**-**131** in Table 8). The opposite was observed for the corresponding poly-IL-based membranes, which also demonstrated good hemocompatibility and low cytotoxicity. Of note, nanoparticles on the basis of 1-alkyl-3-vinylimidazolium poly-ILs showed significantly higher antimicrobial activity than the original poly-ILs [170] (see entries **119**-**122** in Table 8).

(2-Ethylhexyl)ethylenediaminium bis(trifluoromethanesulfonyl)imide-loaded ionogel surface coatings efficiently inhibited the growth of various microorganisms, including those from the ESKAPE list, and prevented the formation of biofilms [163]. Microneedle patches on the basis of salicylic acid-containing API-poly-IL were successfully tested in the treatment of *Propionobacterium acnes* skin infections [165]. Ionic graft copolymers on the basis of [2-(methacryloyloxy)ethyl]trimethylammonium chloride were studied as possible delivery systems for ionic drugs (*p*-aminosalicylate and clavunate) [171]. IL-grafted wound dressings on the basis of 1-vinyl-3-methylimidazolium bromide demonstrated good antimicrobial activity and low cytotoxicity [172,173].

#### 2.2.8. QAC-Containing Bactericidal Coatings

QACs also find application in the composition of bioactive materials and antibacterial coatings. This topic is more relevant than ever due to the growing part of the paint and coatings industry in the biocide market. Thus, research on the application of QACs at surfaces continues to expand.

Antimicrobial films based on surface-modified microfibrillated cellulose grafted with mono-QACs showed high antibacterial activity against *S. aureus* and *E. coli* even at low concentrations [174]. Silica nanoparticles functionalized with quaternary ammonium silane inhibited the growth of Gram-negative bacteria due to the synergistic effect of hydrophobicity and antibacterial activity [175]. QACs with *N*-halamine coated onto cotton fibers were active against *S. aureus* [176,177]. Similarly, the combination of these biocides was highly effective in macroporous cross-linked antimicrobial polymeric resin [160]. An antibacterial coating of immobilized QACs tethered on hyperbranched polyuria demonstrated high contact-killing efficacies toward adhering staphylococci [178]. Antimicrobial acrylic coatings with a QAC-containing perfluoroalkyl monomer were synthesized by using a self-stratification strategy via one-step UV curing [179]. Polyvinylidene fluoride membranes modified by QACs possess antibiofouling effects [180]. Bacterial cellulose incorporated with QACs showed strong and long-term antimicrobial activity against *S. aureus* and *S. epidermidis* [181]. QAC-based silver nanocomposites demonstrated synergistic antibiofilm properties along with a low hemolysis rate [182]. More examples of QACs immobilized on material surfaces with antibacterial activities can be found elsewhere [45,47,49,159].

#### 2.2.9. Ionic Liquid-Containing Bactericidal Coatings

Usage in bactericidal surface coatings seems one of the most promising applications of antibacterial ILs in medicine and other areas. Thus, the number of publications on the topic has been increasing steadily in recent years. As already mentioned above, ILs are proposed to be used as components of ionogels, films, and membranes that demonstrate considerable antimicrobial and antifouling activities (see, e.g., [89,93,163]). Cellulose nanofibers grafted with ammonium ILs and silver ions demonstrated significant antimicrobial activity against *S. aureus* MRSA and *E. coli* [183]. Zinc ion-coordinated poly-IL membranes with bactericidal properties were efficiently used for wound healing [184]. A conductive hydrogel wound dressing composed of a poly-IL (1-vinyl-3-(aminopropyl)imidazolium tetrafluoroborate) and konjac glucomannan demonstrated long-lasting bactericidal activity against *S. aureus* and *E. coli* [185]. Similarly, promising results were obtained with a poly-IL (1-vinyl-3-butylimidazolium bromide)/poly(vinyl alcohol) wound dressing [172], a reusable 1-vinyl-3-butylimidazolium bromide-grafted cotton gauze wound dressing [173], and molecular brushes with 3-(12-mercaptododecyl)-1-methylimidazolium bromide [186]. Composite membranes composed of bacterial cellulose and cholinium poly-ILs with amino acid anions were active against Gram-negative and Gram-positive bacteria and fungi [187]. Poly(vinylidene fluoride) (PVDF) materials grafted with ILs (1-vinyl-3-butylimidazolium chloride, 1-vinyl-3-ethylimidazolium tetrafluoroborate) showed activity against both common bacteria and “superbugs” [188]. Calcium phosphate–IL (1-alkyl-3-methylimidazolium chloride) materials with bactericidal properties were proposed to be used for implants [189]. Halloysite nanotubes functionalized with various ILs demonstrated antimicrobial activity [190].Coatings based on dicationic imidazolium ILs efficiently inhibited bacterial growth on titanium surfaces [191]. TiO_2_ nanomaterials coated with poly-IL brushes on the basis of imidazolium ILs demonstrated antibacterial and antifouling properties [192]. Cholinium salicylate-containing gelatin films with bactericidal activity were proposed to be used in food packaging [193]. In addition, 1-butyl-3-methylimidazolium bis(trifluoromethanesulfonyl)imide ([C_4_Mim][NTf_2_]) was tested as a bactericidal additive in orthodontic adhesive and was shown to reduce biofilm formation [194].

## 3. Conclusions

Despite the vast diversity of the available QAC structures, there are certain structural criteria designating the biocidal activity of the compounds.

Usually, the optimal alkyl tail length is within C_10_-C_14_; it can vary depending on the number of charges: C_12_ and longer for mono-QACs and C_10_-C_12_ for bis-QACs. Nevertheless, in some series of compounds, those with tails of C_10_ and shorter demonstrated the highest activity. This observation suggests that the optimal chain length is specific for each set of structures and is related to the other fragments of the molecule.

In general, QACs with two or more charges (bis-QACs, multi-QACs, poly-QACs) have superior biocidal effects compared to mono-QACs. Moreover, many mono-QACs show little or no activity against Gram-negative bacteria. However, the addition of the second charged nitrogen without an alkyl chain does not always increase the activity, whereas the addition of the second and third alkyl chains increases the toxicity. The introduction of ether or amide bridges into QACs decreases both the toxicity and activity of the corresponding substances.

The combination of two bactericidal fragments with different mechanisms of action in one QAC has been proven to be a successful approach. These biocides have antibacterial and antifungal effects on a wide range of pathogens.

The assessment of the direct relation between the presence of aromatic and heterocyclic fragments/substituents in QAC molecules and their activity is complicated because this factor is highly specific for some structures. Relatively speaking, pyridine QACs, especially bis-pyridine salts with broad antibacterial/antifungal activity, are the most advanced and promising among all heterocyclic QACs. Aromatic structures are often used in QACs due to their strong reactivity. They can be spacers, substituents, tails, head parts, etc.

In 2016, in his report on antibacterial resistance, O’Neill predicted that by 2050, 10 million people would die because of resistant bacteria annually [195]. Moreover, SARS-CoV-2 aggravated the issue. During the current pandemic, antibacterial drugs are being used rather indiscriminately. It should be expected that the threat from resistant bacteria will increase significantly in the next few years. To avert this danger, the next generation of antibacterial drugs, including QACs, should be developed in the near future.

In this review, we analyze some of the structure–activity dependences and provide a general overview of the current situation in the research on antimicrobial QACs. In addition, a brief overview of the antimicrobial activities of various subclasses of ionic liquids, which are often considered advantageous antimicrobial agents, is also provided. We hope that it will serve as a highlight for future studies on these classes of biocides.

## Figures and Tables

**Table 1:** Antimicrobial activity of mono-QACs *.

Series/ Compound	Strain	MIC, mg⋅L^−1^	MBC, mg⋅L^−1^	Method	Notes	Ref.
**10**	*E. faecalis* ATCC 29212	8	16	Microtiter dilution		[52]
	*S. aureus* ATCC 25923	2	4			
	*E. coli* ATCC 25922	64	64			
	*P. aeruginosa* ATCC 27853	250	250			
**11**	*E. faecalis* ATCC 29212	4	8	Microtiter dilution	Active towards herpes simplex virus	[52]
	*S. aureus* ATCC 25923	2	2			
	*E. coli* ATCC 25922	125	250			
	*P. aeruginosa* ATCC 27853	250	1000			
**12**	*E. faecalis* ATCC 29212	1	4	Microtiter dilution		[52]
	*S. aureus* ATCC 25923	<0.25	1			
	*E. coli* ATCC 25922	250	250			
	*P. aeruginosa* ATCC 27853	500	500			
**13**	*E. faecalis* ATCC 29212	<0.25	8	Microtiter dilution		[52]
	*S. aureus* ATCC 25923	<0.25	4			
	*E. coli* ATCC 25922	1000	>2000			
	*P. aeruginosa* ATCC 27853	1000	>2000			
**14**	*S. aureus* ATCC 6538	1.05 μM		Broth microdilution		[54]
	*E. coli* CNCTC 377/79	2.2 μM				
	*C. albicans* CCM 8186	1.05 μM				
**15**	*S. aureus* ATCC 6538	5.2 μM		Broth microdilution		[54]
	*E. coli* CNCTC 377/79	41.2 μM				
	*C. albicans* CCM 8186	164.9 μM				
**16**	*S. aureus* ATCC 6538	5.4 μM		Broth microdilution		[53]
	*E. coli* CNCTC 377/79	144.1 μM				
	*C. albicans* CCM 8186	5.4 μM				
**17**	*S. aureus* ATCC 6538	75% (percent of inhibition, 250 mg⋅L^−1^)		Broth microdilution	Active towards bacterial biofilms	[55]
	*E. coli* CNCTC 377/79	80% (percent of inhibition, 250 mg⋅L^−1^)				
**18**	MRSA 70065		3 min (Tk)/141 μM			[58]
	*E. coli* ATCC 25922		3 min (Tk)/141 μM			
	multidrug-resistant (MDR) *P. aeruginosa* 73104		<1 min (Tk)/141 μM			
	wild-type *P. aeruginosan* PA01		3 min (Tk)/141 μM			
**19**	methicillin-resistant *S. aureus* (MRSA) 70065		3 min (Tk (time to kill))/141 μM			[58]
	*E. coli* ATCC 25922		3 min (Tk)/141 μM			
	multidrug-resistant (MDR) *P. aeruginosa* 73104		5 min (Tk)/141 μM			
	wild-type *P. aeruginosan* PA01		5 min (Tk)/141 μM			
**20**	*S. aureus*	99% (reduction, contact time–5 min, 20 ppm)		AATCC test		[59]
	*E. coli*	100% (reduction, contact time–5 min, 20 ppm)				
**21**	*S. aureus*	6.25	6.25	Broth tube dilution		[61]
	*a-H-tococcus*	12.5	12.5			
	*b-H-tococcus*	1.56	3.125			
	*E. coli*	25	25			
	*P. aeruginosa*	25	25			
	*P. vulgaris*	25	25			
	*C. albicans*	6.25	6.25			
	*C. mandshurica*	1.56	6.25			
	*P. piricola*	3.125	3.125			
	*A. niger*	3.125	6.25			
**22**	*S. aureus*	22.4 mm (IZ, 500 ppm)		Disk diffusion		[60]
	*B. subtilis*	17 mm (IZ, 500 ppm)				
	*E. coli*	24.1 mm (inhibition zone, 500 ppm)				
**23**	*S. aureus*	6.25	6.25	Broth tube dilution		[61]
	*a-H-tococcus*	6.25	6.25			
	*b-H-tococcus*	1.56	1.56			
	*E. coli*	12.5	12.5			
	*P. aeruginosa*	25	25			
	*P. vulgaris*	12.5	12.5			
	*C. albicans*	6.25	6.25			
	*C. mandshurica*	3.125	3.125			
	*P. piricola*	1.56	1.56			
	*A. niger*	6.25	6.25			
**24**	*S. aureus*	12.5	25	Broth tube dilution		[61]
	*a-H-tococcus*	12.5	12.5			
	*b-H-tococcus*	6.25	6.25			
	*E. coli*	25	25			
	*P. aeruginosa*	50	50			
	*P. vulgaris*	25	25			
	*C. albicans*	12.5	12.5			
	*C. mandshurica*	12.5	12.5			
	*P. piricola*	6.25	6.25			
	*A. niger*	12.5	12.5			
**25**	*S. aureus* ATCC 209p	12.5 μM		Broth microdilution		[62]
	*B. cereus* ATCC 8035	401 μM				
	*C. albicans* 855-653	200 μM				
**27**	*S. aureus* ATCC 209p	6.9 μM		Broth microdilution		[62]
	*B. cereus* ATCC 8035	28.0 μM				
	*C. albicans* 855-653	222 μM				
**29**	*S. aureus*	14.3 mm (IZ, 500 ppm)		Disk diffusion		[63]
**30**	*S. aureus* C1947	0.49 μM	1.22 μM	Broth microdilution	Active towards varicella-zoster virus	[64]
	MRSA C1926	1.47 μM	1.95 μM			
	Vancomycin-reristant *enterococci* S2484	1.95 μM	2.93 μM			
	*Y. bercovieri* CNCTC6230	1.95 μM	2.45 μM			
	*A. baumannii* J3474	2.93 μM	2.93 μM			
	*E. coli* A1235	5.86 μM	5.86 μM			
	*K. pneumoniae* C1950	7.81 μM	7.81 μM			
	*S. maltophilia* J3552	5.86 μM	5.86 μM			
	Extended-spectrum β-lactamase-producing *K. pneumonie* C1934	7.81 μM	15.63 μM			
	*C. parapsilosis sensu stricto*EXF-8411	100 μM				
	*R. mucilaginosa* EXF-8417	100 μM				
	*E. dermatitidis* EXF-8470	30 μM				
	*A. melanogenum* EXF-8432	30 μM				
	*B. dimerum* EXF-8427	500 μM				
	*P. chrysogenum* EXF-1818	300 μM				
	*A. versicolor* EXF-8692	65 μM				
**32**	*S. aureus* ATCC29213	2		Broth microdilution		[66]
	*S. epidermidis* (clinical isolate)	2				
	*M. luteus* (clinical isolate)	2				
	*E. coli* ATCC25922	>64				
	*S. typhimurium* TA100	>64				
	*P. aeruginosa* ATCC27853	>64				
**33**	*S. aureus* ATCC29213	4		Broth microdilution		[66]
	*S. epidermidis* (clinical isolate)	4				
	*M. luteus* (clinical isolate)	2				
	*E. coli* ATCC25922	>64				
	*S. typhimurium* TA100	4				
	*P. aeruginosa* ATCC27853	>64				
**34**	*S. aureus* ATCC29213	0.5		Broth microdilution		[66]
	*S. epidermidis* (clinical isolate)	0.5				
	*M. luteus* (clinical isolate)	0.5				
	*E. coli* ATCC25922	2				
	*S. typhimurium* TA100	0.5				
	*P. aeruginosa* ATCC27853	>64				
**35**	*S. aureus* ATCC29213	0.5		Broth microdilution	Non-genotoxic and non-mutagenic	[70]
	*S. epidermidis* (clinical isolate)	2				
	*M. luteus* (clinical isolate)	1				
	*E. coli* ATCC25922	8				
	*P. aeruginosa* ATCC27853	8				
**36**	*S. aureus* ATCC 29213	4	8	Broth microdilution	Active towards bacterial, fungi and mixed biofilms	[69]
	*B. subtilis* 168	4	8			
	*S. epidermidis*	4	8			
	*E. coli* MG1655	16	16			
	*K. pneumoniae*	>64	>64			
	*P. aeruginosa* ATCC 27853	64	64			
**37**	*S. aureus*	2 μM		Broth microdilution		[76]
	*E. faecalis*	4 μM				
	*E. coli*	16 μM				
	*P. aeruginosa*	63 μM				
	MRSA 300-0114	2 μM				
	MRSA ATCC 33592	2 μM				
**38**	*S. aureus*	0.5 μM		Broth microdilution	Natural derivatives	[74]
	MRSA 300-0114	2 μM				
	MRSA ATCC 33592	4 μM				
	*E. faecalis*	1 μM				
	*E. coli*	8 μM				
	*P. aeruginosa*	8 μM				
**39**	*S. aureus*	1 μM		Broth microdilution	Natural derivatives	[74]
	MRSA 300-0114	4 μM				
	MRSA ATCC 33592	2 μM				
	*E. faecalis*	1 μM				
	*E. coli*	4 μM				
	*P. aeruginosa*	63 μM				
**40**	*S. aureus*	1 μM		Broth microdilution		[72]
	MRSA 300-0114	4 μM				
	MRSA ATCC 33592	2 μM				
	*E. faecalis*	1 μM				
	*E. coli*	4 μM				
	*P. aeruginosa*	63 μM				
**41**	*S. aureus* SH1000	1 μM		Broth microdilution		[75]
	*E. faecalis* OG1RF	16 μM				
	*E. coli* MC4100	16 μM				
	*P. aeruginosa* PAO1-WT	16 μM				

* IZ, inhibition zone; Tk, time to kill; MIC, minimum inhibitory concentration; MBC, minimum bactericidal concentration; MRSA, methicillin-resistant *S. aureus*; only leader compounds from the series are listed in the table.

**Table 2:** Antimicrobial activity of common ILs *.

IL	Acronym	Species	MIC, μg mL^−1^	MBC, μg mL^−1^	Method	Notes	Ref.
1-Ethyl-3-methylimidazolium bromide	[C_2_Mim][Br]	*E. coli* ATCC 25922	>5000 µM		Broth microdilution	*E. coli* TEM CTX M9, CTX M2, and AmpC MOX2 are ampicillin-resistant strains.	[82]
		*E. coli* TEM CTX M9	5000 µM				
		*E. coli* CTX M2	>5000 µM				
		*E. coli* AmpC MOX2	>5000 µM				
		*K. pneumoniae* (clinical isolate)	>5000 µM				
		*S. aureus* ATCC 25293	50 µM				
		*S. epidermidis* (clinical isolate)	5000 µM				
		*E. faecalis* (clinical isolate)	>5000 µM				
1-Butyl-3-methylimidazolium bis(trifluoromethanesulfonyl)imide	[C_4_Mim][NTf_2_]	*P. aeruginosa* PTCC 1310	3120	3120	Agar disk diffusion/agar well diffusion	Anti-adhesive activity ^a^	[94]
		*S. aureus* PTCC 1112	3120	3120			
		*E. coli* PTCC 1338	<40	48			
		*B. cereus* PTCC 1015	3120	3120			
		*S. typhimurium* (wild type)	390	390			
		*K. pneumonia* PTCC 1290	3120	3120			
		*B. subtilis* PTCC 1715	3120	3120			
1-Octyl-3-methylimidazolium bromide	[C_8_Mim][Br]	*M. luteus* ATCC 9341	R		Broth microdilution	R, resistant at the highest concentration tested (256 μg mL−1).	[81,87]
		*S. epidermidis* ATCC155-1	930 μM				
		*S. aureus* ATCC 25178	R				
		*S. aureus* 209 KCTC1916	64				
		*S. aureus* R209 KCTC1928	250				
		*E. coli* ATCC 27325	R				
		*E. coli* KCTC1924	64				
		*K. pneumonia* ATCC 9721	R				
		*P. aeruginosa* ATCC 9721	R				
		*C. albicans* ATCC10231	R				
		*C. albicans* KCTC19401	250				
		*B. subtilis* ATCC663	R				
		*B. subtilis* KCTC1914	500				
		*S. typhimurium* KCTC1926	500				
		*C. regularis*	500				
1-Octyl-3-methylimidazolium nitrate	[C_8_Mim][NO_3_]	*S. aureus*	97	97	Agar disk diffusion/agar well diffusion	Anti-adhesive activity ^a^	[95]
		*K. pneumoniae*	780	780			
		*S. typhimurium*	780	780			
		*P. aeruginosa*	1560	1560			
		*E. coli*	39	39			
		*B. tequilensis*	19	19			
		*B. subtilis*	19	19			
1-Decyl-3-methylimidazolium chloride	[C_10_Mim][Cl]	*S. aureus* ATCC 29213	40 μM (MBEC 2415 μM)	643 μM	Broth microdilution, MBEC assay	Deletions ΔrfaC, ΔrfaL, and ΔrfaG affect the cell surface hydrophobicity and membrane permeability.	[81,85,86]
		*E-MRSA 15*	40 μM (MBEC 1207 μM)	321 μM			
		*MRSA* (clinical strain 201)	160 μM (MBEC 4829 μM)	643 μM			
		*S. aureus* 209 KCTC1916	16				
		*S. aureus* R209 KCTC1928	32				
		*S. epidermidis* ATCC 12228	40 μM	644 μM			
		*S. epidermidis* ATCC 35984	40 μM (MBEC 4829 μM)	160 μM			
		*E. coli* NCTC 8196	321 μM (MBEC 9659 μM)	1287 μM			
		*E. coli* KCTC1924	8				
		*E. coli* BW25113 (wild-type)	188.9				
		*E. coli* JW3596 (ΔrfaC)	100				
		*E. coli* JW3597 (ΔrfaL)	155				
		*E. coli* JW3606 (ΔrfaG)	67.5				
		*P. aeruginosa* PA01	>1287 μM (MBEC 2415 μM)	>1287 μM			
		*K. aerogenes* NCTC 7427	643 μM (MBEC 19318 μM)	1287 μM			
		*B. cenocepacia* J2315	1287 μM (MBEC 19318 μM)	1287 μM			
		*P. mirabilis* NCTC 12442	1287 μM (MBEC 9659 μM)	1287 μM			
		*C. tropicalis* NCTC 7393	321 μM (MBEC 19318 μM)	321 μM			
		*B. subtilis* KCTC1914	125				
		*S. typhimurium* KCTC1926	125				
		*C. albicans* KCTC19401	250				
		*C. regularis*	250				
1-Decyl-3-methylimidazolium bromide	[C_10_Mim][Br]	*M. luteus* ATCC 9341	R		Broth microdilution	R, resistant at the highest concentration tested (256 μg mL−1).	[87]
		*S. epidermidis* ATCC155-1	844 μM				
		*S. aureus* ATCC 25178	106 μM				
		*E. coli* ATCC 27325	R				
		*K. pneumonia* ATCC 9721	R				
		*P. aeruginosa* ATCC 9721	R				
		*C. albicans* ATCC10231	R				
		*B. subtilis* ATCC6633	422 μM				
1-Dodecyl-3-methylimidazolium chloride	[C_12_Mim][Cl]	*S. aureus* ATCC 29213	18 μM (MBEC 272 μM)	36 μM	Broth microdilution, MBEC assay	Deletions ΔrfaC, ΔrfaL, and ΔrfaG affect the cell surface hydrophobicity and membrane permeability.	[85,86]
		*E-MRSA 15*	18 μM (MBEC 272 μM)	73 μM			
		*MRSA* (clinical strain 201)	36 μM (MBEC 545 μM)	290 μM			
		*S. epidermidis* ATCC 12228	36 μM	145 μM			
		*S. epidermidis* ATCC 35984	36 μM (MBEC 272 μM)	73 μM			
		*E. coli* NCTC 8196	73 μM (MBEC 1089 μM)	73 μM			
		*E. coli* BW25113 (wild-type)	47.3				
		*E. coli* JW3596 (ΔrfaC)	10.1				
		*E. coli* JW3597 (ΔrfaL)	45.4				
		*E. coli* JW3606 (ΔrfaG)	11.4				
		*P. aeruginosa* PA01	580 μM (MBEC 1089 μM)	1161 μM			
		*K. aerogenes* NCTC 7427	73 μM (MBEC 2179 μM)	145 μM			
		*B. cenocepacia* J2315	290 μM (MBEC 2179 μM)	580 μM			
		*P. mirabilis* NCTC 12442	580 μM (MBEC 4357 μM)	1161 μM			
		*C. tropicalis* NCTC 7393	73 μM (MBEC 8714 μM)	73 μM			
1-Dodecyl-3-methylimidazolium bromide	[C_12_Mim][Br]	*M. luteus* ATCC 9341	R		Broth microdilution	R, resistant at the highest concentration tested (256 μg mL−1).	[81,87,90,91]
		*S. epidermidis* ATCC155-1	193 μM				
		*S. epidermidis* ATCC 35984	2.5				
		*S. aureus* ATCC 25178	97 μM				
		*S. aureus* ATCC 6538	2.5	40			
		*S. aureus* 209 KCTC1916	4				
		*S. aureus* R209 KCTC1928	8				
		*E. coli* ATCC 27325	386 μM				
		*E. coli* ATCC 25922	20	10			
		*E. coli* KCTC1924	8				
		*K. pneumonia* ATCC 9721	773 μM				
		*K. pneumonia* ATCC BAA-1705	80				
		*P. aeruginosa* ATCC 9721	R				
		*P. aeruginosa* ATCC 27853	160	20			
		*C. albicans* ATCC10231	R				
		*B. subtilis* ATCC6633	48 μM				
		*B. subtilis* KCTC1914	8				
		*S. typhimurium* KCTC1926	32				
		*A. baumannii* AB01	80				
		*E. faecalis* ATCC 29212	5	40			
		*C. albicans* KCTC19401	32				
		*C. regularis*	16				
1-Dodecyl-3-methylimidazolium iodide	[C_12_Mim][I]	*S. aureus* V329	0.31 μM	5 μM	Broth microdilution	Potent anti-biofilm activity (higher against *S. aureus*)	[98]
		*P. aeruginosa* PAO1	125 μM	250 μM			
1-Tetradecyl-3-methylimidazolim chloride	[C_14_Mim][Cl]	*S. aureus* ATCC 29213	16 μM (MBEC 124 μM)	66 μM	Broth microdilution, MBEC assay	Deletions ΔrfaC, ΔrfaL, and ΔrfaG affect the cell surface hydrophobicity and membrane permeability.	[81,85,86]
		*E-MRSA 15*	16 μM (MBEC 248 μM)	66 μM			
		*MRSA* (clinical strain 201)	16 μM (MBEC 124 μM)	66 μM			
		*S. aureus* 209 KCTC1916	4				
		*S. aureus* R209 KCTC1928	4				
		*S. epidermidis* ATCC 12228	7.75 μM	33 μM			
		*S. epidermidis* ATCC 35984	7.75 μM (MBEC 124 μM)	33 μM			
		*E. coli* NCTC 8196	33 μM (MBEC 124 μM)	33 μM			
		*E. coli* KCTC1924	4				
		*E. coli* BW25113 (wild-type)	14.9				
		*E. coli* JW3596 (ΔrfaC)	2.2				
		*E. coli* JW3597 (ΔrfaL)	15.5				
		*E. coli* JW3606 (ΔrfaG)	3.3				
		*P. aeruginosa* PA01	264 μM (MBEC 496 μM)	264 μM			
		*K. aerogenes* NCTC 7427	33 μM (MBEC 248 μM)	66 μM			
		*B. cenocepacia* J2315	132 μM (MBEC 496 μM)	264 μM			
		*P. mirabilis* NCTC 12442	264 μM (MBEC 1984 μM)	530 μM			
		*C. tropicalis* NCTC 7393	66 μM (MBEC 248 μM)	132 μM			
		*B. subtilis* KCTC1914	4				
		*S. typhimurium* KCTC1926	8				
		*C. albicans* KCTC19401	8				
		*C. regularis*	8				
1-Tetradecyl-3-methylimidazolim bromide	[C_14_Mim][Br]	*M. luteus* ATCC 9341	178 μM		Broth microdilution		[81,87]
		*S. epidermidis* ATCC155-1	6 μM				
		*S. aureus* ATCC 25178	45 μM				
		*S. aureus* 209 KCTC1916	4				
		*S. aureus* R209 KCTC1928	4				
		*E. coli* ATCC 27325	356 μM				
		*E. coli* KCTC1924	4				
		*K. pneumonia* ATCC 9721	356 μM				
		*P. aeruginosa* ATCC 9721	356 μM				
		*C. albicans* ATCC10231	178 μM				
		*B. subtilis* ATCC6633	6 μM				
		*B. subtilis* KCTC1914	4				
		*S. typhimurium* KCTC1926	8				
		*C. albicans* KCTC19401	8				
		*C. regularis*	16				
1-Hexadecyl-3-methylimidazolim chloride	[C_16_Mim][Cl]	*E. coli* BW25113 (wild-type)	7.7		Broth microdilution	The clinical isolates 72A, 72P, and 94P are resistant to fluconazole, amphotericin B, voriconazole and anidulafungin. Deletions ΔrfaC, ΔrfaL, and ΔrfaG affect the cell surface hydrophobicity and membrane permeability.	[86,88]
		*E. coli* JW3596 (ΔrfaC)	3.5				
		*E. coli* JW3597 (ΔrfaL)	8.2				
		*E. coli* JW3606 (ΔrfaG)	3				
		*C. tropicalis* 17A	0.014 (MBEC 0.028)				
		*C. tropicalis* 57A	0.014 (MBEC 0.056)				
		*C. tropicalis* 72A	0.014 (MBEC 0.056)				
		*C. tropicalis* 72P	0.014 (MBEC 0.056)				
		*C. tropicalis* 94P	0.014 (MBEC 0.225)				
		*C. tropicalis* 102A	0.014 (MBEC 0.056)				
1-Hexadecyl-3-methylimidazolim bromide	[C_16_Mim][Br]	*S. aureus* 209 KCTC1916	8		Broth microdilution		[81,97]
		*S. aureus* R209 KCTC1928	4				
		*S. aureus* ATCC 6538	15 µM				
		*E. coli* KCTC1924	8				
		*E. coli* O157:H7 ATCC 43895	10 µM				
		*B. subtilis* KCTC1914	4				
		*S. typhimurium* KCTC1926	4				
		*E. faecium* ATCC 49474	1 µM				
		*K. pneumonia* ATCC 4352	15 µM				
		*C. albicans* KCTC19401	8				
		*C. regularis*	8				
1-Hexyl-2,3-dimethylimidazolium bromide	[C_6_MMim][Br]	*S. aureus* ATCC 6538	23 µM		Broth microdilution		[97]
		*E. coli* O157:H7 ATCC 43895	12 µM				
		*E. faecium* ATCC 49474	9 µM				
		*K. pneumonia* ATCC 4352	15 µM				
*N*-Dodecylpyridinium bromide	[C_12_Py][Br]	*M. luteus* ATCC 9341	R		Broth microdilution	R, resistant at the highest concentration tested (256 μg mL−1).	[87]
		*S. epidermidis* ATCC155-1	49 μM				
		*S. aureus* ATCC 25178	195 μM				
		*E. coli* ATCC 27325	97 μM				
		*K. pneumonia* ATCC 9721	780 μM				
		*P. aeruginosa* ATCC 9721	780 μM				
		*C. albicans* ATCC10231	R				
		*B. subtilis* ATCC6633	24 μM				
*N*-Tetradecylpyridinium bromide	[C_14_Py][Br]	*M. luteus* ATCC 9341	90 μM		Broth microdilution		[87]
		*S. epidermidis* ATCC155-1	6 μM				
		*S. aureus* ATCC 25178	22 μM				
		*E. coli* ATCC 27325	45 μM				
		*K. pneumonia* ATCC 9721	359 μM				
		*P. aeruginosa* ATCC 9721	359 μM				
		*C. albicans* ATCC10231	359 μM				
		*B. subtilis* ATCC6633	6 μM				
*N*-Hexadecylpyridinium chloride	[C_16_Py][Cl]	*E. coli* ATCC 25922	500 μM		Broth microdilution	*E. coli* TEM CTX M9, CTX M2, and AmpC MOX2 are ampicillin-resistant strains.	[81,82]
		*E. coli* TEM CTX M9	500 μM				
		*E. coli* CTX M2	>5000 μM				
		*E. coli* AmpC MOX2	>5000 μM				
		*K. pneumoniae (clinical isolate)*	2500 μM				
		*S. aureus* ATCC 25293	500 μM				
		*S. aureus* 209 KCTC1916	8				
		*S. aureus* R209 KCTC1928	8				
		*S. epidermidis* (clinical isolate)	2500 μM				
		*E. faecalis* (clinical isolate)	500 μM				
		*B. subtilis* KCTC1914	8				
*N*-Hexadecylpyridinium bromide	[C_16_Py][Br]	*S. aureus* ATCC 6538	15 μM		Broth microdilution		[97]
		*E. coli* O157:H7 ATCC 43895	13 μM				
		*E. faecium* ATCC 49474	2 μM				
		*K. pneumonia* ATCC 4352	13 μM				
*N*-Dodecyl-*N*-methylpyrrolidinium bromide	[C_12_C_1_Pyr][Br]	*S. epidermidis* ATCC 35984	10		Broth microdilution		[89,90,91]
		*S. aureus*	15 µM				
		*S. aureus* ATCC 6538	10	80			
		*E. coli*	20 µM				
		*E. coli* ATCC 25922	80	20			
		*P. aeruginosa* ATCC 27853	320	80			
		*K. pneumonia* ATCC BAA-1705	160				
		*A. baumannii* AB01	80				
		*E. faecalis* ATCC 29212	20	40			
*N*-Dodecyl-*N*-hydroxyethylpyrrolidinium chloride	[C_12_HOC_2_Pyr][Cl]	*E. coli* KCTC1924	8		Broth microdilution		[81]
		*S. typhimurium* KCTC1926	16				
		*B. subtilis* KCTC1914	4				
		*C. regularis*	8				
*N*-Dodecyl-*N*-methylpiperidinium bromide	[C_12_C_1_Pip][Br]	*S. epidermidis* ATCC 35984	5		Broth microdilution		[90,91]
		*S. aureus* ATCC 6538	5	80			
		*E. coli* ATCC 25922	40	20			
		*P. aeruginosa* ATCC 27853	320	80			
		*K. pneumonia* ATCC BAA-1705	160				
		*A. baumannii* AB01	320				
		*E. faecalis* ATCC 29212	10	40			
*N*-Dodecyl-*N*-methylmorpholinium bromide	[C_12_C_1_Mor][Br]	*S. epidermidis* ATCC 35984	20		Broth microdilution		[90]
		*S. aureus* ATCC 6538	20				
		*E. coli* ATCC 25922	156.2				
		*P. aeruginosa* ATCC 27853	312.5				
		*E. faecalis* ATCC 29212	40				
Dioctyldimethylammonium chloride	[C_8,8,1,1_N][Cl]	*E. coli* BW25113 (wild-type)	104.2		Broth microdilution	Deletions ΔrfaC, ΔrfaL, and ΔrfaG affect the cell surface hydrophobicity and membrane permeability.	[86]
		*E. coli* JW3596 (ΔrfaC)	20.8				
		*E. coli* JW3597 (ΔrfaL)	91.7				
		*E. coli* JW3606 (ΔrfaG)	22.9				
Trioctylmethylammonium chloride	[C_8,8,8,1_N][Cl]	*E. coli* BW25113 (wild-type)	6.8		Broth microdilution	Deletions ΔrfaC, ΔrfaL, and ΔrfaG affect the cell surface hydrophobicity and membrane permeability.	[86]
		*E. coli* JW3596 (ΔrfaC)	1.7				
		*E. coli* JW3597 (ΔrfaL)	6.9				
		*E. coli* JW3606 (ΔrfaG)	2.5				
Trimethyldecylammonium chloride	[C_1,1,1,10_N][Cl]	*E. coli* BW25113 (wild-type)	119.4		Broth microdilution	Deletions ΔrfaC, ΔrfaL, and ΔrfaG affect the cell surface hydrophobicity and membrane permeability.	[86]
		*E. coli* JW3596 (ΔrfaC)	83				
		*E. coli* JW3597 (ΔrfaL)	130				
		*E. coli* JW3606 (ΔrfaG)	80				
Trimethylhexadecylammonium chloride	[C_1,1,1,16_N][Cl]	*E. coli* BW25113 (wild-type)	13.1		Broth microdilution	Deletions ΔrfaC, ΔrfaL, and ΔrfaG affect the cell surface hydrophobicity and membrane permeability.	[86]
		*E. coli* JW3596 (ΔrfaC)	2.8				
		*E. coli* JW3597 (ΔrfaL)	13				
		*E. coli* JW3606 (ΔrfaG)	3.3				
Trimethylhexadecylammonium bromide (cetyltrimethylammonium bromide)	[C_1,1,1,16_N][Br] (CTAB)	*S. aureus* V329	0.31 μM	5 μM	Broth microdilution	Potent anti-biofilm activity against *S. aureus*	[98]
		*P. aeruginosa* PAO1	125 μM	250 μM			
Dimethyldodecyl(2-hydroxyethyl)ammonium bromide	[HOC_2_C_1,1,12_N][Br]	*B. subtilis* ATCC 6633	15.62		Broth microdilution		[92]
		*M. smegmatis* ATCC 607	15.62				
		*K. pneumonia* ATCC 9997	N.T.				
		*E. faecalis* ATCC 29212	N.T.				
		*VRE* ATCC 51299	62.5				
		*S. aureus*	31.25				
		MRSA CIP 106760	62.5				
		*E. coli* ATCC 25922	62.5				
		*P. aeruginosa* ATCC 27853	250				
		*C. albicans* ATCC 10231	62.5				
		*S. cerevisiae* ATCC 2601	7.81				
Dimethyltetradecyl(2-hydroxyethyl)ammonium bromide	[HOC_2_C_1,1,14_N][Br]	*B. subtilis* ATCC 6633	0.98		Broth microdilution		[92]
		*M. smegmatis* ATCC 607	1.95				
		*K. pneumonia* ATCC 9997	7.82				
		*E. faecalis* ATCC 29212	1.95				
		*VRE* ATCC 51299	1.95				
		*S. aureus*	7.81				
		MRSA CIP 106760	15.62				
		*E. coli* ATCC 25922	15.62				
		*P. aeruginosa* ATCC 27853	125				
		*C. albicans* ATCC 10231	31.25				
		*S. cerevisiae* ATCC 2601	1.95				
Dimethylhexadecyl(2-hydroxyethyl)ammonium bromide	[HOC_2_C_1,1,16_N][Br]	*B. subtilis* ATCC 6633	<0.49		Broth microdilution		[92]
		*M. smegmatis* ATCC 607	3.91				
		*K. pneumonia* ATCC 9997	0.98				
		*E. faecalis* ATCC 29212	0.98				
		*VRE* ATCC 51299	0.98				
		*S. aureus*	1.95				
		MRSA CIP 106760	3.91				
		*E. coli* ATCC 25922	7.81				
		*P. aeruginosa* ATCC 27853	250				
		*C. albicans* ATCC 10231	3.91				
		*S. cerevisiae* ATCC 2601	1.95				
Dimethyloctadecyl(2-hydroxyethyl)ammonium bromide	[HOC_2_C_1,1,18_N][Br]	*B. subtilis* ATCC 6633	1.95		Broth microdilution		[92]
		*M. smegmatis* ATCC 607	3.91				
		*K. pneumonia* ATCC 9997	1.95				
		*E. faecalis* ATCC 29212	1.95				
		*VRE* ATCC 51299	0.98				
		*S. aureus*	1.95				
		MRSA CIP 106760	0.98				
		*E. coli* ATCC 25922	31.25				
		*P. aeruginosa* ATCC 27853	125				
		*C. albicans* ATCC 10231	<0.48				
		*S. cerevisiae* ATCC 2601	<0.48				
Di(2-hydroxyethyl)tetradecylammonium bromide	[(HOC_2_)_2_C_14_NH][Br]	*B. subtilis* ATCC 6633	7.81		Broth microdilution		[92]
		*M. smegmatis* ATCC 607	15.62				
		*K. pneumonia* ATCC 9997	7.81				
		*E. faecalis* ATCC 29212	15.62				
		*VRE* ATCC 51299	7.81				
		*S. aureus*	15.62				
		MRSA CIP 106760	15.62				
		*E. coli* ATCC 25922	31.25				
		*P. aeruginosa* ATCC 27853	N.T.				
		*C. albicans* ATCC 10231	15.62				
		*S. cerevisiae* ATCC 2601	N.T.				
Di(2-hydroxyethyl)decylmethylammonium bromide	[(HOC_2_)_2_C_10,1_N][Br]	*B. subtilis* ATCC 6633	250		Broth microdilution		[92]
		*M. smegmatis* ATCC 607	62.5				
		*K. pneumonia* ATCC 9997	N.A.				
		*E. faecalis* ATCC 29212	N.A.				
		*VRE* ATCC 51299	N.A.				
		*S. aureus*	N.A.				
		MRSA CIP 106760	N.A.				
		*E. coli* ATCC 25922	N.A.				
		*P. aeruginosa* ATCC 27853	N.A.				
		*C. albicans* ATCC 10231	N.T.				
		*S. cerevisiae* ATCC 2601	N.T.				
Di(2-hydroxyethyl)dodecylmethylammonium bromide	[(HOC_2_)_2_C_12,1_N][Br]	*B. subtilis* ATCC 6633	31.25		Broth microdilution		[92]
		*M. smegmatis* ATCC 607	<7.82				
		*K. pneumonia* ATCC 9997	62.5				
		*E. faecalis* ATCC 29212	62.25				
		*VRE* ATCC 51299	62.5				
		*S. aureus*	31.25				
		MRSA CIP 106760	62.5				
		*E. coli* ATCC 25922	125				
		*P. aeruginosa* ATCC 27853	250				
		*C. albicans* ATCC 10231	250				
		*S. cerevisiae* ATCC 2601	31.25				
Di(2-hydroxyethyl)tetradecylmethylammonium bromide	[(HOC_2_)_2_C_14,1_N][Br]	*B. subtilis* ATCC 6633	1.95		Broth microdilution		[92]
		*M. smegmatis* ATCC 607	1.95				
		*K. pneumonia* ATCC 9997	7.82				
		*E. faecalis* ATCC 29212	N.T.				
		*VRE* ATCC 51299	N.T.				
		*S. aureus*	3.91				
		MRSA CIP 106760	1.95				
		*E. coli* ATCC 25922	15.62				
		*P. aeruginosa* ATCC 27853	62.5				
		*C. albicans* ATCC 10231	31.25				
		*S. cerevisiae* ATCC 2601	1.95				
Trioctylmethylphosphonium chloride	[C_8,8,8,1_P][Cl]	*E. coli* BW25113 (wild-type)	6.8		Broth microdilution	Deletions ΔrfaC, ΔrfaL, and ΔrfaG affect the cell surface hydrophobicity and membrane permeability.	[86]
		*E. coli* JW3596 (ΔrfaC)	2.2				
		*E. coli* JW3597 (ΔrfaL)	5.6				
		*E. coli* JW3606 (ΔrfaG)	2.8				
Trihexyltetradecylphosphonium chloride	[C_6,6,6,14_P][Cl]	*L. monocytogenes* ATCC13932	5.7		Broth microdilution		[96]
		*B. cereus* ATCC 11778	9.77				
		*S. aureus* ATCC 6538	8.14				
		*E. faecalis* ATCC 19433	11.39				
		*L. sakei* ATCC 15521	8.14				
		*L. lactis* ATCC 19435	8.14				
		*S. typhimurium* ATCC 14028	625				
		*E. coli* ATCC 25922	5000				
		*C. freundii* ATCC 27853	5000				
Gentamycin		*S. typhimurium* ATCC 14028	0.25		Broth microdilution		[81]
		*E. coli* ATCC 25922	0.25				
		*C. freundii* ATCC 27853	1				
		*B. subtilis* KCTC1914	1				
		*S. typhimurium* KCTC1926	0.5				
Kanamycin		*S. aureus* 209 KCTC1916	2		Broth microdilution		[81]
		*S. aureus* R209 KCTC1928	1				
		*E. coli* KCTC1924	16				
		*B. subtilis* KCTC1914	2				
		*S. typhimurium* KCTC1926	1				
Fuconazole		*C. tropicalis* 17A	0.125 (MBEC 4)		Broth microdilution	The clinical isolates 72A, 72P, and 94P are resistant to fluconazole, amphotericin B, voriconazole and anidulafungin.	[88]
		*C. tropicalis* 57A	0.125 (MBEC 64)				
		*C. tropicalis* 72A	128 (MBEC 8)				
		*C. tropicalis* 72P	128 (MBEC 128)				
		*C. tropicalis* 94P	64 (MBEC 32)				
		*C. tropicalis* 102A	0.125 (MBEC 128)				
Colistin		*E. coli* ATCC 25922	2		Broth microdilution		[91]
		*P. aeruginosa* ATCC 27853	1				
		*K. pneumonia* ATCC BAA-1705	2				
		*A. baumannii* AB01	4				
Vancomycin		*B. subtilis* ATCC 6633	<0.48		Broth microdilution		[92]
		*K. pneumonia* ATCC 9997	15.62				
		*E. faecalis* ATCC 29212	1.95				
		*VRE* ATCC 51299	3.91				
		*S. aureus*	7.82				
		MRSA CIP 106760	3.91				
Rifampicin		*M. smegmatis* ATCC 607	<0.48		Broth microdilution		[92]
		*E. coli* ATCC 25922	0.98				
Norfloxacin		*P. aeruginosa* ATCC 27853	<0.48		Broth microdilution		[92]
Amphotericin B		*C. albicans* ATCC 10231	<0.48		Broth microdilution		[92]
		*S. cerevisiae* ATCC 2601	<0.48				

* IZ, inhibition zone; MIC, minimum inhibitory concentration; MBC, minimum bactericidal concentration; MBEC, minimum biofilm eradication concentration; MRSA, methicillin-resistant *S. aureus*; N.A., not active; N.T., not tested; VRE, vancomycin-resistant *E. faecalis*. ^a^ Anti-adhesive activity varies depending on the species.

**Table 3:** Antimicrobial activity of API-ILs *.

IL	Acronym	Species	IZ, mm	MIC μg mL^−1^	MBC, μg mL^−1^	Method	Notes	Ref.
1-Ethyl-3-methylimidazolium nalidixate	[C_2_Mim][Nal]	*E. coli* BW25113 (wild-type)	11			Disk diffusion test, 10 µg per disk	Deletions ΔrfaC, ΔrfaL, and ΔrfaG affect the cell surface hydrophobicity and membrane permeability.	[86]
		*E. coli* JW3596 (ΔrfaC)	20					
		*E. coli* JW3597 (ΔrfaL)	11					
		*E. coli* JW3606 (ΔrfaG)	18					
1-Hexadecyl-3-methylimidazolium ampicillinate	[C_16_Mim][Amp]	*S. aureus* ATCC 6538		30 µM		Broth microdilution		[97]
		*E. coli* O157:H7 ATCC 43895		9 µM				
		*E. faecium* ATCC 49474		13 µM				
		*K. pneumonia* ATCC 4352		15 µM				
1-Hexadecyl-2,3-dimethylimidazolium ampicillinate	[C_16_MMim][Amp]	*S. aureus* ATCC 6538		14 µM		Broth microdilution		[97]
		*E. coli* O157:H7 ATCC 43895		9 µM				
		*E. faecium* ATCC 49474		0.4 µM				
		*K. pneumonia* ATCC 4352		15 µM				
1-Hexadecylpyridinium ampicillinate	[C_16_Py][Amp]	*S. aureus* ATCC 6538		8 µM		Broth microdilution	*E. coli* TEM CTX M9, CTX M2, and AmpC MOX2 are ampicillin-resistant strains.	[82,97]
		*S. aureus* ATCC 25293		5 µM				
		*S. epidermidis* (clinical isolate)		5 µM				
		*E. coli* O157:H7 ATCC 43895		6 µM				
		*E. coli* ATCC 25922		500 µM				
		*E. coli* TEM CTX M9		5 µM				
		*E. coli* CTX M2		50 µM				
		*E. coli* AmpC MOX2		>5000 µM				
		*E. faecium* ATCC 49474		0.4 µM				
		*E. faecalis* (clinical isolate)		5 µM				
		*K. pneumonia* ATCC 4352		9 µM				
		*K. pneumoniae (clinical isolate)*		50 µM				
*N*-Ethyl-*N*-methylpiperidinium nalidixate	[C_2_C_1_Pip][Nal]	*E. coli* BW25113 (wild-type)	12.9			Disk diffusion test, 10 µg per disk	Deletions ΔrfaC, ΔrfaL, and ΔrfaG affect the cell surface hydrophobicity and membrane permeability.	[86]
		*E. coli* JW3596 (ΔrfaC)	22.9					
		*E. coli* JW3597 (ΔrfaL)	12.8					
		*E. coli* JW3606 (ΔrfaG)	21					
Trimethylhexadecylammonium nalidixate	[C_1,1,1,16_N][Nal]	*E. coli* BW25113 (wild-type)	12.6			Disk diffusion test, 10 µg per disk	Deletions ΔrfaC, ΔrfaL, and ΔrfaG affect the cell surface hydrophobicity and membrane permeability.	[86]
		*E. coli* JW3596 (ΔrfaC)	22.7					
		*E. coli* JW3597 (ΔrfaL)	12.2					
		*E. coli* JW3606 (ΔrfaG)	20.2					
Dioctyldimethylammonium nalidixate	[C_8,8,1,1_N][Nal]	*E. coli* BW25113 (wild-type)	13.3			Disk diffusion test, 10 µg per disk	Deletions ΔrfaC, ΔrfaL, and ΔrfaG affect the cell surface hydrophobicity and membrane permeability.	[86]
		*E. coli* JW3596 (ΔrfaC)	23.3					
		*E. coli* JW3597 (ΔrfaL)	13.6					
		*E. coli* JW3606 (ΔrfaG)	20.3					
Trioctylmethylammonium nalidixate	[C_8,8,8,1_N][Nal]	*E. coli* BW25113 (wild-type)	11.3			Disk diffusion test, 10 µg per disk	Deletions ΔrfaC, ΔrfaL, and ΔrfaG affect the cell surface hydrophobicity and membrane permeability.	[86]
		*E. coli* JW3596 (ΔrfaC)	22.2					
		*E. coli* JW3597 (ΔrfaL)	11					
		*E. coli* JW3606 (ΔrfaG)	18.7					
Tetramethylammonium nalidixate	[C_1,1,1,1_N][Nal]	*E. coli* BW25113 (wild-type)	13.3			Disk diffusion test, 10 µg per disk	Deletions ΔrfaC, ΔrfaL, and ΔrfaG affect the cell surface hydrophobicity and membrane permeability.	[86]
		*E. coli* JW3596 (ΔrfaC)	22.9					
		*E. coli* JW3597 (ΔrfaL)	13.4					
		*E. coli* JW3606 (ΔrfaG)	20.6					
Tetrabutylammonium nalidixate	[C_4,4,4,4_N][Nal]	*E. coli* BW25113 (wild-type)	13.3			Disk diffusion test, 10 µg per disk	Deletions ΔrfaC, ΔrfaL, and ΔrfaG affect the cell surface hydrophobicity and membrane permeability.	[86]
		*E. coli* JW3596 (ΔrfaC)	22.7					
		*E. coli* JW3597 (ΔrfaL)	13.6					
		*E. coli* JW3606 (ΔrfaG)	21.3					
Didecyldimethylammonium saccharinate	[C_10,10,1,1_N][Sac]	*S. aureus* ATCC 6538		4 ppm	62.5 ppm	Tube dilution		[99]
		*MRSA* ATCC 43300		4 ppm	31.2 ppm			
		*E. faecium* ATCC 49474		8 ppm	16 ppm			
		*E. coli* ATCC25922		16 ppm	16 ppm			
		*M. luteus* ATCC 9341		4 ppm	31.2 ppm			
		*S. epidermidis* ATCC 12228		4 ppm	16 ppm			
		*K. pneumonia* ATCC 4352		4 ppm	16 ppm			
		*C. albicans* ATCC 10231		16 ppm	16 ppm			
		*R. rubra* PhB		16 ppm	31.2 ppm			
		*S. mutans* PCM		31 ppm	62.5 ppm			
Didecyldimethylammonium acesulfamate	[C_10,10,1,1_N][Ace]	*S. aureus* ATCC 6538		8 ppm	16 ppm	Tube dilution		[99]
		*MRSA* ATCC 43300		4 ppm	31.2 ppm			
		*E. faecium* ATCC 49474		8 ppm	31.2 ppm			
		*E. coli* ATCC25922		16 ppm	62.5 ppm			
		*M. luteus* ATCC 9341		8 ppm	62.5 ppm			
		*S. epidermidis* ATCC 12228		4 ppm	31.2 ppm			
		*K. pneumonia* ATCC 4352		4 ppm	31.2 ppm			
		*C. albicans* ATCC 10231		16 ppm	31.2 ppm			
		*R. rubra* PhB		16 ppm	62.5 ppm			
		*S. mutans* PCM		16 ppm	125 ppm			
Tetrabutylphosphonium nalidixate	[C_4,4,4,4_P][Nal]	*E. coli* BW25113 (wild-type)	13.3			Disk diffusion test, 10 µg per disk	Deletions ΔrfaC, ΔrfaL, and ΔrfaG affect the cell surface hydrophobicity and membrane permeability.	[86]
		*E. coli* JW3596 (ΔrfaC)	22.6					
		*E. coli* JW3597 (ΔrfaL)	12.9					
		*E. coli* JW3606 (ΔrfaG)	20.4					
Trihexyltetradecylphosphonium ampicillinate	[C_6,6,6,14_P][Amp]	*E. coli* ATCC 25922		2500 µM		Broth microdilution	*E. coli* TEM CTX M9, CTX M2, and AmpC MOX2 are ampicillin-resistant strains.	[82]
		*E. coli* TEM CTX M9		500 µM				
		*E. coli* CTX M2		500 µM				
		*E. coli* AmpC MOX2		>5000 µM				
		*K. pneumoniae* (clinical isolate)		5000 µM				
		*S. aureus* ATCC 25293		50 µM				
		*S. epidermidis* (clinical isolate)		50 µM				
		*E. faecalis* (clinical isolate)		50 µM				
Benzalkonium saccharinate	[BA][Sac]	*S. aureus* ATCC 6538		4 ppm	31.2 ppm	Tube dilution		[99]
		*MRSA* ATCC 43300		4 ppm	31.2 ppm			
		*E. faecium* ATCC 49474		8 ppm	16 ppm			
		*E. coli* ATCC25922		16 ppm	62.5 ppm			
		*M. luteus* ATCC 9341		8 ppm	62.5 ppm			
		*S. epidermidis* ATCC 12228		4 ppm	31.2 ppm			
		*K. pneumonia* ATCC 4352		4 ppm	62.5 ppm			
		*C. albicans* ATCC 10231		16 ppm	31.2 ppm			
		*R. rubra* PhB		16 ppm	62.5 ppm			
		*S. mutans* PCM		0.1 ppm	0.5 ppm			
Benzalkonium acesulfamate	[BA][Ace]	*S. aureus* ATCC 6538		4 ppm	31.2 ppm	Tube dilution		[99]
		*MRSA* ATCC 43300		4 ppm	31.2 ppm			
		*E. faecium* ATCC 49474		8 ppm	31.2 ppm			
		*E. coli* ATCC25922		31 ppm	125 ppm			
		*M. luteus* ATCC 9341		8 ppm	62.5 ppm			
		*S. epidermidis* ATCC 12228		4 ppm	62.5 ppm			
		*K. pneumonia* ATCC 4352		8 ppm	31.2 ppm			
		*C. albicans* ATCC 10231		16 ppm	31.2 ppm			
		*R. rubra* PhB		16 ppm	62.5 ppm			
		*S. mutans* PCM		1 ppm	16 ppm			
Nalidixic acid		*E. coli* BW25113 (wild-type)	11			Disk diffusion test, 10 µg per disk	Deletions ΔrfaC, ΔrfaL, and ΔrfaG affect the cell surface hydrophobicity and membrane permeability.	[86]
		*E. coli* JW3596 (ΔrfaC)	20					
		*E. coli* JW3597 (ΔrfaL)	11					
		*E. coli* JW3606 (ΔrfaG)	18					
Ampicillin sodium salt		*S. aureus* ATCC 6538		27 µM		Broth microdilution	*E. coli* TEM CTX M9, CTX M2, and AmpC MOX2 are ampicillin-resistant strains.	[82,97]
		*S. aureus* ATCC 25293		5 µM				
		*S. epidermidis* (clinical isolate)		50 µM				
		*E. coli* O157:H7 ATCC 43895		12 µM				
		*E. coli* ATCC 25922		50 µM				
		*E. coli* TEM CTX M9		>5000 µM				
		*E. coli* CTX M2		>5000 µM				
		*E. coli* AmpC MOX2		>5000 µM				
		*E. faecium* ATCC 49474		17 µM				
		*E. faecalis* (clinical isolate)		50 µM				
		*K. pneumonia* ATCC 4352		20 µM				
		*K. pneumoniae* (clinical isolate)		2500 µM				
Benzalkonium chloride		*S. aureus* ATCC 6538		2 ppm	62.5 ppm	Tube dilution, broth microdilution		[81,99]
		*MRSA* ATCC 43300		2 ppm	31.2 ppm			
		*S. aureus* 209 KCTC1916		8				
		*S. aureus* R209 KCTC1928		8				
		*E. faecium* ATCC 49474		4 ppm	31.2 ppm			
		*E. coli* ATCC25922		8 ppm	62.5 ppm			
		*M. luteus* ATCC 9341		4 ppm	31.2 ppm			
		*S. epidermidis* ATCC 12228		2 ppm	16 ppm			
		*K. pneumonia* ATCC 4352		4 ppm	31.2 ppm			
		*B. subtilis* KCTC1914		8				
		*C. albicans* ATCC 10231		8 ppm	16 ppm			
		*R. rubra* PhB		8 ppm	31.2 ppm			
		*S. mutans* PCM		2 ppm	16 ppm			
Didecyldimethylammonium chloride		*S. aureus* ATCC 6538		2 ppm	31.2 ppm	Tube dilution		[99]
		*MRSA* ATCC 43300		2 ppm	31.2 ppm			
		*E. faecium* ATCC 49474		4 ppm	31.2 ppm			
		*E. coli* ATCC25922		8 ppm	31.2 ppm			
		*M. luteus* ATCC 9341		2 ppm	31.2 ppm			
		*S. epidermidis* ATCC 12228		2 ppm	31.2 ppm			
		*K. pneumonia* ATCC 4352		4 ppm	16 ppm			
		*C. albicans* ATCC 10231		8 ppm	16 ppm			
		*R. rubra* PhB		4 ppm	31.2 ppm			
		*S. mutans* PCM		2 ppm	16 ppm			

* IZ, inhibition zone; MIC, minimum inhibitory concentration; MBC, minimum bactericidal concentration; MRSA, methicillin-resistant *S. aureus*.

**Table 4:** Antimicrobial activity of Bis-QACs *.

Series/ Compound	Strain	MIC, mg⋅L^−1^	MBC, mg⋅L^−1^	Method	Notes	Ref.
**42**	*S. aureus* SH1000	1 μM		Broth microdilution		[75]
	*E. faecalis* OG1RF	1 μM				
	*E. coli* MC4100	2 μM				
	*P. aeruginosa* PAO1-WT	4 μM				
**43**	*S. aureus* SH1000	1 μM		Broth microdilution		[71]
	*E. faecalis* OG1RF	1 μM				
	*E. coli* MC4100	2 μM				
	*P. aeruginosa* PAO1-WT	4 μM				
**44**	*S. aureus* SH1000я	1 μM		Broth microdilution		[71]
	*E. faecalis* OG1RF	1 μM				
	*E. coli* MC4100	1 μM				
	*P. aeruginosa* PAO1-WT	4 μM				
**46**	*S. aureus* Mau 29/58	0.4 μM		Suspension micromethod		[101]
	*E. coli* 377/79	3.1 μM				
	*C. albicans* 45/54	1.5 μM				
**47**	*S. aureus*	13 μM		Broth microdilution		[103]
	*E. coli*	10 μM				
**48**	*S. aureus* SH1000	2	2	Broth microdilution		[105]
	*E. faecalis* OG1RF	18	18			
	*E. coli* MC4100	18	18			
	*P. aeruginosa* PAO1-WT	37	37			
**49**	*S. aureus* SH1000	10	10	Broth microdilution		[105]
	*E. faecalis* OG1RF	18	18			
	*E. coli* MC4100	37	37			
	*P. aeruginosa* PAO1-WT	149	149			
**50**	*S. aureus* SH1000	10	10	Broth microdilution		[105]
	*E. faecalis* OG1RF	30	30			
	*E. coli* MC4100	74	74			
	*P. aeruginosa* PAO1-WT	297	297			
**51**	*S. aureus* SH1000	4	4	Broth microdilution		[105]
	*E. faecalis* OG1RF	18	18			
	*E. coli* MC4100	37	37			
	*P. aeruginosa* PAO1-WT	74	74			
**52**	*S. aureus* SH1000	4	4	Broth microdilution		[105]
	*E. faecalis* OG1RF	10	10			
	*E. coli* MC4100	18	18			
	*P. aeruginosa* PAO1-WT	74	74			
**53**	*S. aureus* SH1000	0.5 μM		Broth microdilution		[107]
	MRSA 300-0114	1 μM				
	MRSA ATCC 33592	0.25 μM				
	*E. faecalis* OG1RF	0.25 μM				
	*E. coli* MC4100	1 μM				
	*P. aeruginosa* PAO1-WT	2 μM				
**54**	*S. aureus* ATCC 29213	0.5		Broth microdilution	Tested in vivo with proved efficiency	[106]
	*S. epidermidis* (clinical)	2				
	*B. subtilis* 168	1				
	*E. coli* ATCC 25922	0.5				
	*K. pneumoniae* 1813	4				
	*P. aeruginosa* ATCC 27853	0.5				
	*T. rubrum* 1336 (clinical)	32				
	*A. niger* F-1119	16				
	*C. albicans* NCTC- 885-653	16				
	*F. oxysporum* KM-19 (clinical)	32				
**55**	*S. aureus* ATCC 29213	4		Broth microdilution		[65]
**57**	*P. aeruginosa* ATCC 27583	6.3 μM		Broth microdilution		[108]
	*P. aeruginosa* ATCC 10145	5.2 μM				
	*P. aeruginosa* ATCC 3080	1.6 μM				
	*K. pneumoniae* ATCC 4352	0.4 μM				
	*K. pneumoniae* ATCC 13883	0.8 μM				
	*P. vulgaris* ATCC 13315	0.4 μM				
	*P. mirabilis* NBRC 3849	6.3 μM				
	*E. coli* K12 W3110	0.8 μM				
	*E. coli* IFO 3301	0.2 μM				
	*E. coli* IFO 3972	1.3 μM				
	*B. subtilis* IFO 3134	0.8 μM				
	*B. subtilis* ATCC 6633	0.8 μM				
	*B. cereus* IFO 3001	0.4 μM				
	*B. megaterium* IFO 3003	0.3 μM				
	*S. aureus* ATCC 25923	0.3 μM				
	*S. aureus* IFO 12732	0.4 μM				
	*A. niger* IFO 6341	8 μM				
	*A. niger* IFO 6342	4 μM				
	*A. niger* IFO 4414	4 μM				
	*C. globosum* IFO 6347	8 μM				
	*R. oryzae* IFO 31005	2 μM				
	*P. citrinum* IFO 6352	8 μM				
	*A. pullulans* IFO 6353	16 μM				
	*C. cladosporioides* IFO 6348	4 μM				
	*G. virens* IFO 6355	8 μM				
**58**	*S. aureus* SH1000	1 μM		Broth microdilution		[109]
	MRSA 300-0114	1 μM				
	MRSA ATCC 33592	2 μM				
	*E. faecalis* OG1RF	8 μM				
	*E. coli* MC4100	8 μM				
	*P. aeruginosa* PAO1-WT	8 μM				
**59**	*S. aureus* SH1000	0.25 μM		Broth microdilution		[109]
	MRSA 300-0114	2 μM				
	MRSA ATCC 33592	0.5 μM				
	*E. faecalis* OG1RF	4 μM				
	*E. coli* MC4100	2 μM				
	*P. aeruginosa* PAO1-WT	8 μM				
**60**	*S. aureus* ATCC 25923	64	128	Broth microdilution	Surfactant	[110]
	*B. subtilis* ATCC 6633	16	32			
	*E. coli* ATCC 25922	16	64			
**61**	*S. aureus* SH1000	1 μM		Broth microdilution	Natural derivatives	[74]
	MRSA 300-0114	4 μM				
	MRSA ATCC 33592	2 μM				
	*E. faecalis* OG1RF	2 μM				
	*E. coli* MC4100	4 μM				
	*P. aeruginosa* PAO1-WT	32 μM				
**62**	*S. aureus* SH1000	1 μM		Broth microdilution	Natural derivatives	[74]
	MRSA 300-0114	1 μM				
	MRSA ATCC 33592	1 μM				
	*E. faecalis* OG1RF	2 μM				
	*E. coli* MC4100	2 μM				
	*P. aeruginosa* PAO1-WT	8 μM				
**63**	*S. aureus* SH1000	2 μM		Broth microdilution		[111]
	MRSA 300-0114	1 μM				
	MRSA ATCC 33592	2 μM				
	*E. faecalis* OG1RF	4 μM				
	*E. coli* MC4100	1 μM				
	*P. aeruginosa* PAO1-WT	4 μM				
**64**	*S. aureus* SH1000	2 μM		Broth microdilution		[111]
	MRSA 300-0114	2 μM				
	MRSA ATCC 33592	2 μM				
	*E. faecalis* OG1RF	4 μM				
	*E. coli* MC4100	2 μM				
	*P. aeruginosa* PAO1-WT	4 μM				
**65**	*S. aureus* SH1000	0.5 μM		Broth microdilution		[112]
	MRSA 300-0114	0.5 μM				
	*E. coli* MC4100	1 μM				
	*P. aeruginosa* PAO1-WT	2 μM				
**66**	*S. aureus* SH1000	0.5 μM		Broth microdilution		[72]
	MRSA 300-0114	0.5 μM				
	MRSA ATCC 33592	0.5 μM				
**67**	*S. aureus* ATCC 29213	16		Broth microdilution		[113]
	*E. faecalis* ATCC 29212	64				
	*E. coli* ATCC 25922	128				
	*P. aeruginosa* ATCC 27853	256				
**68**	*S. aureus* ATCC 29213	0.25		Broth microdilution		[113]
	MRSA (*mecA*)	0.5				
	*E. faecalis* ATCC 29212	0.5				
	Vancomycin-resistant *E. faecalis* (*vanA*)	0.5				
	*E. coli* ATCC 25922	0.5				
	Extended-spectrum b-lactamase-producing *E. coli*	1				
	*P. aeruginosa* ATCC 27853	4				
	*P. aeruginosa* resistant, efflux pump	8				
**69**	*S. aureus* ATCC 29213	0.5		Broth microdilution		[113]
	MRSA (*mecA*)	0.5				
	*E. faecalis* ATCC 29212	0.5				
	Vancomycin-resistant *E. faecalis* (*vanA*)	0.5				
	*E. coli* ATCC 25922	0.5				
	Extended-spectrum b-lactamase-producing *E. coli*	1				
	*P. aeruginosa* ATCC 27853	2				
	*P. aeruginosa* resistant, efflux pump	2				
**70**	*P. aeruginosa* ATCC 27853	17 μM		Broth microdilution		[114]
	*K. pneumoniae* ATCC 4352	2.1 μM				
	*P. mirabilis* NBRC 3849	3.1 μM				
	*E. coli* IFO 12713	1.6 μM				
	*S. marcescens* ATCC 13880	3.1 μM				
	*M. luteus* IFO 12708	0.65 μM				
	*B. subtilis* ATCC 6633	0.91 μM				
	*B. cereus* IFO 3001	1.6 μM				
	*S. aureus* IFO 12732	0.23 μM				
	MRSA COL 1	1.6 μM				
**71**	*P. aeruginosa* ATCC 27853	13 μM		Broth microdilution		[114]
	*K. pneumoniae* ATCC 4352	1.6 μM				
	*P. mirabilis* NBRC 3849	5.2 μM				
	*E. coli* IFO 12713	1.6 μM				
	*S. marcescens* ATCC 13880	6.3 μM				
	*M. luteus* IFO 12708	0.78 μM				
	*B. subtilis* ATCC 6633	1.0 μM				
	*B. cereus* IFO 3001	1.3 μM				
	*S. aureus* IFO 12732	0.33 μM				
	MRSA COL 1	1.3 μM				
**72**	*S. aureus* ATCC 25923	4		Broth microdilution		[115]
	MRSA ATCC 33591	4				
	*E. faecalis* ATCC 1299	1				
	*E. coli* ATCC 25922	2				
	*P. aeruginosa* ATCC 27853	4				
	*K. pneumoniae* ATCC 13883	16				
	*A. flavus*	15.63				
	*C. albicans* 64124	3.91				
	*C. albicans* MYA2876	3.91				
	*C. neoformans*	3.9				
	*R. pilimanae*	2.0				
**73**	*S. aureus* SH1000	2 μM		Broth microdilution		[117]
	*E. faecalis* OG1RF	2 μM				
	*E. coli* MC4100	2 μM				
	*P. aeruginosa* PAO1-WT	16 μM				
**74**	*S. aureus* SH1000	0.5 μM		Broth microdilution		[117]
	*E. faecalis* OG1RF	0.5 μM				
	*E. coli* MC4100	0.5 μM				
	*P. aeruginosa* PAO1-WT	1 μM				
**75**	*S. aureus* SH1000	0.5 μM		Broth microdilution		[117]
	*E. faecalis* OG1RF	1 μM				
	*E. coli* MC4100	1 μM				
	*P. aeruginosa* PAO1-WT	2 μM				
**76**	*S. aureus* SH1000	1 μM		Broth microdilution		[118]
	MRSA 300-0114	1 μM				
	MRSA ATCC 33592	1 μM				
	*E. faecalis* OG1RF	4 μM				
	*E. coli* MC4100	1 μM				
	*P. aeruginosa* PAO1-WT	4 μM				
**77**	*S. aureus* SH1000	1 μM		Broth microdilution		[118]
	MRSA 300-0114	0.5 μM				
	MRSA ATCC 33592	2 μM				
	*E. faecalis* OG1RF	2 μM				
	*E. coli* MC4100	1 μM				
	*P. aeruginosa* PAO1-WT	2 μM				
**78**	*S. aureus* SH1000	16 μM		Broth microdilution		[118]
	MRSA 300-0114	32 μM				
	MRSA ATCC 33592	16 μM				
	*E. faecalis* OG1RF	63 μM				
	*E. coli* MC4100	32 μM				
	*P. aeruginosa* PAO1-WT	63 μM				
**79**	MRSA ATCC 43300	0.25		Broth microdilution		[119]
	*E. coli* ATCC 25922	4				
	*K. pneumoniae* ATCC 700603	16				
	*A. baumannii* ATCC 19606	4				
	*P. aeruginosa* ATCC 27853	8				
	*C. albicans* ATCC 90028	0.25				
	*C. neoformans* ATCC 208821	0.25				
**80**	MRSA ATCC 43300	0.25		Broth microdilution		[122,126]
	*E. coli* ATCC 25922	1				
	*K. pneumoniae* ATCC 700603	8				
	*A. baumannii* ATCC 19606	2				
	*P. aeruginosa* ATCC 27853	4				
	*C. albicans* ATCC 90028	0.25				
	*C. neoformans* ATCC 208821	0.25				
**81**	MRSA ATCC 43300	0.25		Broth microdilution		[123,126]
	*E. coli* ATCC 25922	0.25				
	*K. pneumoniae* ATCC 700603	0.25				
	*A. baumannii* ATCC 19606	0.25				
	*P. aeruginosa* ATCC 27853	0.25				
	*C. albicans* ATCC 90028	0.25				
	*C. neoformans* ATCC 208821	4				
**82**	MRSA ATCC 43300	0.25		Broth microdilution		[124]
	*E. coli* ATCC 25922	0.25				
	*K. pneumoniae* ATCC 700603	16				
	*A. baumannii* ATCC 19606	0.25				
	*P. aeruginosa* ATCC 27853	0.25				
	*C. albicans* ATCC 90028	0.25				
	*C. neoformans* ATCC 208821	0.25				
**83**	MRSA ATCC 43300	0.25		Broth microdilution		[125]
	*E. coli* ATCC 25922	0.25				
	*K. pneumoniae* ATCC 700603	0.25				
	*A. baumannii* ATCC 19606	8				
	*P. aeruginosa* ATCC 27853	0.25				
	*C. albicans* ATCC 90028	0.25				
	*C. neoformans* ATCC 208821	0.25				
**84**	*P. aeruginosa* ATCC 27583		6.3 μM	Broth microdilution		[127]
	*K. pneumoniae* ATCC 13883		3.1 μM			
	*P. mirabilis* IFO 3849		6.3 μM			
	*E. coli* K12 W3110		3.1 μM			
	*M. luteus* IFO 12708		0.78 μM			
	*B. cereus* IFO 3001		3.1 μM			
	*S. aureus* IFO 12732		0.39 μM			
	MRSA IID 1677		3.1 μM			
	*P. funiculosam* IFO 6345	1.6 μM				
	*C. globosum* IFO 6347	3.1 μM				
	*A. pullulans* IFO 6353	6.3 μM				
	*R. stolonifera* IFO 4781	25 μM				
	*A. terreus* IFO 6346	25 μM				
	*A. niger* IFO 6342	12.5 μM				
**85**	*E. coli*	2.7		Broth microdilution		[134]
**86**	*P. aeruginosa* ATCC 27583		13 μM	Broth microdilution		[127]
	*K. pneumoniae* ATCC 13883		1.6 μM			
	*P. mirabilis* IFO 3849		13 μM			
	*E. coli* K12 W3110		6.3 μM			
	*M. luteus* IFO 12708		0.39 μM			
	*B. cereus* IFO 3001		1.6 μM			
	*S. aureus* IFO 12732		0.39 μM			
	MRSA IID 1677		6.3 μM			
	*P. funiculosam* IFO 6345	1.6 μM				
	*C. globosum* IFO 6347	0.78 μM				
	*A. pullulans* IFO 6353	6.3 μM				
	*R. stolonifera* IFO 4781	25 μM				
	*A. terreus* IFO 6346	12.5 μM				
	*A. niger* IFO 6342	6.3 μM				
**87**	*P. aeruginosa* ATCC 27583		25 μM	Broth microdilution		[132]
	*K. pneumoniae* ATCC 13883		1.6 μM			
	*P. mirabilis* IFO 3849		13 μM			
	*E. coli* K12 W3110		6.3 μM			
	*M. luteus* IFO 12708		0.78 μM			
	*B. cereus* IFO 3001		3.1 μM			
	*S. aureus* IFO 12732		0.39 μM			
	MRSA IID 1677		6.3 μM			
	*P. funiculosum* IFO 6345	0.78 μM				
	*C. globosum* IFO 6347	0.78 μM				
	*A. pullulans* IFO 6353	3.1 μM				
	*R. stolonifera* IFO 4781	6.3 μM				
	*A. terreus* IFO 6346	1.6 μM				
	*A. niger* IFO 6342	6.3 μM				
**88**	*P. aeruginosa* ATCC 27583	6.3 μM		Broth microdilution		[129]
	*P. aeruginosa* ATCC 10145	8.3 μM				
	*K. pneumoniae* ATCC 4352	1.0 μM				
	*P. rettgeri* NIH 96	2.1 μM				
	*P. mirabilis* IFO 3849	25 μM				
	*E. coli* IFO 12713	1.8 μM				
	*S. enteritidis* IFO 3313	1.3 μM				
	*B. subtilis* IFO 3134	0.57 μM				
	*B. subtilis* ATCC 6633	1.0 μM				
	*B. cereus* IFO 3001	3.1 μM				
	*S. aureus* IFO 12732	0.46 μM				
	MRSA IID 1677	1.1 μM				
	*M. luteus* IFO 12708	0.26 μM				
	*A. niger* IFO 6342	25 μM				
	*A. niger* TSY 0013	13 μM				
	*A. pullulans* IFO 6353	3.1 μM				
	*P. citrinum* IFO 6345	25 μM				
	*P. funiculosum* IFO 6345	8.3 μM				
	*R. oryzae* IFO 31005	13 μM				
	*T. viride* IFO 30498	25 μM				
	*C. albicans* IFO 1061	29 μM				
**89**	*C. neoformans* ATCC 90112	1.3 μM		Broth microdilution		[133]
	*C. albicans* ATCC 10231	1.3 μM				
	*A. fumigatus* ATCC 204305	88 μM				
**90**	*E. coli* ATCC 25922	8	18	Broth microdilution		[120]
	*P. aeruginosa* ATCC 6538	32	8.3			
	*S. aureus* ATCC 278530	2.3	8.3			
	*A. baumannii* JCM 6841	11				
	*B. cepacia* JCM 5964	19				
	*E. hirae* ATCC 10541	5.3				
	*E. faecalis* ATCC 29212	6.7				
	MRSA ATCC 700698	11				
	*S. epidermidis* ATCC 12228	5.3				
	*C. albicans* ATCC 10231	13				
**91**	*E. coli* ATCC 25922	1.7	15	Broth microdilution		[120]
	*P. aeruginosa* ATCC 6538	21	8.3			
	*S. aureus* ATCC 278530	1.7	33			
	*A. baumannii* JCM 6841	16				
	*B. cepacia* JCM 5964	64				
	*E. hirae* ATCC 10541	16				
	*E. faecalis* ATCC 29212	19				
	MRSA ATCC 700698	8				
	*S. epidermidis* ATCC 12228	9.3				
	*C. albicans* ATCC 10231	27				
**92**	MRSA ATCC 25923	2 ppm		Broth microdilution		[135]
	*E. coli* ATCC 25922	4 pmm				
	*P. aeruginosa* ATCC 27853	16 ppm				

* MIC, minimum inhibitory concentration; MBC, minimum bactericidal concentration; MRSA, methicillin-resistant *S. aureus*; only leader compounds from the series are listed in the table.

**Table 5:** Antimicrobial activity of dicationic ILs *.

IL	Acronym	Species	IZ, mm	MIC, μg mL−1	MBC, μg mL−1	Method	Ref.
2-Methyl-3-(4-(2-methyl-5-nitro-1H-imidazolium bromide)butyl-5-nitro-1H-imidazolium bromide	([NO_2_C_1_Im]-C_4_-[NO_2_C_1_Im])[Br]_2_	*S. aureus*	16	0.25	0.25	Disk diffusion (100 µg per well); broth microdilution	[139]
		*E. coli*	15	0.25	0.25		
		*K. pneumoniae*	16	0.255	0.255		
		*P. aeruginosa*	14	0.255	0.255		
		*P. vulgaris*	15	0.27	0.27		
2-Methyl-3-(4-(2-methyl-5-nitro-1H-imidazolium tetrafluoroborate)butyl-5-nitro-1H-imidazolium tetrafluoroborate	([NO_2_C_1_Im]-C_4_-[NO_2_C_1_Im])[BF_4_]_2_	*S. aureus*	15	0.27	0.27	Disk diffusion (100 µg per well); broth microdilution	[139]
		*E. coli*	16	0.27	0.27		
		*K. pneumoniae*	12	0.27	0.27		
		*P. aeruginosa*	12	0.27	0.27		
		*P. vulgaris*	14	0.27	0.27		
2-Methyl-3-(4-(2-methyl-5-nitro-1H-imidazolium hexafluorophosphate)butyl-5-nitro-1H-imidazolium hexafluorophosphate	([NO_2_C_1_Im]-C_4_-[NO_2_C_1_Im])[PF_6_]_2_	*S. aureus*	16.5	0.255	0.255	Disk diffusion (100 µg per well); broth microdilution	[139]
		*E. coli*	16	0.255	0.255		
		*K. pneumoniae*	15.5	0.255	0.255		
		*P. aeruginosa*	15	0.27	0.27		
		*P. vulgaris*	16	0.27	0.27		
2-Methyl-3-(4-(2-methyl-5-nitro-1H-imidazolium trifluoromethanesulfonate)butyl-5-nitro-1H-imidazolium trifluoromethanesulfonate	([NO_2_C_1_Im]-C_4_-[NO_2_C_1_Im])[TfO]_2_	*S. aureus*	16	0.27	0.27	Disk diffusion (100 µg per well); broth microdilution	[139]
		*E. coli*	14	0.255	0.255		
		*K. pneumoniae*	14	0.27	0.27		
		*P. aeruginosa*	13	0.27	0.27		
		*P. vulgaris*	15	0.27	0.27		
Erythromycin		*S. aureus*	24	0.23	0.23	Disk diffusion (30 µg per well); broth microdilution	[139]
		*E. coli*	27	0.23	0.23		
		*K. pneumoniae*	26	0.23	0.23		
		*P. aeruginosa*	25	0.23	0.23		
		*P. vulgaris*	32	0.23	0.23		
Nalidixic acid		*S. aureus*	22	0.23	0.23	Disk diffusion (30 µg per well); broth microdilution	[139]
		*E. coli*	22	0.23	0.23		
		*K. pneumoniae*	27	0.23	0.23		
		*P. aeruginosa*	21	0.23	0.23		
		*P. vulgaris*	24	0.23	0.23		
Amikacin		*S. aureus*	19	0.23	0.23	Disk diffusion (30 µg per well); broth microdilution	[139]
		*E. coli*	20	0.23	0.23		
		*K. pneumoniae*	19	0.23	0.23		
		*P. aeruginosa*	17	0.23	0.23		
		*P. vulgaris*	17	0.23	0.23		

* IZ, inhibition zone; MIC, minimum inhibitory concentration; MBC, minimum bactericidal concentration.

**Table 6:** Antimicrobial activity of multi-QACs *.

Series/ Compound	Strain	MIC, mg⋅L^−1^	Method	Notes	Ref.
**93**	*S. aureus* SH1000	1 μM	Broth microdilution		[71]
	*E. faecalis* OG1RF	1 μM			
	*E. coli* MC4100	1 μM			
	*P. aeruginosa* PAO1-WT	2 μM			
**94**	*S. aureus* SH1000	0.5 μM	Broth microdilution		[71]
	*E. faecalis* OG1RF	1 μM			
	*E. coli* MC4100	1 μM			
	*P. aeruginosa* PAO1-WT	4 μM			
**95**	*S. aureus* SH1000	1 μM	Broth microdilution		[112]
	MRSA 300-0114	0.5 μM			
	MRSA ATCC 33592	1 μM			
**96**	*S. aureus* SH1000	1 μM	Broth microdilution		[72]
	MRSA 300-0114	1 μM			
	*E. coli* MC4100	2 μM			
	*P. aeruginosa* PAO1-WT	4 μM			
**96**	*S. aureus* SH1000	0.5 μM	Broth microdilution		[140]
	MRSA 300-0114	0.5 μM			
	MRSA ATCC 33592	0.5 μM			
	*E. faecalis* OG1RF	1 μM			
	*E. coli* MC4100	0.5 μM			
	*P. aeruginosa* PAO1-WT	0.5 μM			
**98**	*S. aureus* SH1000	1 μM	Broth microdilution		[107]
	MRSA 300-0114	0.5 μM			
	MRSA ATCC 33592	0.5 μM			
	*E. faecalis* OG1RF	1 μM			
	*E. coli* MC4100	0.5 μM			
	*P. aeruginosa* PAO1-WT	4 μM			
**99**	*B. cereus*	2 μM	Broth microdilution		[141]
	*E. faecalis* ATCC 29212	2 μM			
	*S. agalactiae* J48	2 μM			
	*S. aureus* ATCC 29213	2 μM			
	*E. coli* ATCC 25922	4 μM			
	*P. aeruginosa* ATCC 27853	16 μM			
**100**	*S. aureus* SH1000	0.5 μM	Broth microdilution		[143]
	*E. faecalis* OG1RF	1 μM			
	*E. coli* MC4100	1 μM			
	*P. aeruginosa* PAO1-WT	2 μM			
	MRSA 300-0114	0.5 μM			
	MRSA ATCC 33592	0.5 μM			
**101**	MRSA ATCC 25923	4	Broth microdilution	The first tetra-pyridinic salts	[144]
	*E. coli* ATCC 25922	4			
	*P. aeruginosa* ATCC 27853	32			

* MIC, minimum inhibitory concentration; MBC, minimum bactericidal concentration; MRSA, methicillin-resistant *S. aureus*; only leader compounds from the series are listed in the table.

**Table 7:** Antimicrobial activity of poly-QACs *.

Series/ Compound	Strain	MIC, mg⋅L^−1^	MBC, mg⋅L^−1^	Method	Notes	Ref.
**102**	*E. coli* ATCC 25922		1.56	Broth microdilution		[148]
	*S. aureus* ATCC 25923		1.56			
**103**	*E. coli* ATCC 8099	0.78		Broth microdilution		[149]
	*S. aureus* ATCC 6538	0.91				
**104**	*E. coli* ATCC 8099	0.13		Broth microdilution		[149]
	*S. aureus* ATCC 6538	0.28				
**105**	*E. coli* ATCC 25922	7		Broth tube dilution		[153]
	*S. aureus* ATCC 6538 P	7				
	*C. albicans* ATCC 865-653	3.5				
	*P. aeruginosa* ATCC 9027	31				
	*P. mirabilis* 47	31				
	*K. pneumoniae* ATCC 13883	62				
**106**	*E. coli*	62.5	62.5	Broth dilution		[152]
	*S. aureus*	62.5	62.5			
**107**	*E. coli*	22 mm/mg (IZ)		Disk diffusion	Possesses anticorrosion activity	[151]
	*S. aureus*	20 mm/mg (IZ)				
	*C. albicans*	13 mm/mg (IZ)				
	*P. aeruginosa*	24 mm/mg (IZ)				
	*A. niger*	12 mm/mg (IZ)				
**108**	*B. cinerea*	106		Radial growth technique	Efficient against fungal spores	[150]
	*F. oxysporum*	720				
	*P. debaryanum*	164				
**109**	*S. aureus*	5.3 (log reduction, 24 h contact)		Plate count	Prevent biofouling	[155]
	*P. aeruginosa*	5.4 (log reduction, 24 h contact)				
**110**	*S. aureus*	1.7 (log reduction, 24 h contact)		Plate count		[155]
	*P. aeruginosa*	1.9 (log reduction, 24 h contact)				
**111**	*S. aureus*	6 (log reduction, 24 h contact)		Plate count		[154]
	*E. coli*	6 (log reduction, 24 h contact)				
	*P. aeruginosa*	4.5 (log reduction, 24 h contact)				
**112**	*S. aureus*	128		Plate count		[156]
	*E. coli*	256				
**113**	*S. aureus* ATCC 6538P	7.26 (log reduction, 1 min contact)		Plate count		[160]
	*E. coli* ATCC 1122	8.26 (log reduction, 1 min contact)				

* IZ, inhibition zone; MIC, minimum inhibitory concentration; MBC, minimum bactericidal concentration; MRSA, methicillin-resistant *S. aureus*; only leader compounds from the series are listed in the table.

**Table 8:** Antimicrobial activity of poly-ILs *.

Series/ Compound	IL	Species	MIC, μM	MBC, μM	Method	Notes	Ref.
**103**	Poly-(vinylbenzyl dimethylhexylammonium chloride)	*S. aureus* ATCC 6538	910		Broth microdilution	Side-chain polymer	[149]
		*E. coli* ATCC 8099	780				
**104**	Poly-((*N*,*N*-dimethyl-N-(4-((trimethylammonio)methyl)benzyl)hexan-1-aminium) dibromide)	*S. aureus* ATCC 6538	280		Broth microdilution	Main-chain polymer	[149]
		*E. coli* ATCC 8099	130				
**114**	3-(2-(Methacryloyloxy)ethyl)-1-hexylimidazolium bromide-based polymer	*E. coli* ATCC 25922		3.62	Shake flask test	Antibacterial coating	[162]
**115**	3-(2-(Methacryloyloxy)ethyl)-1-octylimidazolium bromide-based polymer	*E. coli* ATCC 25922		1.67	Shake flask test	Antibacterial coating	[162]
**116**	3-(2-(Methacryloyloxy)ethyl)-1-dodecylimidazolium bromide-based polymer	*E. coli* ATCC 25922		<0.46	Shake flask test	Antibacterial coating	[162]
**117**	Poly(1-ethyl-3-vinylimidazolium bromide)	*S. aureus* ATCC 6538	110345		Broth microdilution		[164]
		*E. coli* ATCC 8099	110345				
**118**	Poly(1-butyl-3-vinylimidazolium bromide)	*S. aureus* ATCC 6538	2961		Broth microdilution		[164]
		*E. coli* ATCC 8099	5922				
**119**	Poly(1-octyl-3-vinylimidazolium bromide)	*S. aureus* ATCC 6538	1491 (3.71 for NPs)		Broth microdilution		[164,170]
		*E. coli* ATCC 8099	1192 (1.85 for NPs)				
**120**	Poly(1-decyl-3-vinylimidazolium bromide)	*S. aureus* ATCC 6538	3.57		Broth microdilution	NPs	[170]
		*E. coli* ATCC 8099	1.84				
**121**	Poly(1-dodecyl-3-vinylimidazolium bromide)	*S. aureus* ATCC 6538	61 (2.52 for NPs)		Broth microdilution		[164,170]
		*E. coli* ATCC 8099	122 (1.19 for NPs)				
**122**	Poly(1-hexadecyl-3-vinylimidazolium bromide)	*S. aureus* ATCC 6538	3.15		Broth microdilution	NPs	[170]
		*E. coli* ATCC 8099	2.72				
**123**	Poly(1-ethyl-3-(1-vinylimidazolium-3-hexyl)imidazolium bromide)	*S. aureus* ATCC 6538	33180		Broth microdilution		[164]
		*E. coli* ATCC 8099	33180				
**124**	Poly(1-butyl-3-(1-vinylimidazolium-3-hexyl)imidazolium bromide)	*S. aureus* ATCC 6538	918		Broth microdilution		[164]
		*E. coli* ATCC 8099	1853				
**125**	Poly(1-octyl-3-(1-vinylimidazolium-3-hexyl)imidazolium bromide)	*S. aureus* ATCC 6538	81		Broth microdilution		[164]
		*E. coli* ATCC 8099	41				
**126**	Poly(1-dodecyl-3-(1-vinylimidazolium-3-hexyl)imidazolium bromide)	*S. aureus* ATCC 6538	9		Broth microdilution		[164]
		*E. coli* ATCC 8099	18				
**127**	Poly-(*N*-Butyl-*N*-methylpyrrolidinonium bromide)	*S. aureus*	549		Broth microdilution		[89]
		*E. coli*	2196				
**128**	Poly-(*N*-Hexyl-*N*-methylpyrrolidinonium bromide)	*S. aureus*	236		Broth microdilution		[89]
		*E. coli*	548				
**129**	Poly-(*N*-Octyl-*N*-methylpyrrolidinonium bromide)	*S. aureus*	147		Broth microdilution		[89]
		*E. coli*	424				
**130**	Poly-(*N*-Decyl-*N*-methylpyrrolidinonium bromide)	*S. aureus*	112		Broth microdilution		[89]
		*E. coli*	224				
**131**	Poly-(*N*-Dodecyl-*N*-methylpyrrolidinonium bromide)	*S. aureus*	61		Broth microdilution		[89]
		*E. coli*	90				
**132**	Poly-(1-vinylbenzyl-3-hexylimidazolium chloride)	*S. aureus* ATCC 6538	900		Broth microdilution	Side-chain polymer	[149]
		*E. coli* ATCC 8099	770				
**133**	Poly-(1-vinylbenzyl-4-hexyl-1,4-diazoniabicyclo[2 .2.2]octane-1,4-diium chloride bromide)	*S. aureus* ATCC 6538	1280		Broth microdilution	Side-chain polymer	[149]
		*E. coli* ATCC 8099	1160				
**134**	Poly-(1-hexyl-3-methylimidazolium bromide)	*S. aureus* ATCC 6538	230		Broth microdilution	Main-chain polymer	[149]
		*E. coli* ATCC 8099	110				
**135**	Poly-(1-hexyl-4-methyl-1,4-diazoniabicyclo[2.2.2]octane-1,4-diium dibromide)	*S. aureus* ATCC 6538	560		Broth microdilution	Main-chain polymer	[149]
		*E. coli* ATCC 8099	510				

* IZ, inhibition zone; MIC, minimum inhibitory concentration; MBC, minimum bactericidal concentration; MBEC, minimum biofilm eradication concentration; MRSA, methicillin-resistant *S. aureus*; NPs, nanoparticles.

## Supplementary Materials

The Supplementary Materials are available online at https://www.mdpi.com/article/10.3390/ijms22136793/s1.
