# Supplementary material for: Quaternary Ammonium Compounds (QACs) and Ionic Liquids (ILs) as Biocides: From Simple Antiseptics to Tunable Antimicrobials
Source: Int J Mol Sci. 2021 Jun 24;22(13):6793. doi: 10.3390/ijms22136793 (PMC8268321; doi:10.3390/ijms22136793)
Supplement: Supplementary file 1 [file ijms-22-06793-s001.zip › ijms-1248861-supplementary.pdf]

Quaternary Ammonium Compounds (QACs) and Ionic Liquids (ILs) as Biocides: From Simple Antiseptics to Tunable Antimicrobials

Anatoly N. Vereshchagin[a], Nikita A. Frolov[a], Ksenia S. Egorova[a], Marina M. Seitkalieva[a], Valentine P. Ananikov[a]

[a] *N. D. Zelinsky Institute of Organic Chemistry, Russian Academy of Sciences, Leninsky prospect 47, Moscow 119991, Russia; vereshchagin@ioc.ac.ru (A. N. V.); nikitafrolov298@gmail.com (N. A. F.); egorova-ks@ioc.ac.ru (K. S. E.); s_marina@ioc.ac.ru (M. M. S.); val@ioc.ac.ru (V. P. A.).*

Table S1. Antimicrobial activity of common ILs*

IL	Acronym	Species	MIC, µg mL−1	MBC, µg mL−1	Method	Notes	Ref.
1-Ethyl-3-methylimidazolium chloride	[C_2Mim][Cl]	*L. monocytogenes* ATCC13932	>5000		Broth microdilution		[1]
		B. cereus ATCC 11778	>5000				
		S. aureus ATCC 6538	>5000				
		E. faecalis ATCC 19433	>5000				
		L. sakei ATCC 15521	>5000				
		L. lactis ATCC 19435	>5000				
		S. typhimurium ATCC 14028	>5000				
		E. coli ATCC 25922	>5000				
		C. freundii ATCC 27853	>5000				
1-(2-Hydroxyethyl)-3-methylimidazolium chloride	[HOC_2Mim][Cl]	*E. coli* ATCC 25922	5000 µM		Broth microdilution	*E. coli* TEM CTX M9, CTX M2, and AmpC MOX2 are ampicillin-resistant strains.	[2]
		E. coli TEM CTX M9	>5000 µM				
		E. coli CTX M2	2500 µM				
		E. coli AmpC MOX2	5000 µM				
		K. pneumoniae (clinical isolate)	>5000 µM				
		S. aureus ATCC 25293	>5000 µM				
		S. epidermidis (clinical isolate)	5000 µM				
		E. faecalis (clinical isolate)	5000 µM				
1-Butyl-3-methylimidazolium chloride	[C_4Mim][Cl]	*P. aeruginosa* PTCC1310	12500	12500	Agar disk diffusion / agar well diffusion, broth microdilution	Weak anti-adhesive activity[a]	[1, 3]
		S. aureus PTCC1112	50000	50000			
		S. aureus ATCC 6538	>5000				
		E. coli PTCC 1338	12500	12500			
		E. coli ATCC 25922	>5000				
		B. cereus PTCC 1015	50000	50000			

		B. cereus ATCC 11778	>5000				
		S. typhimurium (wild type)	12500	50000			
		S. typhimurium ATCC 14028	>5000				
		K. pneumonia PTCC 1290	25000	N.D.			
		B. subtilis PTCC 1715	50000	N.D.			
		L. monocytogenes ATCC 13932	>5000				
		E. faecalis ATCC 19433	>5000				
		L. sakei ATCC 15521	4167				
		L. lactis ATCC 19435	3333				
		C. freundii ATCC 27853	>5000				
1-Butyl-3-methylimidazolium iodide	$[C_4Mim][I]$	*P. aeruginosa* PTCC 1310	12500	12500	Agar disk diffusion / agar well diffusion	Weak anti-adhesive activity[a]	[3]
		S. aureus PTCC 1112	12500	25000			
		E. coli PTCC 1338	25000	25000			
		B. cereus PTCC 1015	50000	50000			
		S. typhimurium (wild type)	12500	25000			
		K. pneumonia PTCC 1290	25000	25000			
		B. subtilis PTCC 1715	25000	25000			
1-Butyl-3-methylimidazolium hexafluorophosphate	$[C_4Mim][PF_6]$	*P. aeruginosa* PTCC 1310	25000	50000	Agar disk diffusion / agar well diffusion		[3]
		S. aureus PTCC 1112	25000	25000			
		E. coli PTCC 1338	12500	12500			
		B. cereus PTCC 1015	50000	50000			
		S. typhimurium (wild type)	50000	50000			
		K. pneumonia PTCC 1290	25000	25000			
		B. subtilis PTCC 1715	50000	50000			
1-Butyl-3-methylimidazolium tetrafluoroborate	$[C_4Mim][BF_4]$	*P. aeruginosa* PTCC 1310	12500	50000	Agar disk diffusion / agar well diffusion		[3]
		S. aureus PTCC 1112	12500	12500			
		E. coli PTCC 1338	12500	12500			
		B. cereus PTCC 1015	50000	50000			
		S. typhimurium (wild type)	12500	12500			
		K. pneumonia PTCC 1290	25000	N.D.			
		B. subtilis PTCC 1715	50000	N.D.			

1-Butyl-3-methylimidazolium hydrogen sulfate	$[C_4Mim][HSO_4]$	*P. aeruginosa* PTCC 1310	6250	12500	Agar disk diffusion / agar well diffusion	Anti-adhesive activity[a]	[3]
		S. aureus PTCC 1112	3120	3120			
		E. coli PTCC 1338	3120	3120			
		B. cereus PTCC 1015	3120	3120			
		S. typhimurium (wild type)	6250	6250			
		K. pneumonia PTCC 1290	3120	6250			
		B. subtilis PTCC 1715	3120	3120			
1-Butyl-3-methylimidazolium methyl sulfate	$[C_4Mim][C_1OSO_3]$	*P. aeruginosa* PTCC 1310	12500	12500	Agar disk diffusion / agar well diffusion	Anti-adhesive activity[a]	[3]
		S. aureus PTCC 1112	12500	12500			
		E. coli PTCC 1338	12500	12500			
		B. cereus PTCC 1015	12500	12500			
		S. typhimurium (wild type)	12500	12500			
		K. pneumonia PTCC 1290	12500	50000			
		B. subtilis PTCC 1715	12500	12500			
1-Butyl-3-methylimidazolium tosylate	$[C_4Mim][PTS]$	*P. aeruginosa* PTCC 1310	25000	50000	Agar disk diffusion / agar well diffusion	Anti-adhesive activity[a]	[3]
		S. aureus PTCC 1112	50000	50000			
		E. coli PTCC 1338	25000	25000			
		B. cereus PTCC 1015	50000	50000			
		S. typhimurium (wild type)	25000	25000			
		K. pneumonia PTCC 1290	25000	25000			
		B. subtilis PTCC 1715	25000	25000			
1-Butyl-3-methylimidazolium nitrate	$[C_4Mim][NO_3]$	*P. aeruginosa* PTCC 1310	12500	50000	Agar disk diffusion / agar well diffusion	Anti-adhesive activity[a]	[3]
		S. aureus PTCC 1112	12500	12500			
		E. coli PTCC 1338	12500	12500			
		B. cereus PTCC 1015	50000	50000			
		S. typhimurium (wild type)	12500	12500			
		K. pneumonia PTCC 1290	25000	25000			
		B. subtilis PTCC 1715	50000	50000			
1-Butyl-3-methylimidazolium dicyanamide	$[C_4Mim][N(CN)_2]$	*P. aeruginosa* PTCC 1310	25000	25000	Agar disk diffusion / agar well diffusion	Anti-adhesive activity[a]	[3]
		S. aureus PTCC 1112	25000	25000			
		E. coli PTCC 1338	25000	25000			
		B. cereus PTCC 1015	25000	25000			
		S. typhimurium (wild type)	25000	25000			
		K. pneumonia PTCC 1290	25000	25000			

		B. subtilis PTCC 1715	25000	25000			
1-Butyl-3-methylimidazolium thiocyanate	[C_4Mim][SCN]	*P. aeruginosa* PTCC 1310	6250	6250	Agar disk diffusion / agar well diffusion	Anti-adhesive activity[a]	[3]
		S. aureus PTCC 1112	3120	3120			
		E. coli PTCC 1338	6250	6250			
		B. cereus PTCC 1015	25000	25000			
		S. typhimurium (wild type)	6250	12500			
		K. pneumonia PTCC 1290	6250	6250			
		B. subtilis PTCC 1715	25000	25000			
1-Hexyl-3-methylimidazolium chloride	[C_6Mim][Cl]	*S. aureus*	1560	1560	Agar disk diffusion / agar well diffusion, broth microdilution	Anti-adhesive activity[a]	[4, 5]
		S. aureus ATCC 29213	>1644 µM	>1644 µM			
		E-MRSA 15	>1644 µM	>1644 µM			
		MRSA (clinical strain 201)	>1644 µM	>1644 µM			
		S. aureus ATCC 6538	833				
		K. pneumoniae	3120	3120			
		S. typhimurium	6250	6250			
		S. typhimurium ATCC 14028	5000				
		P. aeruginosa	6250	12500			
		P. aeruginosa PA01	>1644 µM	>1644 µM			
		E. coli	3120	3120			
		E. coli ATCC 27325	>1644 µM	>1644 µM			
		E. coli ATCC 25922	1250				
		B. tequilensis	6250	6250			
		B. subtilis	6250	12500			
		S. epidermidis ATCC 12228	>1644 µM	>1644 µM			
		S. epidermidis ATCC 35984	>1644 µM	>1644 µM			
		K. aerogenes NCTC 7427	>1644 µM	>1644 µM			
		B. cenocepacia J2315	>1644 µM	>1644 µM			
		P. mirabilis NCTC 12442	>1644 µM	>1644 µM			

		C. tropicalis NCTC 7393	>1644 µM	>1644 µM			
		L. monocytogenes ATCC13932	3750				
		B. cereus ATCC 11778	5000				
		E. faecalis ATCC 19433	4167				
		L. sakei ATCC 15521	208				
		L. lactis ATCC 19435	625				
		C. freundii ATCC 27853	3333				
1-Hexyl-3-methylimidazolium iodide	$[C_6Mim][I]$	*S. aureus*	780	780	Agar disk diffusion / agar well diffusion	Anti-adhesive activity[a]	[4]
		K. pneumoniae	6250	6250			
		S. typhimurium	6250	12500			
		P. aeruginosa	1560	1560			
		E. coli	1560	6250			
		B. tequilensis	12500	12500			
		B. subtilis	3120	3120			
1-Hexyl-3-methylimidazolium hexafluorophosphate	$[C_6Mim][PF_6]$	*S. aureus*	3120	3120	Agar disk diffusion / agar well diffusion	Anti-adhesive activity[a]	[4]
		K. pneumoniae	25000	25000			
		S. typhimurium	25000	25000			
		P. aeruginosa	25000	25000			
		E. coli	12500	12500			
		B. tequilensis	25000	25000			
		B. subtilis	50000	50000			
1-Hexyl-3-methylimidazolium tetrafluoroborate	$[C_6Mim][BF_4]$	*S. aureus*	1560	1560	Agar disk diffusion / agar well diffusion	Anti-adhesive activity[a]	[4]
		K. pneumoniae	3120	6250			
		S. typhimurium	6250	6250			
		P. aeruginosa	6250	6250			
		E. coli	6250	6250			
		B. tequilensis	6250	6250			
		B. subtilis	6250	6250			
1-Hexyl-3-methylimidazolium bis(trifluormethylsu	$[C_6Mim][NTf_2]$	*S. aureus*	780	780	Agar disk diffusion / Agar well diffusion	Anti-adhesive activity[a]	[4]
		K. pneumoniae	12500	12500			
		S. typhimurium	12500	12500			

lfonyl)imide		*P. aeruginosa*	25000	25000			
		E. coli	6250	6250			
		B. tequilensis	6250	6250			
		B. subtilis	3120	3120			
1-Hexyl-3-methylimidazolium tosylate	$[C_6Mim][PTS]$	*S. aureus*	6250	6250	Agar disk diffusion / agar well diffusion	Anti-adhesive activity[a]	[4]
		K. pneumoniae	6250	6250			
		S. typhimurium	25000	25000			
		P. aeruginosa	12500	12500			
		E. coli	3120	12500			
		B. tequilensis	6250	12500			
		B. subtilis	12500	12500			
1-Hexyl-3-methylimidazolium nitrate	$[C_6Mim][NO_3]$	*S. aureus*	780	780	Agar disk diffusion / agar well diffusion	Anti-adhesive activity[a]	[4]
		K. pneumoniae	3120	3120			
		S. typhimurium	3120	3120			
		P. aeruginosa	6250	6250			
		E. coli	780	780			
		B. tequilensis	1560	1560			
		B. subtilis	3120	3120			
1-Octyl-3-methylimidazolium chloride	$[C_8Mim][Cl]$	*S. aureus* ATCC 29213	722 µM (MBEC 2708 µM)	>1444 µM	Broth microdilution, MBEC assay		[5]
		E-MRSA 15	722 µM (MBEC 2708 µM)	>1444 µM			
		MRSA (clinical strain 201)	1444 µM (MBEC 21666 µM)	>1444 µM			
		S. epidermidis ATCC 12228	361 µM	1444 µM			
		S. epidermidis ATCC 35984	722 µM (MBEC 10833 µM)	1444 µM			
		E. coli NCTC 8196	722 µM (MBEC 21666 µM)	1444 µM			
		P. aeruginosa PA01	>1444 µM (MBEC 21666 µM)	>1444 µM			
		K. aerogenes NCTC 7427	1444 µM (MBEC 43331 µM)	>1444 µM			
		B. cenocepacia J2315	>1444 µM	>1444 µM			

			(MBEC 43331 µM)				
		P. mirabilis NCTC 12442	1444 µM (MBEC 43331 µM)	>1444 µM			
		C. tropicalis NCTC 7393	1444 µM (MBEC >43331 µM)	>1444 µM			
1-Hexyl-2,3-dimethylimidazolium tetrafluoroborate	$[C_6MMim][BF_4]$	*S. aureus*	3120	3120	Agar disk diffusion / agar well diffusion	Anti-adhesive activity[a]	[4]
		K. pneumoniae	6250	6250			
		S. typhimurium	12500	12500			
		P. aeruginosa	3120	6250			
		E. coli	1560	3120			
		B. tequilensis	12500	12500			
		B. subtilis	6250	6250			
N-Butylpyridinium nitrate	$[C_4Py][NO_3]$	*S. aureus*	6250	6250	Agar disk diffusion / agar well diffusion	Anti-adhesive activity[a]	[4]
		K. pneumoniae	50000	50000			
		S. typhimurium	50000	50000			
		P. aeruginosa	6250	12500			
		E. coli	25000	25000			
		B. tequilensis	50000	50000			
		B. subtilis	12500	25000			
N-Hexylpyridinium nitrate	$[C_6Py][NO_3]$	*S. aureus*	1560	1560	Agar disk diffusion / agar well diffusion	Best anti-adhesive activity among the ILs tested in the current work.	[4]
		K. pneumoniae	6250	6250			
		S. typhimurium	6250	6250			
		P. aeruginosa	3120	3120			
		E. coli	1560	1560			
		B. tequilensis	3120	6250			
		B. subtilis	3120	3120			
N-Octylpyridinium bromide	$[C_8Py][Br]$	*M. luteus* ATCC 9341	R		Broth microdilution	R, resistant at the highest concentration tested (256 µg mL^{-1}).	[6]
		S. epidermidis ATCC155-1	940 µM				
		S. aureus ATCC 25178	R				
		E. coli ATCC 27325	R				
		K. pneumonia ATCC 9721	R				
		P. aeruginosa ATCC 9721	R				

		C. albicans ATCC10231	R				
		B. subtilis ATCC6633	R				
N-Decylpyridinium bromide	[C_{10}Py][Br]	*M. luteus* ATCC 9341	R		Broth microdilution	R, resistant at the highest concentration tested (256 µg mL^{-1}).	[6]
		S. epidermidis ATCC155-1	428 µM				
		S. aureus ATCC 25178	428 µM				
		E. coli ATCC 27325	428 µM				
		K. pneumonia ATCC 9721	R				
		P. aeruginosa ATCC 9721	R				
		C. albicans ATCC10231	R				
		B. subtilis ATCC6633	428 µM				
N-Butyl-*N*-methylpyrrolidinium chloride	[C_4C_1Pyr][Cl]	*L. monocytogenes* ATCC13932	5000		Broth microdilution		[1]
		B. cereus ATCC 11778	5000				
		S. aureus ATCC 6538	5000				
		E. faecalis ATCC 19433	5000				
		L. sakei ATCC 15521	3333				
		L. lactis ATCC 19435	4167				
		S. typhimurium ATCC 14028	>5000				
		E. coli ATCC 25922	5000				
		C. freundii ATCC 27853	>5000				
N-Butyl-*N*-methylpyrrolidinium bromide	[C_4C_1Pyr][Br]	*S. aureus*	64505 µM		Broth microdilution		[7]
		E. coli	83333 µM				
N-Hexyl-*N*-methylpyrrolidinium bromide	[C_6C_1Pyr][Br]	*S. aureus*	14920 µM		Broth microdilution		[7]
		E. coli	2988 µM				
N-Octyl-*N*-methylpyrrolidinium bromide	[C_8C_1Pyr][Br]	*S. aureus*	1808 µM		Broth microdilution		[7]
		E. coli	1899 µM				
N-Decyl-*N*-methylpyrrolidinium bromide	[$C_{10}C_1$Pyr][Br]	*S. aureus*	559 µM		Broth microdilution		[7]
		E. coli	622 µM				
Triethylmethylammonium chloride	[$C_{2,2,2,1}$N][Cl]	*L. monocytogenes* ATCC13932	>5000		Broth microdilution		[1]

		B. cereus ATCC 11778	>5000				
		S. aureus ATCC 6538	>5000				
		E. faecalis ATCC 19433	>5000				
		L. sakei ATCC 15521	>5000				
		L. lactis ATCC 19435	>5000				
		S. typhimurium ATCC 14028	>5000				
		E. coli ATCC 25922	>5000				
		C. freundii ATCC 27853	>5000				
Tetraethylammonium bromide	$[C_{2,2,2,2}N][Br]$	*E. coli* ATCC 25922	>5000 µM		Broth microdilution	*E. coli* TEM CTX M9, CTX M2, and AmpC MOX2 are ampicillin-resistant strains.	[2]
		E. coli TEM CTX M9	>5000 µM				
		E. coli CTX M2	2500 µM				
		E. coli AmpC MOX2	>5000 µM				
		K. pneumoniae (clinical isolate)	>5000 µM				
		S. aureus ATCC 25293	2500 µM				
		S. epidermidis (clinical isolate)	>5000 µM				
		E. faecalis (clinical isolate)	>5000 µM				
Di(2-hydroxyethyl)butyl methylammonium bromide	$[(HOC_2)_2C_{4,1}N][Br]$	*B. subtilis* ATCC 6633	>500		Broth microdilution		[8]
		M. smegmatis ATCC 607	N.A.				
		K. pneumonia ATCC 9997	N.A.				
		E. faecalis ATCC 29212	>500				
		VRE ATCC 51299	>500				
		S. aureus	N.A.				
		MRSA CIP 106760	N.A.				
		E. coli ATCC 25922	N.A.				
		P. aeruginosa ATCC 27853	N.A.				
		C. albicans ATCC 10231	N.T.				
		S. cerevisiae ATCC 2601	N.T.				
Cholinium chloride	[Chol][Cl]	*E. coli* ATCC 25922	>5000 µM		Broth microdilution	*E. coli* TEM CTX M9, CTX M2, and AmpC MOX2 are ampicillin-resistant strains.	[2]
		E. coli TEM CTX M9	>5000 µM				
		E. coli CTX M2	>5000 µM				
		E. coli AmpC MOX2	>5000 µM				
		K. pneumoniae (clinical isolate)	>5000 µM				
		S. aureus ATCC 25293	2500 µM				

		S. epidermidis (clinical isolate)	>5000 µM				
		E. faecalis (clinical isolate)	>5000 µM				
Gentamycin		*S. typhimurium* ATCC 14028	0.25		Broth microdilution		[9]
		E. coli ATCC 25922	0.25				
		C. freundii ATCC 27853	1				
		B. subtilis KCTC1914	1				
		S. typhimurium KCTC1926	0.5				
Kanamycin		*S. aureus* 209 KCTC1916			Broth microdilution		[9]
		S. aureus R209 KCTC1928					
		E. coli KCTC1924					
		B. subtilis KCTC1914					
		S. typhimurium KCTC1926					
Fuconazole		*C. tropicalis* 17A	0.125 (MBEC 4)		Broth microdilution	The clinical isolates 72A, 72P, and 94P are resistant to fluconazole, amphotericin B, voriconazole and anidulafungin.	[10]
		C. tropicalis 57A	0.125 (MBEC 64)				
		C. tropicalis 72A	128 (MBEC 8)				
		C. tropicalis 72P	128 (MBEC 128)				
		C. tropicalis 94P	64 (MBEC 32)				
		C. tropicalis 102A	0.125 (MBEC 128)				
Colistin		*E. coli* ATCC 25922	2		Broth microdilution		[11]
		P. aeruginosa ATCC 27853	1				
		K. pneumonia ATCC BAA-1705	2				
		A. baumannii AB01	4				
Vancomycin		*B. subtilis* ATCC 6633	<0.48		Broth microdilution		[8]
		K. pneumonia ATCC 9997	15.62				
		E. faecalis ATCC 29212	1.95				
		VRE ATCC 51299	3.91				
		S. aureus	7.82				
		MRSA CIP 106760	3.91				
Rifampicin		*M. smegmatis* ATCC 607	<0.48		Broth microdilution		[8]

		E. coli ATCC 25922	0.98				
Norfloxacin		*P. aeruginosa* ATCC 27853	<0.48		Broth microdilution		[8]
Amphotericin B		*C. albicans* ATCC 10231	<0.48		Broth microdilution		[8]
		S. cerevisiae ATCC 2601	<0.48				

[*] IZ, inhibition zone; MIC, minimum inhibitory concentration; MBC, minimum bactericidal concentration; MBEC, minimum biofilm eradication concentration; MRSA, methicillin-resistant *S. aureus*; N.A., not active; N.D., not determined; N.T., not tested; VRE, vancomycin-resistant *E. faecalis*.

[a] Anti-adhesive activity varies depending on the species.

Table S2. Antimicrobial activity of API-ILs[*]

**IL**	**Acronym**	**Species**	**IZ, mm**	**MIC µg mL^{-1}**	**MBC, µg mL^{-1}**	**Method**	**Notes**	**Ref.**
1-Ethyl-3-methylimidazolium ampicillinate	[C$_2$Mim][Amp]	*E. coli* ATCC 25922		>5000 µM		Broth microdilution	*E. coli* TEM CTX M9, CTX M2, and AmpC MOX2 are ampicillin-resistant strains.	[2]
		E. coli TEM CTX M9		>5000 µM				
		E. coli CTX M2		>5000 µM				
		E. coli AmpC MOX2		>5000 µM				
		K. pneumoniae (clinical isolate)		>5000 µM				
		S. aureus ATCC 25293		>5000 µM				
		S. epidermidis (clinical isolate)		>5000 µM				
		E. faecalis (clinical isolate)		>5000 µM				
1-(2-Hydroxyethyl)-3-methylimidazolium ampicillinate	[HOC$_2$Mim][Amp]	*E. coli* ATCC 25922		5000 µM		Broth microdilution	*E. coli* TEM CTX M9, CTX M2, and AmpC MOX2 are ampicillin-resistant strains.	[2]
		E. coli TEM CTX M9		>5000 µM				
		E. coli CTX M2		>5000 µM				
		E. coli AmpC MOX2		5000 µM				
		K. pneumoniae (clinical isolate)		>5000 µM				
		S. aureus ATCC 25293		>5000 µM				
		S. epidermidis (clinical isolate)		2500 µM				

		E. faecalis (clinical isolate)		5000 µM				
Tetraethylammonium ampicillinate	$[C_{2,2,2,2}N]$[Amp]	*E. coli* ATCC 25922		>5000 µM		Broth microdilution	*E. coli* TEM CTX M9, CTX M2, and AmpC MOX2 are ampicillin-resistant strains.	[2]
		E. coli TEM CTX M9		>5000 µM				
		E. coli CTX M2		>5000 µM				
		E. coli AmpC MOX2		>5000 µM				
		K. pneumoniae (clinical isolate)		>5000 µM				
		S. aureus ATCC 25293		>5000 µM				
		S. epidermidis (clinical isolate)		>5000 µM				
		E. faecalis (clinical isolate)		>5000 µM				
Cholinium ampicillinate	[Chol][Amp]	*E. coli* ATCC 25922		>5000 µM		Broth microdilution	*E. coli* TEM CTX M9, CTX M2, and AmpC MOX2 are ampicillin-resistant strains.	[2]
		E. coli TEM CTX M9		>5000 µM				
		E. coli CTX M2		>5000 µM				
		E. coli AmpC MOX2		>5000 µM				
		K. pneumoniae (clinical isolate)		>5000 µM				
		S. aureus ATCC 25293		>5000 µM				
		S. epidermidis (clinical isolate)		>5000 µM				
		E. faecalis (clinical isolate)		>5000 µM				
Nalidixic acid		*E. coli* BW25113 (wild-type)	11			Disk diffusion test, 10 µg per disk	Deletions ΔrfaC, ΔrfaL, and ΔrfaG affect the cell surface hydrophobicity and membrane permeability.	[12]
		E. coli JW3596 (ΔrfaC)	20					
		E. coli JW3597 (ΔrfaL)	11					
		E. coli JW3606 (ΔrfaG)	18					
Ampicillin sodium		*S. aureus* ATCC 6538		27 µM		Broth microdilution	*E. coli* TEM CTX	[2,

salt		*S. aureus* ATCC 25293		5 µM			M9, CTX M2, and AmpC MOX2 are ampicillin-resistant strains.	13]
		S. epidermidis (clinical isolate)		50 µM				
		E. coli O157:H7 ATCC 43895		12 µM				
		E. coli ATCC 25922		50 µM				
		E. coli TEM CTX M9		>5000 µM				
		E. coli CTX M2		>5000 µM				
		E. coli AmpC MOX2		>5000 µM				
		E. faecium ATCC 49474		17 µM				
		E. faecalis (clinical isolate)		50 µM				
		K. pneumonia ATCC 4352		20 µM				
		K. pneumoniae (clinical isolate)		2500 µM				
Benzalkonium chloride		*S. aureus* ATCC 6538		2 ppm	62.5 ppm	Tube dilution, broth microdilution		[9, 14]
		MRSA ATCC 43300		2 ppm	31.2 ppm			
		S. aureus 209 KCTC1916		8				
		S. aureus R209 KCTC1928		8				
		E. faecium ATCC 49474		4 ppm	31.2 ppm			
		E. coli ATCC25922		8 ppm	62.5 ppm			
		M. luteus ATCC 9341		4 ppm	31.2 ppm			
		S. epidermidis ATCC 12228		2 ppm	16 ppm			

		K. pneumonia ATCC 4352		4 ppm	31.2 ppm			
		B. subtilis KCTC1914		8				
		C. albicans ATCC 10231		8 ppm	16 ppm			
		R. rubra PhB		8 ppm	31.2 ppm			
		S. mutans PCM		2 ppm	16 ppm			
Didecyldimethylammonium chloride		*S. aureus* ATCC 6538		2 ppm	31.2 ppm	Tube dilution		[14]
		MRSA ATCC 43300		2 ppm	31.2 ppm			
		E. faecium ATCC 49474		4 ppm	31.2 ppm			
		E. coli ATCC25922		8 ppm	31.2 ppm			
		M. luteus ATCC 9341		2 ppm	31.2 ppm			
		S. epidermidis ATCC 12228		2 ppm	31.2 ppm			
		K. pneumonia ATCC 4352		4 ppm	16 ppm			
		C. albicans ATCC 10231		8 ppm	16 ppm			
		R. rubra PhB		4 ppm	31.2 ppm			
		S. mutans PCM		2 ppm	16 ppm			

* IZ, inhibition zone; MIC, minimum inhibitory concentration; MBC, minimum bactericidal concentration; MRSA, methicillin-resistant *S. aureus*.

Table S3. Antimicrobial activity of dicationic ILs*

IL	Acronym	Species	IZ, mm	MIC, μg mL^{-1}	MBC, μg mL^{-1}	Method	Ref.
1,1'-(Propane-1,3-diyl)bis(3-methyl-1H-imidazol-3-ium) dibromide	($[C_1Im]$-C_3-$[C_1Im]$)$[Br]_2$	*S. epidermidis* ATCC 35984		>10000		Broth microdilution	[15]
		S. aureus ATCC 6538		>10000			
		E. coli ATCC 25922		10000			
		P. aeruginosa ATCC 27853		>10000			
		E. faecalis ATCC		>10000			

		29212					
1,1'-Butane-1,4-diyl)bis(3-butyl-1H-imidazol-3-ium) dibromide	([C_4Im]-C_4-[C_4Im])[Br]$_2$	*S. epidermidis* ATCC 35984		5000		Broth microdilution	[15]
		S. aureus ATCC 6538		>10000			
		E. coli ATCC 25922		>10000			
		P. aeruginosa ATCC 27853		>10000			
		E. faecalis ATCC 29212		10000			
1,1'-(Hexane-1,6-diyl)bis(3-methyl-1H-imidazol-3-ium) dibromide	([C_1Im]-C_6-[C_1Im])[Br]$_2$	*S. epidermidis* ATCC 35984		10000		Broth microdilution	[15]
		S. aureus ATCC 6538		10000			
		E. coli ATCC 25922		>10000			
		P. aeruginosa ATCC 27853		>10000			
		E. faecalis ATCC 29212		>10000			
1,1'-(Octane-1,8-diyl)bis(3-methyl-1H-imidazol-3-ium) dibromide	([C_1Im]-C_8-[C_1Im])[Br]$_2$	*S. sanguinis* 10556		79000 µM		Broth microdilution	[16]
		S. salivarius 13419		79000 µM			
		S. mutans UA159		39000 µM			
		S. gordonii DL1.1		39000 µM			
		S. uberis 13419		20000 µM			
		E. faecalis V583		79000 µM			
		P. aeruginosa PA14		79000 µM			
		S. epidermidis		79000 µM			
1,1'-(Octane-1,8-diyl)bis(3-methyl-1H-imidazol-3-ium) diascorbate	([C_1Im]-C_8-[C_1Im])[Asc]$_2$	*S. sanguinis* 10556		79000 µM		Broth microdilution	[16]
		S. salivarius 13419		39000 µM			
		S. mutans UA159		39000 µM			
		S. gordonii DL1.1		39000 µM			
		S. uberis 13419		39000 µM			

		E. faecalis V583		156000 µM			
		P. aeruginosa PA14		20000 µM			
		S. epidermidis		10000 µM			
1,1'-(Octane-1,8-diyl)bis(3-methyl-1H-imidazol-3-ium) diphenylalaninate	([C_1Mim]-C_8-[C_1Mim])[Phe]$_2$	*S. sanguinis* 10556		20000 µM		Broth microdilution	[16]
		S. salivarius 13419		20000 µM			
		S. mutans UA159		10000 µM			
		S. gordonii DL1.1		79000 µM			
		S. uberis 13419		20000 µM			
		E. faecalis V583		79000 µM			
		P. aeruginosa PA14		20000 µM			
		S. epidermidis		10000 µM			
1,1'-(Octane-1,8-diyl)bis(3-methyl-1H-imidazol-3-ium) dileucinate	([C_1Im]-C_8-[C_1Im])[Leu]$_2$	*S. sanguinis* 10556		39000 µM		Broth microdilution	[16]
		S. salivarius 13419		20000 µM			
		S. mutans UA159		10000 µM			
		S. gordonii DL1.1		10000 µM			
		S. uberis 13419		10000 µM			
		E. faecalis V583		79000 µM			
		P. aeruginosa PA14		20000 µM			
		S. epidermidis		20000 µM			
1,1'-(Octane-1,8-diyl)bis(3-methyl-1H-imidazol-3-ium) dihistidinate	([C_1Mim]-C_8-[C_1Mim])[His]$_2$	*S. sanguinis* 10556		20000 µM		Broth microdilution	[16]
		S. salivarius 13419		20000 µM			
		S. mutans UA159		20000 µM			
		S. gordonii DL1.1		10000 µM			
		S. uberis 13419		20000 µM			
		E. faecalis V583		79000 µM			
		P. aeruginosa PA14		20000 µM			
		S. epidermidis		10000 µM			
1,1'-(Octane-1,8-diyl)bis(3-methyl-1H-imidazol-3-ium)	([C_1Im]-C_8-[C_1Im])[Met]$_2$	*S. sanguinis* 10556		20000 µM		Broth microdilution	[16]
		S. salivarius 13419		20000 µM			
		S. mutans UA159		10000 µM			
		S. gordonii DL1.1		20000 µM			

dimethioninate		*S. uberis* 13419		20000 µM			
		E. faecalis V583		79000 µM			
		P. aeruginosa PA14		20000 µM			
		S. epidermidis		10000 µM			
1,1'-(Decane-1,10-diyl)bis(3-methyl-1H-imidazol-3-ium) dibromide	($[C_1Mim]$-C_{10}-$[C_1Mim]$)$[Br]_2$	*S. sanguinis* 10556		10000 µM		Broth microdilution	[16]
		S. salivarius 13419		5000 µM			
		S. mutans UA159		2000 µM			
		S. gordonii DL1.1		5000 µM			
		S. uberis 13419		2000 µM			
		E. faecalis V583		39000 µM			
		P. aeruginosa PA14		39000 µM			
		S. epidermidis		2000 µM			
1,1'-(Decane-1,10-diyl)bis(3-methyl-1H-imidazol-3-ium) diascorbate	($[C_1Im]$-C_{10}-$[C_1Im]$)$[Asc]_2$	*S. sanguinis* 10556		20000 µM		Broth microdilution	[16]
		S. salivarius 13419		20000 µM			
		S. mutans UA159		10000 µM			
		S. gordonii DL1.1		20000 µM			
		S. uberis 13419		10000 µM			
		E. faecalis V583		79000 µM			
		P. aeruginosa PA14		20000 µM			
		S. epidermidis		20000 µM			
1,1'-(Decane-1,10-diyl)bis(3-methyl-1H-imidazol-3-ium) diphenylalaninate	($[C_1Im]$-C_{10}-$[C_1Im]$)$[Phe]_2$	*S. sanguinis* 10556		10000 µM		Broth microdilution	[16]
		S. salivarius 13419		5000 µM			
		S. mutans UA159		5000 µM			
		S. gordonii DL1.1		5000 µM			
		S. uberis 13419		2000 µM			
		E. faecalis V583		5000 µM			
		P. aeruginosa PA14		20000 µM			
		S. epidermidis		5000 µM			
1,1'-(Decane-1,10-diyl)bis(3-methyl-1H-	($[C_1Im]$-C_{10}-$[C_1Im]$)$[Leu]_2$	*S. sanguinis* 10556		10000 µM		Broth microdilution	[16]
		S. salivarius 13419		5000 µM			
		S. mutans UA159		5000 µM			

imidazol-3-ium) dileucinate		*S. gordonii* DL1.1		5000 µM			
		S. uberis 13419		5000 µM			
		E. faecalis V583		10000 µM			
		P. aeruginosa PA14		20000 µM			
		S. epidermidis		2000 µM			
1,1'-(Decane-1,10-diyl)bis(3-methyl-1H-imidazol-3-ium) dihistidinate	($[C_1Im]$-C_{10}-$[C_1Im]$)$[His]_2$	*S. sanguinis* 10556		10000 µM		Broth microdilution	[16]
		S. salivarius 13419		5000 µM			
		S. mutans UA159		5000 µM			
		S. gordonii DL1.1		5000 µM			
		S. uberis 13419		2000 µM			
		E. faecalis V583		20000 µM			
		P. aeruginosa PA14		20000 µM			
		S. epidermidis		5000 µM			
1,1'-(Decane-1,10-diyl)bis(3-methyl-1H-imidazol-3-ium) dimethioninate	($[C_1Im]$-C_{10}-$[C_1Im]$)$[Met]_2$	*S. sanguinis* 10556		10000 µM		Broth microdilution	[16]
		S. salivarius 13419		5000 µM			
		S. mutans UA159		5000 µM			
		S. gordonii DL1.1		5000 µM			
		S. uberis 13419		5000 µM			
		E. faecalis V583		20000 µM			
		P. aeruginosa PA14		20000 µM			
		S. epidermidis		2000 µM			
1,1'-(Hexane-1,6-diyl)bis(3-butyl-1H-imidazol-3-ium) dibromide	($[C_4Im]$-C_6-$[C_4Im]$)$[Br]_2$	*S. epidermidis* ATCC 35984		2500		Broth microdilution	[15]
		S. aureus ATCC 6538		5000			
		E. coli ATCC 25922		2500			
		P. aeruginosa ATCC 27853		>10000			
		E. faecalis ATCC 29212		10000			
1,1'-(Hexane-1,6-	($[C_1Pyr]$-C_6-	*S. epidermidis*		10000		Broth microdilution	[15]

diyl)bis(1-methylpyrrolidin-1-ium) dibromide	$[C_1Pyr])[Br]_2$	ATCC 35984					
		S. aureus ATCC 6538		>10000			
		E. coli ATCC 25922		>10000			
		P. aeruginosa ATCC 27853		>10000			
		E. faecalis ATCC 29212		>10000			
3,3'-(((Oxybis(ethane-2,1-diyl))bis(oxy))bis(ethane-2,1-diyl))bis(1-methyl-1H-imidazol-3-ium) dibromide	$([C_1Im]-C_8O_3-[C_1Im])[Br]_2$	*S. epidermidis* ATCC 35984		>10000		Broth microdilution	[15]
		S. aureus ATCC 6538		>10000			
		E. coli ATCC 25922		>10000			
		P. aeruginosa ATCC 27853		>10000			
		E. faecalis ATCC 29212		>10000			
Erythromycin		*S. aureus*	24	0.23	0.23	Disk diffusion (30 µg per well) ; broth microdilution	[17]
		E. coli	27	0.23	0.23		
		K. pneumoniae	26	0.23	0.23		
		P. aeruginosa	25	0.23	0.23		
		P. vulgaris	32	0.23	0.23		
Nalidixic acid		*S. aureus*	22	0.23	0.23	Disk diffusion (30 µg per well) ; broth microdilution	[17]
		E. coli	22	0.23	0.23		
		K. pneumoniae	27	0.23	0.23		
		P. aeruginosa	21	0.23	0.23		
		P. vulgaris	24	0.23	0.23		
Amikacin		*S. aureus*	19	0.23	0.23	Disk diffusion (30 µg per well) ; broth microdilution	[17]
		E. coli	20	0.23	0.23		
		K. pneumoniae	19	0.23	0.23		
		P. aeruginosa	17	0.23	0.23		
		P. vulgaris	17	0.23	0.23		

* IZ, inhibition zone; MIC, minimum inhibitory concentration; MBC, minimum bactericidal concentration; MRSA, methicillin-resistant *S. aureus*.

References

1. Weyhing-Zerrer, N.; Kalb, R.; Oßmer, R.; Rossmanith, P.; Mester, P., Evidence of a reverse side-chain effect of tris(pentafluoroethyl)trifluorophosphate [FAP]-based ionic liquids against pathogenic bacteria. *Ecotoxicol. Environ. Saf.* **2018,** 148, 467-472.
2. Ferraz, R.; Teixeira, V.; Rodrigues, D.; Fernandes, R.; Prudêncio, C.; Noronha, J. P.; Petrovski, Ž.; Branco, L. C., Antibacterial activity of ionic liquids based on ampicillin against resistant bacteria. *RSC Adv.* **2014,** 4, (9), 4301-4307.
3. Hajfarajollah, H.; Mokhtarani, B.; Noghabi, K. A.; Sharifi, A.; Mirzaei, M., Antibacterial and antiadhesive properties of butyl-methylimidazolium ionic liquids toward pathogenic bacteria. *RSC Adv.* **2014,** 4, (80), 42751-42757.
4. Anvari, S.; Hajfarajollah, H.; Mokhtarani, B.; Enayati, M.; Sharifi, A.; Mirzaei, M., Antibacterial and anti-adhesive properties of ionic liquids with various cationic and anionic heads toward pathogenic bacteria. *J. Mol. Liq.* **2016,** 221, 685-690.
5. Carson, L.; Chau, P. K. W.; Earle, M. J.; Gilea, M. A.; Gilmore, B. F.; Gorman, S. P.; McCann, M. T.; Seddon, K. R., Antibiofilm activities of 1-alkyl-3-methylimidazolium chloride ionic liquids. *Green Chem.* **2009,** 11, (4), 492–497.
6. Cornellas, A.; Perez, L.; Comelles, F.; Ribosa, I.; Manresa, A.; Garcia, M. T., Self-aggregation and antimicrobial activity of imidazolium and pyridinium based ionic liquids in aqueous solution. *J. Colloid. Interface Sci.* **2011,** 355, (1), 164-171.
7. Qin, J.; Guo, J.; Xu, Q.; Zheng, Z.; Mao, H.; Yan, F., Synthesis of pyrrolidinium-type poly(ionic liquid) membranes for antibacterial applications. *ACS Appl. Mater. Interfaces* **2017,** 9, (12), 10504-10511.
8. Siopa, F.; Figueiredo, T.; Frade, R. F. M.; Neto, I.; Meirinhos, A.; Reis, C. P.; Sobral, R. G.; Afonso, C. A. M.; Rijo, P., Choline-based ionic liquids: Improvement of antimicrobial activity. *ChemistrySelect* **2016,** 1, (18), 5909-5916.
9. Demberelnyamba, D.; Kim, K.-S.; Choi, S.; Park, S.-Y.; Lee, H.; Kim, C.-J.; Yoo, I.-D., Synthesis and antimicrobial properties of imidazolium and pyrrolidinonium salts. *Bioorg. Med. Chem. Lett.* **2004,** 12, (5), 853-857.
10. Bergamo, V. Z.; Donato, R. K.; Dalla Lana, D. F.; Donato, K. J. Z.; Ortega, G. G.; Schrekker, H. S.; Fuentefria, A. M., Imidazolium salts as antifungal agents: strong antibiofilm activity against multidrug-resistant *Candida tropicalis* isolates. *Lett. Appl. Microbiol.* **2015,** 60, (1), 66-71.
11. Florio, W.; Rizzato, C.; Becherini, S.; Guazzelli, L.; D'Andrea, F.; Lupetti, A., Synergistic activity between colistin and the ionic liquids 1-methyl-3-dodecylimidazolium bromide, 1-dodecyl-1-methylpyrrolidinium bromide, or 1-dodecyl-1-methylpiperidinium bromide against Gram-negative bacteria. *J. Glob. Antimicrob Resist.* **2020,** 21, 99-104.
12. Gundolf, T.; Rauch, B.; Kalb, R.; Rossmanith, P.; Mester, P., Influence of bacterial lipopolysaccharide modifications on the efficacy of antimicrobial ionic liquids. *J. Mol. Liq.* **2018,** 271, 220-227.
13. Cole, M. R.; Li, M.; El-Zahab, B.; Janes, M. E.; Hayes, D.; Warner, I. M., Design, synthesis, and biological evaluation of β-lactam antibiotic-based imidazolium- and pyridinium-type ionic liquids. *Chem. Biol. Drug Des.* **2011,** 78, (1), 33-41.
14. Hough-Troutman, W. L.; Smiglak, M.; Griffin, S.; Matthew Reichert, W.; Mirska, I.; Jodynis-Liebert, J.; Adamska, T.; Nawrot, J.; Stasiewicz, M.; Rogers, R. D.; Pernak, J., Ionic liquids with dual biological function: sweet and anti-microbial, hydrophobic quaternary ammonium-based salts. *New J. Chem.* **2009,** 33, (1), 26-33.
15. Florio, W.; Becherini, S.; D'Andrea, F.; Lupetti, A.; Chiappe, C.; Guazzelli, L., Comparative evaluation of antimicrobial activity of different types of ionic liquids. *Mater. Sci. Eng., C* **2019,** 104, 109907.
16. Gindri, I. M.; Siddiqui, D. A.; Bhardwaj, P.; Rodriguez, L. C.; Palmer, K. L.; Frizzo, C. P.; Martins, M. A. P.; Rodrigues, D. C., Dicationic imidazolium-based ionic liquids: a new strategy for non-toxic and antimicrobial materials. *RSC Adv.* **2014,** 4, (107), 62594-62602.

17. Ganapathi, P.; Ganesan, K., Anti-bacterial, catalytic and docking behaviours of novel di/trimeric imidazolium salts. *J. Mol. Liq.* **2017,** 233, 452-464.
